# Evaluating energy balance and environmental footprint of sludge management in BRICS countries

**DOI:** 10.1016/j.wroa.2024.100255

**Published:** 2024-09-01

**Authors:** Zhenyao Wang, Xuan Li, Huan Liu, Jinhua Mou, Stuart J. Khan, Carol Sze Ki Lin, Qilin Wang

**Affiliations:** a Center for Technology in Water and Wastewater, School of Civil and Environmental Engineering, University of Technology, Ultimo, NSW, 2007, Australia; b School of Energy and Environment, City University of Hong Kong, Tat Chee Avenue, Kowloon, Hong Kong, PR China; c School of Civil Engineering, University of Sydney, NSW 2006, Australia

**Keywords:** Sludge management, BRICS countries, Life cycle assessment, Environmental impacts, Energy balance, Carbon neutrality

## Abstract

Climate change is driving global endeavours to achieve carbon neutrality and renewable energy expansion. Sludge, a nutrient-rich waste, holds energy potential yet poses environmental challenges that need proper management. We conducted a comprehensive life cycle assessment to evaluate the energy balance and environmental footprint of the most commonly used sludge management scenarios in BRICS countries, namely Brazil, Russia, India, China, and South Africa. Technologies such as incineration and anaerobic digestion with energy recovery units (i.e., cogeneration unit) maximize energy balance and minimize the environmental footprint, with incineration showing a superior performance. Shifting sludge management scenarios from the worst to the best can boost energy production by 1.4–98.4 times and cut the environmental footprint by 1.5–21.4 times. In 2050, these improvements could lead to a 98-fold boost in energy generation and a 25-fold drop in carbon emissions, according to the Announced Pledges Scenarios. Optimizing parameters such as volatile solids and anaerobic digestion efficiency further boosts energy output and minimizes the environmental footprint. This study offers robust evidence to support sustainable sludge management and thus promote energy recovery and carbon neutrality goals, guide technological transitions, and inform policymaking for sustainable development.

## Highlights

• Commonly used BRICS sludge treatment routes were valued via life cycle assessment.
• Incineration with energy recovery best boosts energy and cuts carbon emissions.
• Best scenarios boost energy 1.4–98.4 times and cuts carbon footprint 1.5–21.4 times.
• The optimal sludge disposal can save 97 % of carbon emissions from transport sector.
• Proper sludge management contributing to the achievement of carbon neutrality.

## Graphical abstract

**Figure:**
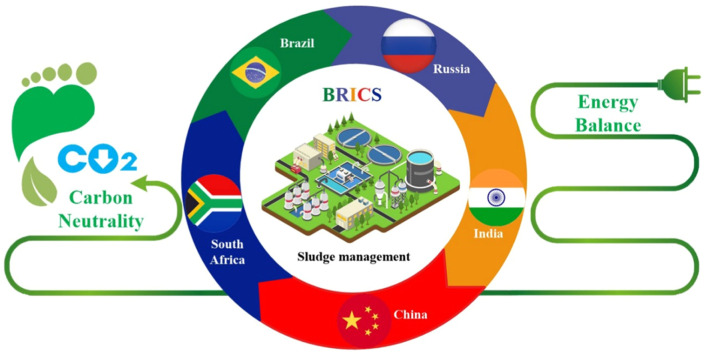

## Introduction

The need to respond to global climate change has inspired a drive toward carbon neutrality and renewable energy development. Sludge, a waste product generated by sewage treatment plants, is increasingly recognized as a renewable energy source due to its high energy content (3.54 kWh/kg dry sludge) and availability at massive quantities of production (75–100 million tons (Mt) globally in 2022) (Apollo, 2022; Wang et al., 2020). Rapid urbanization and population growth have directly boosted sludge generation, especially in densely populated countries such as India and China, leading to predictions that global sludge production will reach 130 Mt by 2030 (Apollo, 2022; Wang et al., 2023b). Many countries have set ambitious goals to halve their carbon emissions by 2030 and to achieve carbon neutrality by 2050 (Du et al., 2023). Consequently, sludge is proposed as a promising renewable energy source and an alternative to fossil fuels. However, sludge contains diverse pollutants (e.g., heavy metal ions, triclosan, per- and polyfluoroalkyl substances, virus, antibiotic resistance genes, etc.) that require proper management or treatment (Bagheri et al., 2023; Li et al., 2021; Wang et al., 2023c; Zhou et al., 2024). Globally, sludge management involves the reduction of moisture content through processes such as thickening, dewatering, and thermal drying (Teoh and Li, 2020); stabilization to decrease the organic content through anaerobic digestion, composting, and incineration (Fang et al., 2019); and, ultimately, disposal via landfills and land application (Tang et al., 2022). However, most of these processes are energy-intensive and negatively impact the environment by contributing to global warming, terrestrial acidification, freshwater eutrophication, human carcinogenic toxicity, and fossil resource scarcity (Chen et al., 2022; Mayer et al., 2021). An understanding of the energy balance and environmental impact of sludge management is thus urgently needed to inform decision-making and support energy and environmental sustainability.

**Fig. 1:**
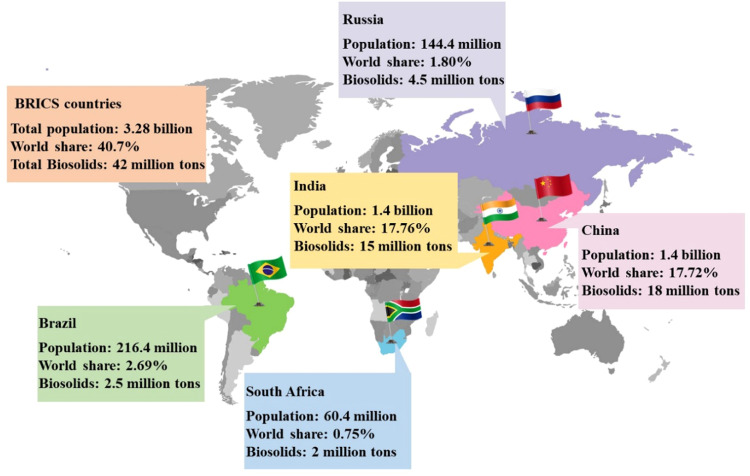
Population size and annual biosolids (dry sludge) production of the BRICS countries (**B**razil, **R**ussia, **I**ndia, **C**hina, and **S**outh Africa, sourced from http://infobrics.org/). The world share indicates each BRICS country's proportion of the global population.

Studies on sludge management have generally focused on assessing the environmental impacts of laboratory technological inventions and improvements such as sludge pre-treatment, advanced oxidation processes, hydrothermal carbonization, and co-combustion with coal (Hao et al., 2020; Medina-Martos et al., 2020), while neglecting real-life application scenarios. Moreover, densely populated developing countries tend to generate sludge in significantly larger amounts than developed countries (e.g., 18 Mt/year in China versus 6.5 Mt/year in the United States) (Altieri et al., 2023; Sauve and Van Acker, 2020). In particular, 40.7 % (3.28 billion) of the global population lives in the BRICS countries, namely, Brazil, Russia, India, China, and South Africa, which account for 42 % to 56 % of global sludge production (Fig. 1). Additionally, the BRICS countries collectively are responsible for approximately 41.8 % of all global carbon dioxide emissions, highlighting the urgent need to develop renewable energy sources (Fang and Chang, 2023). However, limited attention has been directed towards BRICS countries in assessments of the energy balance and environmental impact of sludge management. Recently, BRICS countries have focused on fostering economic growth, promoting sustainable development, and leveraging their potential to address global challenges and thus achieve the United Nations Sustainable Development Goals (Nguyen and Khominich, 2023). Therefore, it is necessary to comprehensively evaluate the energy balance and environmental footprint of sludge management, particularly in densely populated developing countries such as those within the BRICS alliance.

**Fig. 2:**
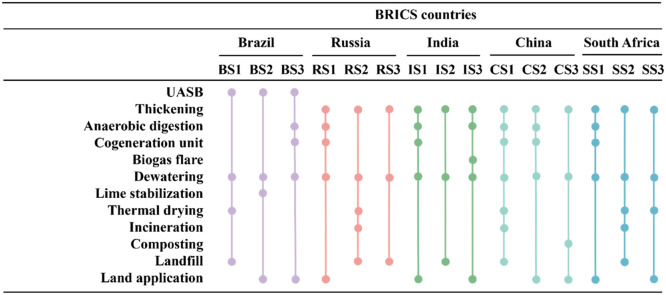
Mainstream sludge treatment and disposal processes used in the BRICS countries. For each country, three mainstream sludge management scenarios were considered and labelled accordingly. For instance, BS1, BS2, and BS3 represent three different sludge management scenarios that are commonly used in Brazil (sourced from a governmental report, see Methods). For each sludge management scenario, the dots are connected sequentially from top to bottom, representing the sequence of steps in the process. UASB represents the up-flow anaerobic sludge blanket.

In this study, we focused on assessing the energy balance and environmental impact of mainstream sludge management scenarios in the BRICS countries (Fig. 2 and detailed in Figs. S1–S5). A comprehensive life cycle assessment was conducted to address the following questions: 1) What is the best sludge management scenario for each country in terms of the energy balance and environmental impact? 2) What level of carbon emission reduction can be anticipated in the future if the best sludge management scenarios are adopted? 3) How do crucial parameters affect the energy balance and environmental impact of sludge management scenarios? Notably, the functional unit for this assessment was 1 t of dry sludge. By providing solid scientific evidence, our study will enable technological transitions and inform policymaking in developing countries, enabling them to maximize energy recovery while minimizing environmental contamination and, ultimately, achieving sustainable development.

## Results

### Energy balance and environmental impact of sludge management scenarios in Brazil

**Fig. 3:**
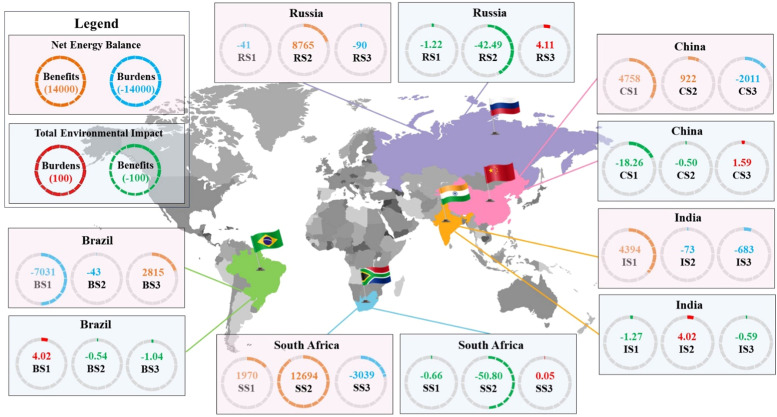
Net energy balance and total environmental impact of sludge treatment and disposal scenarios in BRICS countries. The total environmental impact of the sludge management scenarios was calculated using the sum of the normalized impact categories (see Methods). Negative total environmental impact values denote environmental benefits, while positive values represent environmental burdens. For the net energy balance, positive values indicate energy production, whereas negative values represent energy consumption. BS1–3, RS1–3, IS1–3, CS1–3, and SS1–3 denote the selected sludge management scenarios in Brazil, Russia, India, China, and South Africa, respectively. The specific processes and mass flow of each sludge management scenario are outlined in Figs. 2, S1–S5. The total environmental impact and net energy balance were calculated based on the functional unit of 1 ton of dry sludge solids.

In our analysis of sludge management scenarios in Brazil, BS3 (UASB–Anaerobic digestion–Cogeneration unit–Dewatering–Land application) (Fig. 2) yielded the highest energy recovery of 2815 MJ, whereas both BS1 (UASB–Dewatering–Thermal drying–Landfill) and BS2 (UASB–Dewatering–Lime stabilisation–Land application) (Fig. 2) exhibited increased energy consumption of 7031 and 43 MJ, respectively (Fig. 3). The superior energy recovery performance of BS3 was likely to due to energy production by the cogeneration unit (Fig. S6a) (Ding et al., 2021; Pasciucco et al., 2023). Conversely, scenarios BS1 and BS2 lack energy recovery processes, and BS1 includes a particularly energy-intensive thermal drying step (Adibimanesh et al., 2023) that led to a higher energy consumption than BS2.

**Fig. 4:**
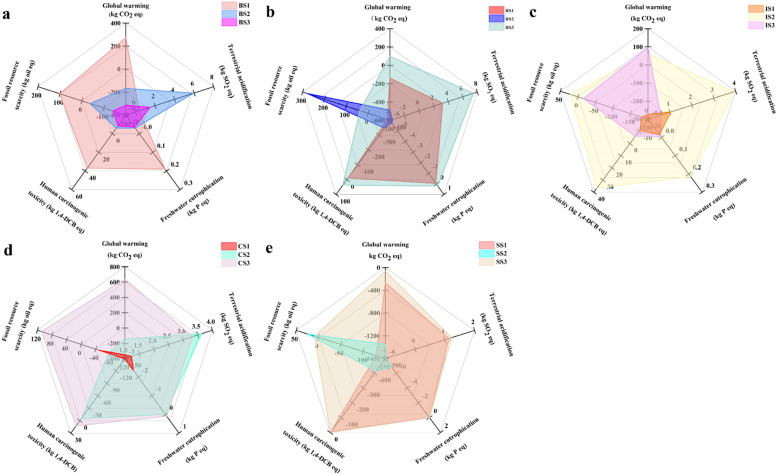
Comparison of the environmental impacts in various categories between sludge management scenarios used in BRICS countries. The results for environmental impact categories of the sludge management scenarios used in Brazil (a), Russia (b), India (c), China (d), and South Africa (e). BS1–3, RS1–3, IS1–3, CS1–3, and SS1–3 denote the sludge management scenarios adopted in the BRICS countries; the detailed processes are outlined in Fig. 2.

The superior energy production in BS3 was also associated with the lowest total environmental impact, as indicated by the highest environmental impact value of −1.04 (Fig. 3). BS3 exhibited predominant advantages in reducing carbon emissions (−314 kg CO_2_ equivalent (eq) saving), freshwater eutrophication (−0.036 kg phosphorus (P) eq), human carcinogenic toxicity (−8.61 kg 1,4-dichlorobenzene (1,4-DCB)), and fossil resource scarcity (−141.61 kg oil eq) (Fig. 4). These environmental benefits of BS3 stem from its inclusion of anaerobic digestion, cogeneration unit, and land application processes (Fig. S7c). Anaerobic digestion improves sludge quality by reducing the release of nutrients and untreated substances into the environment, thus mitigating freshwater eutrophication (Dere et al., 2012; Usman et al., 2012), and by stabilizing heavy metal ions (i.e., nickel, cadmium, and mercury), which contribute to human carcinogenic toxicity (Liew et al., 2022; Mayer et al., 2021). Moreover, the cogeneration unit enables energy recovery, thus reducing carbon emissions and fossil resource scarcity (Ding et al., 2021; Pasciucco et al., 2023). Land application takes advantage of nitrogen and phosphorus fertilizers present in sludge, reducing the use of chemically synthesized fertilizers and thus decreasing carbon emissions (Yang et al., 2023). In contrast, BS1 had the worst environmental impact value of 4.02 (Fig. 3), which was dominated by carbon emissions (273 kg CO_2_ eq), freshwater eutrophication (0.19 kg P eq), human carcinogenic toxicity (36.91 kg 1,4-DCB), and fossil resource scarcity (101.43 kg oil eq) (Fig. 4). These factors stem from the absence of sludge stability technology (e.g., anaerobic digestion, lime stabilization, or incineration) and the high amount of energy consumed by thermal drying (6640 MJ) (Adibimanesh et al., 2023). Notably, the terrestrial acidification impact of BS1 (0.37 kg SO_2_ eq) was lower than that of BS2 (5.94 kg SO_2_ eq); this was mainly due to the addition of lime (Fig. S7b) and consequently resulted in emissions of ammonia, sulfur oxides, and nitrogen oxides (Yoshida et al., 2018)

### Energy balance and environmental impact of sludge management scenarios in Russia

Among the sludge management scenarios used in Russia, RS2 (Thickening–Dewatering–Thermal drying–Incineration–Landfill) (Fig. 2) achieved a maximum energy production of 8765 MJ, while RS1 (Thickening–Anaerobic digestion–Cogeneration unit–Dewatering–Land application) and RS3 (Thickening–Dewatering–Landfill) (Fig. 2) exhibited increased energy consumption of 41 MJ and 90 MJ, respectively (Fig. 3). The notable energy recovery in RS2 can be primarily attributed to the inclusion of incineration process (Fig. S6b), which effectively decomposes organic matter in sludge while simultaneously generating heat and electricity (Liu et al., 2023). Although RS1 includes a cogeneration unit to enable energy recovery through anaerobic digestion, the energy recovered (3220 MJ) cannot offset the high energy consumption (3261 MJ) required to achieve a desirable temperature for anaerobic digestion (i.e., 35 °C) in Russia (annual average temperature of 4.9 °C). In RS3, the energy consumption stems from the thickening and dewatering processes, consistent with prior research, indicating that these processes have lower energy requirements than other sludge treatment processes such as anaerobic digestion and cogeneration unit (Wang et al., 2024a).

RS2 also emerged as the optimal sludge management scenario in terms of environmental burden mitigation, boasting a total environmental impact value of −42.49; accordingly, it outperformed RS1 (benefit = −1.22) by 35-fold. In contrast, RS3 imposed a severe environmental burden, with a total impact value of 4.11 (Fig. 3). RS2 substantially mitigated global warming, terrestrial acidification, freshwater eutrophication, and human carcinogenic toxicity, as indicated by respective values of −489 kg CO_2_ eq, −3.67 kg SO_2_ eq, −5.74 kg P eq, and −347.91 kg 1,4-DCB. These superior mitigation effects of RS2 can be attributed to the enhanced energy recovery due to the incineration process, enabling reductions in fossil-based fuel consumption and carbon emissions (Singh et al., 2020b). Furthermore, studies have shown that phosphorus in ash from sludge incineration has low bioavailability and that incineration releases nitrogen as exhaust fumes (Horttanainen et al., 2017), thus reducing freshwater eutrophication (Li et al., 2015). Incineration converts the heavy metal ions in sludge to more stable forms than those found in digested sludge, thus mitigating human carcinogenic toxicity (Horttanainen et al., 2017; Li et al., 2015). The fact that the heaviest environmental burden was imposed by RS3 can be attributed primarily to the absence of a sludge stabilization process; this lack facilitates the transfer of pollutants to the environment, thus increasing the environmental burden (Guo et al., 2023).

### Energy balance and environmental impact of sludge management scenarios in India

Among the scenarios used in India, IS1 (Thickening–Anaerobic digestion–Cogeneration unit–Dewatering–Land application) (Fig. 2) achieved the highest energy recovery, reaching 4394 MJ (Fig. 3). In contrast, IS2 (Thickening–Dewatering–Landfill) and IS3 (Thickening–Anaerobic digestion–Biogas flare–Dewatering–Land application) (Fig. 2) both increased the energy consumption by 73 MJ and 683 MJ, respectively (Fig. 3). Notably, IS1 and IS3 have similar processes other than the utilization of biogas (energy recovery in the cogeneration unit in IS1, biogas flaring in IS3). The vast disparity in the net energy balance between IS1 and IS3 can be attributed primarily to the energy-intensive nature of anaerobic digestion (Ding et al., 2021; Pasciucco et al., 2023); IS1 lacks energy recovery, while IS3 includes it (Fig. S6c).

IS1 achieved the greatest environmental benefit, with an impact value of −1.27 (Fig. 3). IS1 demonstrated the greatest advantages in terms of reducing greenhouse gas emissions (−274 kg CO_2_ eq), freshwater eutrophication (−0.016 kg P eq), human carcinogenic toxicity (−10.81 kg 1,4-DCB), and fossil resource scarcity (−182.73 kg oil eq) (Fig. 4). These mitigating effects can be ascribed to the improved sludge quality due to anaerobic digestion (Dad et al., 2019; Dong et al., 2013), a reduced reliance on fossil fuels due to enhanced energy recovery from the cogeneration unit, and the ability of land application to substitute for chemical fertilizer use (Yang et al., 2023) (Fig. S9). Moreover, terrestrial acidification was lower in IS1 than in IS2 (1.04 versus 3.96 kg SO_2_ eq), which may be attributable to the higher ammonia emissions caused by organic matter decomposition during the landfill disposal of untreated sludge in IS2 (Angouria-Tsorochidou et al., 2022) (Fig. 4). Furthermore, IS2 was found to exacerbate the total environmental impact burden, with an impact value of 4.02 (Fig. 3). Specifically, IS2 led to increases in terrestrial acidification (3.96 kg SO_2_ eq), freshwater eutrophication (0.21 kg P eq), human carcinogenic toxicity (36.67 kg 1,4-DCB), and fossil resource scarcity (21.39 kg oil eq) (Fig. 4). IS3 yielded the greatest carbon emissions (96.42 kg CO_2_ eq) due to the energy-intensive nature of anaerobic digestion (Ding et al., 2021; Pasciucco et al., 2023), which relies heavily on fossil-based fuel and consequently increases the atmospheric emission of greenhouse gases.

### Energy balance and environmental impact of sludge management scenarios in China

Among the scenarios used in China, CS1 (Thickening–Anaerobic digestion–Cogeneration unit–Dewatering–Incineration–Landfill) and CS2 (Thickening–Anaerobic digestion–Cogeneration unit–Dewatering–Land application) (Fig. 2) exhibited increased energy production, with CS1 (4758 MJ) outperforming CS2 (922 MJ) by 5.2 times (Fig. 3). In contrast, CS3 (Thickening–Dewatering–Composting–Land application) (Fig. 2) consumed 2011 MJ of energy (Fig. 3). The superiority of CS1 over CS2 can be attributed to the energy recovery process: although both scenarios include anaerobic digestion, only CS1 includes incineration (Fig. S6d). In CS3, energy consumption can be attributed primarily to composting, which is recognized as an energy-intensive process (Arias et al., 2021).

Both CS1 and CS2 effectively reduced the total environmental burden, with CS1 (−18.26) demonstrating a 36.5 times greater reduction in the impact value than CS2 (−0.50) (Fig. 3). CS1 exhibited superior performance in terms of mitigating carbon emissions (−350.27 kg CO_2_ eq), freshwater eutrophication (−2.38 kg P eq), and human carcinogenic toxicity (−149.68 kg 1,4-DCB). It also exhibited the smallest increase in the environmental burden related to terrestrial acidification (1.23 kg SO_2_ eq) (Fig. 4). Studies have shown that anaerobic digestion improves sludge quality (Dere et al., 2012; Usman et al., 2012), while incineration further reduces the nutrient content and stabilizes heavy metal ions (Horttanainen et al., 2017; Li et al., 2015); these factors contributed to the dominant role of CS1 in these impact categories. Furthermore, high energy recovery from sludge incineration leads to a reduced dependency on fossil fuel and consequently reduces the emission of environmental pollutants. Notably, CS2 mitigated fossil resource scarcity to a greater extent than CS1 (Fig. 4), and this can be attributed mainly to the land application of digested sludge, which provides nutrients to the soil (Fig. S10) and reduces the need for chemically synthesized fertilizers (Yang et al., 2023). CS3, the worst scenario, increased the environmental burden (1.59) (Fig. 4), with the strongest impacts on global warming (638.48 kg CO_2_ eq), freshwater eutrophication (0.09 kg P eq), human carcinogenic toxicity (12.17 kg 1,4-DCB), and fossil resource scarcity (109.24 kg oil eq). The composting process included in CS3 has been recognized as energy-intensive, leading to fossil fuel scarcity (Arias et al., 2021), and it contributes to the atmospheric emission of nitrogen and sulfur oxides, which contribute to global warming (Morsink-Georgali et al., 2022; Weihs et al., 2022). Simultaneously, the composting process facilitates the release of heavy metal ions (e.g., nickel, cadmium) and nutrients (e.g., nitrogen, phosphorus) into the environment, leading to human carcinogenic toxicity and freshwater eutrophication (Zheng et al., 2007). The release occurs due to microbial activity during the composting process, allowing heavy metal ions to enter soil and water environments. Human exposure occurs through multiple pathways, including direct contact with contaminated soil, consumption of crops grown in contaminated soil, and drinking water sourced from contaminated areas. Previous studies have reported that heavy metal ions (i.e., nickel, cadmium, and mercury) are highly associated with human carcinogenic toxicity (Liew et al., 2022; Mayer et al., 2021).

### Energy balance and environmental impact of sludge management scenarios in South Africa

Among the scenarios used in South Africa, SS2 (Thickening–Dewatering–Thermal drying–Incineration–Landfill) (Fig. 2) was identified as the most effective scenario for achieving energy recovery, with an energy production of 12,694 MJ, followed by SS1 (Thickening–Anaerobic digestion–Cogeneration unit–Dewatering–Land application) (Fig. 2) with an energy production of 1970 MJ (Fig. 3). These findings indicate that incineration and the cogeneration unit contribute to energy recovery, with incineration being most effective for energy production, aligning with previous studies (Zhao et al., 2023). SS2, which includes the same processes as RS2, exhibited a 1.45-fold higher energy production than RS2, which is probably attributable to a higher organic matter content in sludge (70 % versus 65 % in RS2). In contrast, SS3 (Thickening–Dewatering–Thermal drying–Land application) (Fig. 2) led to a substantial increase in energy consumption, reaching 3039 MJ (Fig. 3). This elevated energy consumption can be attributed primarily to thermal drying, which accounted for 97 % of the total energy consumption in SS3 (Fig. S6e).

SS2 achieved the best performance in terms of mitigating the environmental burden, with an impact value of −50.80, followed by SS1, which achieved a slight reduction in the total environmental burden (impact value = −0.66) (Fig. 3). SS2 led to reductions in the global warming, terrestrial acidification, freshwater eutrophication, and human carcinogenic toxicity, as demonstrated by values of −1348.6 kg CO_2_ eq, −0.98 kg SO_2_ eq, −6.85 kg P eq, and −412.83 kg 1,4-DCB eq, respectively (Fig. 4). The high energy recovery achieved during incineration, combined with a reduction in the nutrient content and stabilization of heavy metal ions (Horttanainen et al., 2017; Li et al., 2015), determined the mitigation of these impact categories in SS2. However, SS2 was less effective than SS1 in addressing fossil resource scarcity (Fig. 4). This is because the combination of energy recovery (cogeneration unit) and chemical fertilizer substitution (land application) in SS1 surpasses the energy recovery achieved via incineration (Fig. S11). SS3 represents the worst scenario, exhibiting a slight increase in the environmental burden (impact value = 0.05). It achieves a minimal reduction in carbon emissions (−62 kg CO_2_ eq) and human carcinogenic toxicity (−0.082 kg 1,4-DCB eq), while experiencing the maximum improvement in terrestrial acidification (1.23 kg SO_2_ eq) and freshwater eutrophication (0.019 kg P eq). Notably, these impact categories were dominated by thermal drying and land application (Fig. S11).

### Comparative analysis of the optimal sludge management strategies in the BRICS countries

In the preceding discussion, the most effective sludge management strategies for maximizing environmental benefits and energy recovery across Brazil, Russia, India, China, and South Africa were identified as BS3, RS2, IS1, CS1, and SS2, respectively. Specifically, the overall environmental benefits of optimal sludge management scenarios in the BRICS countries followed the order: South Africa (−50.80), Russia (−42.49), China (−18.26), India (−1.27), and Brazil (−1.04), with corresponding energy production of 12,694, 8765, 4758, 4394, and 2815 MJ, respectively, per 1 t of dry sludge. The maximum environmental benefits observed in South Africa can be attributed to the application of incineration at a high volatile solids content (up to 70 %, Table S1), accompanied by a net positive energy balance and a remarkable reduction in reliance on fossil fuels. Additionally, the incineration process reduced nutrient content (e.g., nitrogen and phosphorus) and stabilized metal ions (Horttanainen et al., 2017; Zhou et al., 2024). Although India had a higher volatile solids content in sewage sludge than South Africa (83 % versus 70 %, Table S1), it did not achieve higher environmental benefits and energy recovery. This discrepancy was attributed to the fact that incineration with energy recovery was more effective than anaerobic digestion with energy recovery in reducing pollutant emissions and increasing net energy balance (Horttanainen et al., 2017; Li et al., 2015). Notably, sludge management scenarios in Brazil exhibited the lowest environmental benefits and energy recovery compared to the other BRICS countries. The primary reason for this was the lower volatile content of sewage sludge (56 %, Table S1), which resulted in less biogas generation during the anaerobic digestion process and, consequently, less energy production for biogas valorization in the cogeneration unit.

Further analysis found that the optimal sludge management strategies in the BRICS countries can be categorized as follows: 1) anaerobic digestion with energy recovery and land application of sludge (i.e., BS3 and IS1); 2) incineration with energy recovery and landfill disposal (RS2 and SS2); and 3) anaerobic digestion with energy recovery, incineration with energy recovery and landfill disposal (CS1). Although the same sludge treatment process is applied, SS2 outperformed RS2 and IS1 outperformed BS3. This is likely due to the differences in sludge properties (i.e., solids content, volatile solids content of sewage sludge) and varying treatment standards (i.e., the permissible concentration values of heavy metal ions for land application of sludge). Scenarios in the last category (e.g., CS1) have not been widely adopted globally due to their complexity and cost (Zhao et al., 2023). Consequently, the sensitivity analysis in the subsequent section focuses exclusively on SS2 and IS1.

### Projections of energy balance and carbon emissions associated with sludge management in BRICS countries in 2023 and 2050

Increasing global turmoil has intensified the emphasis placed on greenhouse gas emissions (Liu and Rajagopal, 2019). Carbon emission, a primary concern of the sewage treatment industry, is a metric reportable to regulatory bodies; it incurs millions of dollars in annual taxes, such as those related to the Carbon Reduction Commitment (Mills et al., 2014). Most of the BRICS countries have committed to carbon emission reduction efforts: Brazil and South Africa have set a target of net-zero carbon emissions by 2050, China by 2060, and India by 2070 (Afrane et al., 2024; Das et al., 2023; Soterroni et al., 2023). The following projections of energy production and carbon emission under different sludge management scenarios in BRICS countries in 2023 and 2050 are based on the best-performing (i.e., BS3, RS2, IS1, CS1, and SS2) and worst-performing (i.e., BS1, RS3, IS3, CS3, and SS3) sludge management scenarios in each country, with consideration of increased sludge production along with urbanization and/or population growth and the countries’ carbon emission goals. Notably, the years 2023 and 2050 were chosen for the availability of accurate and current data and their significance in reflecting actual conditions and future carbon neutrality targets for most countries worldwide.

**Fig. 5:**
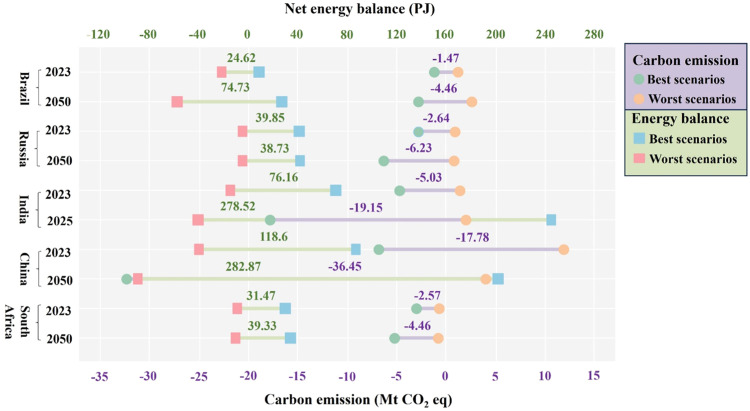
Energy balance and carbon emission projections for the best and worst sludge management scenarios in each BRICS country for the years 2023 and 2050 (according to the Announced Pledges Scenarios: see Methods). The green and purple values represent the differences between the best and worst sludge management scenarios in terms of the net energy balance and carbon emission, respectively, across the BRICS countries in the indicated years (for detailed calculations, see Methods). Positive net energy balance values represent energy production in the sludge management scenarios, whereas negative values represent energy consumption. Negative carbon emission values represent environmental benefits, whereas positive values represent environmental burdens. Notably, the projections of carbon emissions for 2050 are based on the predicted electricity generation sources in BRICS countries as derived from the International Energy Agency.

In 2023, the worst sludge management scenarios are estimated to exhibit substantial energy consumption, with the highest value in China (CS3: 36.2 PJ), followed by Brazil (BS1: 17.6 PJ), India (IS3: 10.2 PJ), South Africa (SS3: 6.1 PJ), and Russia (RS3: 0.4 PJ) (Fig. 5). Shifting to the best scenarios, the highest energy production is projected in China (CS1: 82.4 PJ), followed by India (IS1: 65.9 PJ), Russia (RS2: 39.4 PJ), South Africa (SS2: 25.4 PJ), and Brazil (BS3: 7 PJ). The energy benefits generated by shifting the worst scenarios to the best could satisfy the annual electricity demands of approximately 16.1, 5.3, 2.5, 2.2, and 1.4 million people in India, China, South Africa, Brazil, and Russia, respectively. Similar patterns are observed in the projections for 2050: the worst sludge management scenarios was predicted to have the highest energy consumption (CS3: 86.3 PJ) in China, followed by Brazil (BS1: 53.4 PJ), India (IS3: 37.5 PJ), and South Africa (SS2: 7.6 PJ). Transitioning to the best scenarios, India was predicted to have the highest energy production (IS1: 241.1 PJ), followed by China (CS1: 196.5 PJ), South Africa (SS2: 31.7 PJ), and Brazil (BS3: 21.4 PJ) (Fig. 5). The differences between the best and worst scenarios could meet the annual electricity needs of 59, 12.7, 6.6, and 3.1 million people in India, China, Brazil, and South Africa, respectively. Notably, in Russia, the variations of energy consumption (RS3) and energy production (RS2) in 2050 were comparable to those in 2023, mainly due to a projected decrease in population (Fig. S13b).

In terms of environmental impact, the worst sludge management scenario, CS3 in China, is estimated to contribute the largest amount of carbon emissions by 2023, reaching 11.48 Mt CO_2_ eq. This is estimated to be followed by the worst scenarios in India (IS3: 0.92 Mt CO_2_ eq), Brazil (BS1: 0.68 Mt CO_2_ eq), Russia (RS3: 0.44 Mt CO_2_ eq) and South Africa (SS3: −0.12 Mt CO_2_ eq) (Fig. 5). In contrast to other countries, the worst sludge management scenario in South Africa resulted in a slight reduction in carbon emissions in 2023. This reduction is likely due to the substitution of sludge for chemical fertilizer in agriculture applications (Fig. S11c). Transitioning to the best scenario, CS1 in China is projected to achieve the greatest carbon emission savings of 6.3 Mt CO_2_ eq, followed by India (IS1: 4.11 Mt CO_2_ eq), South Africa (SS2: 2.7 Mt CO_2_ eq), Russia (RS2: 2.2 Mt CO_2_ eq), and Brazil (BS3: 0.79 Mt CO_2_ eq) (Fig. 5). Shifting the worst scenarios into best scenarios is projected to reduce 55.8 %, 19.3 %, 18.2 %, 11 %, and 7.8 % of carbon emissions from the transport sectors in South Africa, China, India, Russia, and Brazil, respectively.

Similar patterns are observed in the projections for 2050, with CS3 in China expected to yield the greatest amount of carbon emissions of 3.65 Mt CO_2_ eq, followed by Brazil (BS1: 2.07 Mt CO_2_ eq), India (IS2: 1.59 Mt CO_2_ eq), Russia (RS3: 0.25 Mt CO_2_ eq). In contrast, the best sludge management scenario, CS1 in China, is projected to achieve the greatest reduction in carbon emissions (32.8 Mt CO_2_ eq), followed by India (IS1: 17.55 Mt CO_2_ eq), Russia (RS2: 5.98 Mt CO_2_ eq), South Africa (SS2: 4.79 Mt CO_2_ eq), and Brazil (BS3: 2.38 Mt CO_2_ eq). Although both the best and worst scenarios in South Africa are predicted to reduce carbon emissions, the best scenario (SS2: 4.79 Mt CO_2_ eq) demonstrates a reduction 14 times greater than that of the worst scenario (SS3: 0.34 Mt CO_2_ eq). The differences between the best and worst scenarios account for 96.6 %, 69.4 %, 39.6 %, 26 % and 23.5 % of carbon emissions from the transport sectors in South Africa, India, China, Russia and Brazil, respectively. Notably, the projected carbon emission reductions associated with RS3 and CS3 are lower in 2023 than in 2050, in contrast to the projections for other BRICS countries (Fig. 5); this difference may be due to expected reductions in population growth and/or urbanization rates (Fig. S13).

The above observations highlight the importance of selecting appropriate sludge management scenarios. Among the BRICS countries, shifting from the worst to the best scenario could enable China to achieve maximum energy and environmental benefits, with an estimated energy production of 118.6 PJ and carbon savings of 17.78 Mt CO_2_ eq in 2023 and respective increases to 282.87 PJ and 36.45 Mt CO_2_ eq by 2050 (Fig. 5). These projected benefits will enhance energy resources, reduce the fossil fuel dependency, and also mitigate sludge pollutants, stabilizing its state (e.g., heavy metal ions and nutrients).

### Sensitivity analysis of the optimal sludge management scenarios in BRICS countries

**Fig. 6:**
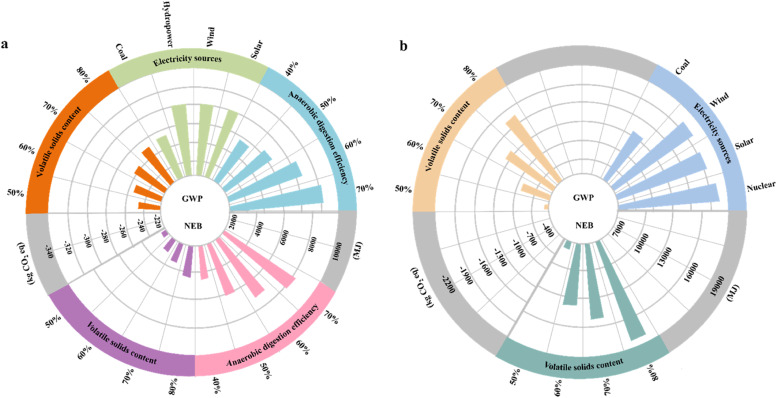
Sensitivity analysis of the optimal sludge management scenarios used in BRICS countries. (a) India, IS1 (Thickening–Anaerobic digestion–Cogeneration unit–Dewatering–Land application). (b) South Africa, SS2 (Thickening–Dewatering–Thermal drying–Incineration–Landfill). GWP and NEB represent the global warming potential (kg CO_2_ eq) and net energy balance (MJ), respectively. Regarding India, sensitivity analysis was used to explore parameters such as the volatile solids content (range: 50 %–80 %), anaerobic digestion efficiency (range: 40 %–70 %), and electricity sources (coal, hydropower, wind, and solar). Regarding South Africa, sensitivity analysis was used to explore parameters such as the volatile solids content (50 %–80 %) and electricity source (coal, wind, solar, and nuclear). The global warming potential is displayed in the upper left (Fig. 6a; India) and upper right (Fig. 6b; South Africa) halves of the circles. The lower left (Fig. 6a; India) and lower right (Fig. 6b; South Africa) halves of the circles illustrate the results of sensitivity analysis of the net energy balance.

The volatile solids content, anaerobic digestion efficiency, and electricity sources were selected as variables for sensitivity analysis (see Methods). Increasing the volatile solids content from 50 % to 80 % reduced carbon emissions by 27 kg CO_2_ eq in IS1 and 1295 kg CO_2_ eq in SS2 (Fig. 6). These reductions can be attributed primarily to increases in energy production of 1826 MJ in IS1 and 10,503 MJ in SS2 (Fig. 6). In IS1, energy recovery mainly occurred in the cogeneration unit (Fig. S16a), where an increase in biogas from anaerobic digestion elevated the energy output (Evangelisti et al., 2014a). In contrast, in SS2, the higher volatile solids content boosted the calorific value of sewage sludge (Zhang et al., 2021; Zhao et al., 2023), increasing energy production during incineration (Fig. S16c). These improvements have reduced reliance on fossil fuels during sludge management and, consequently, the emission of pollutants (i.e., carbon dioxides, nitrogen oxides, and sulfur oxides) into the atmosphere (Fig. S15). Similarly, in IS1, enhancing the anaerobic digestion efficiency from 40 % to 70 % led to a reduction in carbon emissions by 49 kg CO_2_ eq (Fig. 6a). This can be attributed to an increase in the conversion of organic matter to biogas, which is subsequently transformed into electricity and heat in the cogeneration unit (Fig. S16b), resulting in increased energy recovery (by 3786 MJ) and a reduced dependence on fossil fuels (Wang et al., 2024a). These observations are in line with those of previous studies indicating that a higher volatile solids content and anaerobic digestion efficiency contribute to an increase in energy recovery and, consequently, reduced carbon emissions (Wang et al., 2024a).

Additionally, shifting the electricity sources used for sludge management from fossil fuels (e.g., coal) to renewable sources (e.g., wind, solar, hydropower, and/or nuclear) could reduce carbon emissions (Fig. 6). In the sensitivity analysis, carbon emissions decreased by 1.09-fold in IS1 and 1.44-fold in SS2 when using renewable electricity sources compared with coal-based fossil fuels (Fig. 6). Moreover, the decision to use renewable energy sources resulted in comparable reductions in carbon emission in both IS1 and SS2. These observations are consistent with those of previous studies, suggesting that clean renewable energy sources typically emit fewer pollutants than traditional fossil fuels (Rahman and Alam, 2021; Staples et al., 2017). Notably, anaerobic digestion (in IS1) and incineration (in SS2), which have high energy demands, dominated carbon emission during sludge management (Di Capua et al., 2020) (Fig. S15c and e).

## Discussion

Selecting the appropriate scenario for sludge management is paramount. In this study, scenarios involving anaerobic digestion with energy recovery (Brazil and India), incineration with energy recovery (Russia and South Africa), or a combination of these steps (China) were found to most efficiently minimize environmental impacts while maximizing energy production. Conversely, sludge management scenarios that lack a stabilization process tend to have a substantial adverse environmental impact and may lead to a high energy demand if the treatment process involves energy-intensive methods (e.g., thermal drying). Compared with the worst scenarios, the best sludge management scenarios were found to enhance energy recovery by 1.4–98.4 times and to reduce the environmental impact by 1.5–21.4 times in BRICS countries. To further support the transition towards these optimal scenarios, it is essential to focus on advancing technology and developing effective policies (Chrispim et al., 2021; Zhang et al., 2017). Continued innovation in sludge management technologies is crucial, to enhance anaerobic digestion and incineration processes, improving their efficiency and reducing their environmental footprint (Chrispim et al., 2021). Concurrently, effective policy frameworks and investments are vital to facilitate the innovation and adoption of these advanced technologies. Governments should implement incentives for adopting state-of-the-art sludge treatment methods and provide subsidies for upgrading existing facilities. Relevant regulations are highly recommended to promote the use of renewable energy sources in sludge management processes and establish stringent environmental standards to reduce pollution (Tyagi and Lo, 2013; Wen et al., 2023). Additionally, policies that encourage collaboration among industry, academia, and government agencies can accelerate the development and deployment of innovative technologies (Li et al., 2023).

Enhanced energy production can provide a renewable alternative to fossil fuels and thus help to reduce carbon emissions. In the BRICS countries, shifting to the best sludge management scenarios from the worst is projected to reduce carbon emissions by 1.54–21.42 times by 2023 and by 2.15–24.92 times by 2050. Such a shift is also projected to reduce carbon emissions by 7.8 %–55.8 % in 2023 and 10.6 %–69.5 % in 2050 in the corresponding transport sector of each BRICS country (Supplementary Note 6). These projections highlight the importance of selecting the optimal sludge management scenario. Additionally, the replacement of fossil-based fuels with renewable energy sources for electricity production in the best sludge management scenarios is projected to further reduce carbon emissions by 23.5 %, 26 %, 69.3 %, 39.6 %, and 96.6 % compared with the worst scenarios in Brazil, Russia, India, China, and South Africa, respectively, in 2050 (Fig. 6). These findings highlight the significance of transitioning towards the use of renewable energy sources to achieve carbon neutrality goals. Given the global nature of environmental challenges, international cooperation is essential for sharing best practices and technological advancements. Collaborative research and knowledge exchange among BRICS countries can accelerate the adoption of effective sludge management strategies and drive innovation. Additionally, raising public awareness through educational programs for stakeholders, policymakers, industry professionals, and the general public is crucial (Li et al., 2023). Such initiatives can promote the adoption of best practices and support informed decision-making, leading to more effective and widespread implementation of advanced sludge management strategies.

Further improvements in energy recovery and reductions in carbon emissions can be achieved by adjusting the anaerobic digestion efficiency and volatile solids content during sludge management. Increasing the anaerobic digestion efficiency from 40 % to 70 % was shown to enhance energy production by 1.4–1.9 times and to reduce carbon emissions by 1.03–1.18 times. At the laboratory scale, various methods have been proposed to enhance anaerobic digestion efficiency, such as pre-treatment (e.g., free ammonia, advanced oxidation process, thermal, CaO_2_, etc.), the use of additives (e.g., lignosulfonate, zero-valent iron, etc.), and co-digestion with diverse substrates (e.g., microalgae, food waste, sugarcane bagasse, agricultural residues, etc.) (Liu et al., 2024; Wang et al., 2023a, 2023b; Wei et al., 2017). Despite these advances, the application of these methods in real-world sludge management scenarios (e.g., pilot-scale and full-scale studies) is still lacking, and additional research will be needed to realize actual improvements in energy production and carbon emission reduction. Additionally, it is also essential to explore novel approaches that enhance anaerobic digestion efficiency, while being cost-effective and environmentally friendly. Increasing the volatile solids content from 50 % to 80 % was also shown to enhance energy production by 1.45–2.35 times in South Africa and 1.26–1.76 times in India, while also reducing carbon emissions by 1.84–3.62 times and 1.04–1.11, respectively. Potential strategies for prioritizing higher volatile solids content include: 1) establishing separate sewer systems and minimizing leakage (especially in China) (Yang et al., 2015); 2) optimizing organic matter removal efficiency and adjusting sludge recirculation ratios during wastewater treatment (Nges and Liu, 2010; Shi et al., 2021); and 3) ensuring a balanced biodegradable ratio in mixed sludge, as primary clarifier sludge typically contains larger amounts of biodegradable matter than secondary clarifier sludge (Wang et al., 2024b). In the future, the benefits of these proposed technological advancements will warrant further investigation once they have been widely adopted in real-world scenarios.

## Conclusions

This study has provided a comprehensive assessment of sludge management scenarios used in the BRICS countries regarding their environmental impacts and energy balance. Across all the BRICS countries, sludge stabilization technologies involving energy recovery processes such as anaerobic digestion and incineration, were identified as the optimal sludge management scenarios, with incineration proving to be more effective than anaerobic digestion in terms of environmental benefits and energy production. Under the best scenario for each country, energy recovery was enhanced by 1.4–98.4 times and the environmental negative impact was reduced by 1.5–21.4 times compared with the worst scenario. The selection of sludge management scenarios and renewable energy sources played a crucial role in achieving carbon neutrality goals. The differences in energy production and carbon emission reduction between the best and worst scenarios in the BRICS countries could fulfill the annual electricity demands of up to 59 million people and account for up to 69.5 % of carbon emissions in the corresponding transport sectors. Transitioning to renewable energy could further reduce carbon emissions by up to 96.6 % in the transport sector in 2050, highlighting the importance of optimal scenario selection and the use of renewable energy. Optimizing the volatile solids content and anaerobic digestion efficiency, were shown to further reduce the environmental footprint by up to 3.62 times and to enhance energy recovery by up to 2.35 times, respectively. In summary, the findings of this study support the advancement of sludge valorization efforts to promote energy recovery and help reduce carbon emissions related to sludge management.

## Material and methods

### BRICS countries

A targeted analysis of the BRICS countries, namely Brazil, Russia, India, China, and South Africa, was conducted, focusing on sludge management scenarios and their energy balance and potential environmental impacts. Fig. 1 illustrates the population size and annual biosolids production of each BRICS country. The combined population and biosolids production amounted to 3.28 billion people and 42 Mt/year, respectively, representing 40.7 % of the total population and 42 %–56 % of the total biosolids production worldwide, as estimated in 2023 (Apollo, 2022). Specifically, Brazil, Russia, India, China, and South Africa had respective populations of 216.4, 144.4, 1428.6, 1425.6, and 60.4 million residents, accounting for 2.69 %, 1.80 %, 17.76 %, 17.72 %, and 0.75 % of the global population, respectively. Moreover, these nations respectively produced up to 2.5, 4.5, 15, 18, and 2 Mt of biosolids annually (Apollo, 2022; Kovalev et al., 2022) (Fig. 1).

### Functional unit, system boundary and life cycle inventory

The treatment of 1 ton of dry sludge (DS) was set as the functional unit in this analysis of BRICS countries. All calculations regarding materials, energy consumption, energy recovery, and emissions were based on this unit. The system boundaries considered various aspects such as the materials input, energy consumption, road transport, energy recovery, avoided products produced (which refers to avoided environmental impacts due to specific actions taken to prevent the use and production of products such as fertilizers and electricity), and direct emissions of pollutants to air, soil, and water environments. Notably, the construction and installation of sludge management infrastructures were not considered in this study, as numerous studies have shown that their overall environmental impact may be negligible (Hong et al., 2009; Singh et al., 2020a; Xu et al., 2014). The specific system boundaries of the selected sludge management scenario in each BRICS country are outlined in Figs. S1–S5.

Three scenarios (BS1, BS2, BS3) used in Brazil for sludge treatment and disposal were considered in this work (Fig. S1). BS1 comprises UASB, dewatering, thermal drying, and landfill; BS2 comprises UASB, dewatering, lime stabilisation, and land application; and BS3 comprises UASB, anaerobic digestion, cogeneration unit, dewatering, and land application. The characteristics of sludge were sourced from the National Solid Waste Policy in Brazil and published articles (see Table S1 for details).

Three scenarios (RS1, RS2, RS3) used in Russia for sludge management were considered in this study (Fig. S2). RS1 includes thickening, anaerobic digestion, cogeneration unit, dewatering, and land application; RS2 follows thickening, dewatering, thermal drying, incineration, and landfill; and RS3 involves thickening, dewatering and landfill. The characteristics of sludge were sourced from the Ministry of National Resources and Environment of the Russian Federation and published articles (seeTable S1 for details).

Three sludge management scenarios (IS1, IS2, IS3) used in India were considered in this work (Fig. S3). IS1 incorporates thickening, anaerobic digestion, cogeneration unit, dewatering, and land application; IS2 involves thickening, dewatering, and landfill; and IS3 consists thickening, anaerobic digestion, biogas flaring, dewatering, and land application. The characteristics of sludge were sourced from the Ministry of Environment, Forest, and Climate Change of the Government of India and published articles (see Table S1 for details).

Three sludge treatment and disposal scenarios (CS1, CS2, CS3) used in China were considered in this study (Fig. S4). CS1 includes thickening, anaerobic digestion, dewatering, cogeneration unit, dewatering, thermal drying, incineration, and landfill; CS2 encompasses thickening, anaerobic digestion, cogeneration unit, dewatering, and land application; and CS3 involves thickening, dewatering, composting, and land application. The characteristics of sludge were sourced from the Ministry of Housing and Urban-Rural Development of the People's Republic of China and published articles (see Table S1 for details).

Three sludge treatment and disposal scenarios (SS1, SS2, SS3) used in South Africa were considered in this work (Fig. S5). SS1 incorporates thickening, anaerobic digestion, cogeneration unit, dewatering, and landfill; CS2 follows thickening, dewatering, thermal drying, incineration, and landfill; and SS3 consists thickening, dewatering, thermal drying and land application. The characteristics of sludge were sourced from the Guidelines for the Utilization and Disposal of Wastewater Sludge issued by the Department of the Water Affairs and Forestry, Republic of South Africa and published articles (see Table S1 for details).

Specific data on each sludge management process were sourced from published articles focusing on sludge-related topics in Brazil, Russia, India, China and South Africa; the respective details are shown in Supplementary Notes 1–5.

### Unit process and inventory analysis

The energy flows and environmental impact of each sub-system were calculated using the life cycle inventory model, which includes 1) the heat and power consumption and generation across system processes (e.g., dewatering, thermal drying, anaerobic digestion, cogeneration unit, and incineration) and 2) the materials used (e.g., flocculant). Comprehensive details concerning the equations, inventory, and constants employed in this analysis are provided in Supplementary Notes 1–5.

**Dewatering:** Various mechanical dewatering methods, including filter press, centrifugation, vacuum filtration, and belt press, have been used extensively in sludge treatment (Zhang et al., 2022). The assessment of energy consumption by mechanical dewatering methods was conducted using Eq. (1):

(1) $$E_{c}=E_{c}\_Coeff\times \left(\frac{1}{1-w_{A}}-\frac{1}{1-w_{B}}\right)$$

where *E_c_* represents the energy consumption due to mechanical dewatering (kWh); *Ec_Coeff* represents the energy consumption coefficient (0.632 kWh/t DS); and *w_A_* and *w_B_* represent the sludge moisture content (%) before and after dewatering, respectively.

**Thermal drying:** The energy demand of sludge heating and water evaporation in thermal drying was calculated using the formulas outlined in Eqs. (2)–(7):

(2) $$Ec\_st=Cs\times (T_{2}\_A-T_{2}\_B)\times M_{2}$$

(3) $$Ec\_wt=Cw\times (T_{2}\_A-T_{2}\_B)\times M\_eva\_w$$

(4) E_eva_w=Q_eva_w×M_eva_w

(5) E_w_drying=Ec_wt+E_eva_w

(6) $$TEc_{2}=Ec\_wt+E\_eva\_w$$

(7) $$EAc_{2}=TEc_{2}\times (1+Eff\_hl)$$

where *Ec_st* and *Ec_wt* represent the energy consumption (MJ) of heat sludge and water, respectively; *T_2__A* and *T_2__B* represent the sludge temperature (°C) before and after thermal drying, respectively; and *C*s and *C*w denote the heat capacity of sludge and water, respectively, which are assumed to be equal at 4.186 kJ/(kg °C) (Yin et al., 2018). *M*_2_ and *M_eva_w* represent the mass (t) of sludge entering the thermal process and the evaporated water mass during drying, respectively; *E_eva_w* and *Q_eva_w* represent the energy required (MJ) for water evaporation and the vaporization heat value (2260 kJ/kg), respectively; and *E_w_drying* represents the energy required for water drying (MJ). *TEc*_2_, *EAc*_2_, and *Eff_hl* represent the theoretical energy consumption (MJ), actual energy consumption (MJ), and heat loss efficiency (20 %) (Hao et al., 2019) during drying, respectively.

**Anaerobic digestion:** Anaerobic digesters require energy input for regular operation, and this was calculated using Eq. (8):

(8) $$Pc{\_}_{3}=C_{s}\times (M_{3}+M_{3}\_w)\times (T_{3}-T_{i})$$

where Pc__3_ represents the theoretical power consumption for anaerobic digestion (MJ); *Cs* represents the heat capacity of wet sludge (equal to that of water, 4.186 kJ/(*t* × °C)); and *M_3_* and *M_3__w* denote the mass of sludge (dry basis) entering the digester and the mass (t) of water within the sludge, respectively. *T*_3_ and *T_i_* represent the temperature (°C) of anaerobic digestion and of the initial sludge, respectively.

(9) $$Pc{{\_}_{3}}^{\prime }=\frac{Pc{\_}_{3}}{\alpha }$$

where *Pc_*_3_*’* represents the actual energy consumption during anaerobic digestion (MJ), and *α* represents the conversion coefficient between the actual and theoretical energy consumption required to maintain the anaerobic digestion performance (%). Noticeably, biogas is generated during anaerobic digestion, and its volume was calculated using Eq. (10):

(10) $$D_{COD}=1.42\times Eff\times M_{3}\times VS$$

where *D_COD_* represents the degraded chemical oxygen demand (*COD)* during anaerobic digestion (t); *Eff* represents the anaerobic digestion efficiency (%); *M*_3_ represents the mass of dry sludge entering the digester (t); and *VS* represents the volatile solids content contained in the sludge (%).

(11) $$V_{3\_Biogas}=0.35\times D_{COD}$$

where *V*_3_*_Biogas* signifies the biogas production (m^3^), and 0.35 is the coefficient relating biogas production to degraded *COD.*

**Cogeneration unit:** Heat and power production during the cogeneration unit process were evaluated using Eqs. (12)–(13):

(12) $$P\_el=V_{3}\_biogas\times LHV\_biogas\times Eff\_CHP\_el$$

(13) $$P\_heat=V_{3}\_biogas\times LHV\_biogas\times Eff\_CHP\_heat$$

where *P_el* represents the electricity production in the cogeneration unit (MJ); *V*_3_*_biogas* denotes the biogas volume entering the cogeneration unit (m^3^); *LHV_biogas* represents the lower heating value of biogas from sludge anaerobic digestion, (6.5 kWh/m^3^) (Mukawa et al., 2022); and *Eff_CHP_el* and *Eff_CHP_heat* respectively represent the conversion efficiency of electricity and heat production in the cogeneration unit, with corresponding values of 40 % and 50 % (Xu et al., 2022).

**Incineration:** Heat and electricity recovery in the incineration process were determined using Eqs. (14)–(19):

(14) $$HCV=2.5\times 10^{5}\times (100\times Per\_om-5)$$

(15) $$LCV=HCV-Q\_eva\_w\times M_{5}\_w$$

(16) $$CV\_sludge=\frac{LCV}{1-w_{5}}$$

(17) $$E\_DS=CV\_sludge\times M_{6}$$

(18) E_ele=Eff_ele×E_DS

(19) E_the=Eff_the×E_DS

where *HCV, LCV*, and *CV_sludge* represent the high, low, and calorific values of sludge, respectively (GJ/t dry sludge); *Per_om* represents the percentage of organic matter in the sludge (%); *Q_eva_w* denotes the potential vaporization heat value of sludge (2260 kJ/kg); and *M*_5_*_w* and *w*_5_ indicate the mass and sludge moisture content of sludge before entering incineration (t), respectively. *E_DS, E_ele*, and *E_the* indicate the energy contents in dry sludge, electricity, and thermal production during incineration (GJ), respectively, and *M*_6_ (t), *Eff_ele* (%), and *Eff_the* (%) represent the mass of dry sludge before entering incineration and the conversion coefficients of electricity and thermal energy, respectively.

The life cycle inventory results for the sludge management scenarios used in the BRICS countries are outlined in Supplementary Tables 2–6.

### Life cycle analysis for energy balance and environmental impacts

The environmental impact of each sludge management scenario was calculated using the ReCiPe 2016 Midpoint (H) V1.07 method in SimaPro 9.4.0.1 software. This method is a widely recognized indicator used in life cycle assessment analysis (Laurent and Espinosa, 2015; Sternberg and Bardow, 2015; Wang et al., 2024a; Zhang et al., 2023). The environmental impact categories were selected from among those that have been reported as being most strongly correlated with sludge management in previous studies (Cartes et al., 2018; Chen et al., 2022; Evangelisti et al., 2014b; Li et al., 2017; Mayer et al., 2021; Singh et al., 2020a; Wang et al., 2024a). Five impact categories were quantified: global warming, terrestrial acidification, freshwater eutrophication, human carcinogenic toxicity, and fossil resource scarcity. The applied equivalent factors were CO_2_ eq for global warming, SO_2_ eq for terrestrial acidification, P eq for freshwater eutrophication, 1,4-DCB eq for human carcinogenic toxicity, and oil eq for fossil resource scarcity. The total environmental impact was calculated as the sum of the normalized individual impact category scores. The net energy balance represents the summed total energy across all processes within each sludge management scenario.

### Sensitivity analysis of the main contributors

Studies have identified the volatile solids content, anaerobic digestion efficiency, and electricity sources as key parameters that significantly influence the results of sludge management evaluation (Shahbeig and Nosrati, 2020; Zhao et al., 2023; Zhou et al., 2022). Consequently, these parameters were included in a sensitivity analysis of the optimal sludge management scenarios in the BRICS countries. The volatile solids content was selected based on those observed (Table S1) in the BRICS countries, while the values of anaerobic digestion efficiency were sourced from the previous studies (Li et al., 2018). To assess their influence, the parameters were adjusted in 10 % intervals. The selection of electricity sources was guided by the information shown in Fig. S12, which depicts the actual electricity compositions in the BRICS countries.

### Announced Pledges Scenario for projected carbon emissions in 2050

The Announced Pledges Scenario is based on an assumption that governments will meet all of their announced climate-related commitments fully and in a timely manner, including their longer-term net-zero emissions targets, pledged nationally determined contributions, and commitments in related areas such as energy access. This scenario also considers pledges made by businesses and other stakeholders that contribute to goals set by governments. Carbon emissions in 2050 were projected by multiplying the projected annual sludge production in each BRICS country by the corresponding carbon emissions per functional unit (1 t DS). Notably, these projected emissions were determined for both the best and worst sludge management scenarios (derived from an analysis in this study), based on the projected electricity generation sources to be used in the BRICS countries in 2050 as outlined in the Announced Pledges Scenarios, and the calculations of environmental impact were performed using the ReCiPe method. The projected electricity generation sources for each BRICS country in 2050 were determined from the World Energy Outlook 2023 report by the International Energy Agency; detailed results are presented in Fig. S14. The projected annual sludge production for each BRICS country was calculated by multiplying the population quantity, using data provided by the Department of Economic and Social Affairs, United Nations (Fig. S15), by the per capita sludge production, using data from previous studies (Appels et al., 2008). The ultimate outcome is illustrated in Fig. S17. Additionally, the projected carbon emissions in 2023 were similar to those in 2050, except for the electricity generation sources, which were based on the current composition of electricity generation.

The number of people whose annual electricity consumption demand could be met by the energy recovered from sludge management was calculated by dividing the total energy recovered by the per capita average annual energy consumption. Moreover, the proportion of carbon emissions from each sludge management scenario used in the BRICS countries compared with the carbon emissions from the transport sector was calculated based on the ratio of the projected values in 2023 and 2050. Data on the per capita annual electricity consumption and carbon emissions from the transport sector were obtained from Our World in Data (https://ourworldindata.org/). Detailed results are provided in Figs. S18 and S19.

## CRediT authorship contribution statement

**Zhenyao Wang:** Writing – review & editing, Writing – original draft, Visualization, Methodology, Investigation, Formal analysis, Conceptualization. **Xuan Li:** Writing – review & editing, Visualization, Supervision, Methodology, Formal analysis. **Huan Liu:** Writing – review & editing. **Jinhua Mou:** Writing – review & editing, Software. **Stuart J. Khan:** Writing – review & editing. **Carol Sze Ki Lin:** Writing – review & editing, Software, Resources. **Qilin Wang:** Writing – review & editing, Validation, Supervision, Project administration, Funding acquisition.

## Declaration of competing interest

The authors declare that they have no known competing financial interests or personal relationships that could appear to influence the work reported in this paper.

## Data Availability

Data will be made available on request.

## Appendix Supplementary materials

Image, application 1

## References

[bib0001] Adibimanesh B., Polesek-Karczewska S., Bagherzadeh F., Szczuko P., Shafighfard T. (2023). Energy consumption optimization in wastewater treatment plants: machine learning for monitoring incineration of sewage sludge. Sustain. Energy Technol. Assess..

[bib0002] Afrane S., Ampah J.D., Yusuf A.A., Jinjuan Z., Yang P., Chen J.L., Mao G. (2024). Role of negative emission technologies in South Africa's pathway to net zero emissions by 2050. Energy Sustain. Develop..

[bib0003] Altieri V.G., De Sanctis M., Barca E., Di Iaconi C. (2023). SBBGR technology for reducing waste sludge production during plastic recycling process: assessment of potential increase in sludge hazardousness. Sci. Total Environ..

[bib0004] Angouria-Tsorochidou E., Seghetta M., Trémier A., Thomsen M. (2022). Life cycle assessment of digestate post-treatment and utilization. Sci. Total Environ..

[bib0005] Apollo S. (2022). A review of sludge production in South Africa municipal wastewater treatment plants, analysis of handling cost and potential minimization methods. Phys. Sci. Rev..

[bib0006] Appels L., Baeyens J., Degrève J., Dewil R. (2008). Principles and potential of the anaerobic digestion of waste-activated sludge. Prog. Energ. Combust..

[bib0007] Arias A., Feijoo G., Moreira M. (2021). Benchmarking environmental and economic indicators of sludge management alternatives aimed at enhanced energy efficiency and nutrient recovery. J. Environ. Manage..

[bib0008] Bagheri M., Bauer T., Burgman L.E., Wetterlund E. (2023). Fifty years of sewage sludge management research: mapping researchers' motivations and concerns. J. Environ. Manage..

[bib0009] Cartes J., Neumann P., Hospido A., Vidal G. (2018). Life cycle assessment of management alternatives for sludge from sewage treatment plants in Chile: does advanced anaerobic digestion improve environmental performance compared to current practices?. J. Mater. Cycles Waste Manage..

[bib0010] Chen R., Yuan S., Chen S., Ci H., Dai X., Wang X., Li C., Wang D., Dong B. (2022). Life-cycle assessment of two sewage sludge-to-energy systems based on different sewage sludge characteristics: energy balance and greenhouse gas-emission footprint analysis. J. Environ. Sci..

[bib0011] Chrispim M.C., Scholz M., Nolasco M.A. (2021). Biogas recovery for sustainable cities: a critical review of enhancement techniques and key local conditions for implementation. Sustain. Citi. Soc..

[bib0012] Dad K., Wahid A., Khan A.A., Anwar A., Ali M., Sarwar N., Ali S., Ahmad A., Ahmad M., Khan K.A. (2019). Nutritional status of different biosolids and their impact on various growth parameters of wheat (Triticum aestivum L.). Saud. J. Biol. Sci..

[bib0013] Das A., Saini V., Parikh K., Parikh J., Ghosh P., Tot M. (2023). Pathways to net zero emissions for the Indian power sector. Energy Strategy Rev.

[bib0014] Dere A.L., Stehouwer R.C., Aboukila E., McDonald K.E. (2012). Nutrient leaching and soil retention in mined land reclaimed with stabilized manure. J. Environ. Qual..

[bib0015] Di Capua F., Spasiano D., Giordano A., Adani F., Fratino U., Pirozzi F., Esposito G. (2020). High-solid anaerobic digestion of sewage sludge: challenges and opportunities. Appl. Energ..

[bib0016] Ding A., Zhang R., Ngo H.H., He X., Ma J., Nan J., Li G. (2021). Life cycle assessment of sewage sludge treatment and disposal based on nutrient and energy recovery: a review. Sci. Total Environ..

[bib0017] Dong B., Liu X., Dai L., Dai X. (2013). Changes of heavy metal speciation during high-solid anaerobic digestion of sewage sludge. Bioresour. Technol..

[bib0018] Du W., Lu J., Hu Y., Xiao J., Yang C., Wu J., Huang B., Cui S., Wang Y., Li W. (2023). Spatiotemporal pattern of greenhouse gas emissions in China's wastewater sector and pathways towards carbon neutrality. Nat. Water.

[bib0019] Evangelisti S., Lettieri P., Borello D., Clift R. (2014). Life cycle assessment of energy from waste via anaerobic digestion: a UK case study. Waste Manage.

[bib0020] Evangelisti S., Lettieri P., Borello D., Clift R. (2014). Life cycle assessment of energy from waste via anaerobic digestion: a UK case study. Waste Manage.

[bib0021] Fang C., Huang R., Dykstra C.M., Jiang R., Pavlostathis S.G., Tang Y. (2019). Energy and nutrient recovery from sewage sludge and manure via anaerobic digestion with hydrothermal pretreatment. Environ. Sci. Technol..

[bib0022] Fang M., Chang C.-L. (2023). The role of COP26 commitment and technological innovation in depletion of natural resources: evidence from BRICS countries. Resour. Policy.

[bib0023] Guo Y., Gong H., Shi W., Fang N., Tan Y., Zhou W., Huang J., Dai L., Dai X., Guo Y. (2023). Insights into multisource sludge distributed in the Yangtze River basin, China: characteristics, correlation, treatment and disposal. J. Environ. Sci..

[bib0024] Hao X., Chen Q., van Loosdrecht M.C., Li J., Jiang H. (2020). Sustainable disposal of excess sludge: incineration without anaerobic digestion. Water Res..

[bib0025] Hao X., Li J., van Loosdrecht M.C., Jiang H., Liu R. (2019). Energy recovery from wastewater: heat over organics. Water Res..

[bib0026] Hong J., Hong J., Otaki M., Jolliet O. (2009). Environmental and economic life cycle assessment for sewage sludge treatment processes in Japan. Waste Manage.

[bib0027] Horttanainen M., Deviatkin I., Havukainen J. (2017). Nitrogen release from mechanically dewatered sewage sludge during thermal drying and potential for recovery. J. Clean. Prod..

[bib0028] Kovalev A.A., Mikheeva E.R., Kovalev D.A., Katraeva I.V., Zueva S., Innocenzi V., Panchenko V., Zhuravleva E.A., Litti Y.V. (2022). Feasibility study of anaerobic codigestion of municipal organic waste in moderately pressurized digesters: a case for the Russian Federation. Appl. Sci..

[bib0029] Laurent A., Espinosa N. (2015). Environmental impacts of electricity generation at global, regional and national scales in 1980–2011: what can we learn for future energy planning?. Energy Environ. Sci..

[bib0030] Li H., Jin C., Zhang Z., O'Hara I., Mundree S. (2017). Environmental and economic life cycle assessment of energy recovery from sewage sludge through different anaerobic digestion pathways. Energy.

[bib0031] Li L., Hua Y., Zhao S., Yang D., Chen S., Song Q., Gao J., Dai X. (2023). Worldwide research progress and trend in sludge treatment and disposal: a bibliometric analysis. ACS EST Eng..

[bib0032] Li R., Zhang Z., Li Y., Teng W., Wang W., Yang T. (2015). Transformation of apatite phosphorus and non-apatite inorganic phosphorus during incineration of sewage sludge. Chemosphere.

[bib0033] Li X., Zhang S., Shi J., Luby S.P., Jiang G. (2021). Uncertainties in estimating SARS-CoV-2 prevalence by wastewater-based epidemiology. Chem. Eng. J..

[bib0034] Li Y., Luo W., Lu J., Zhang X., Li S., Wu Y., Li G. (2018). Effects of digestion time in anaerobic digestion on subsequent digestate composting. Bioresour. Technol..

[bib0035] Liew C.S., Yunus N.M., Chidi B.S., Lam M.K., Goh P.S., Mohamad M., Sin J.C., Lam S.M., Lim J.W., Lam S.S. (2022). A review on recent disposal of hazardous sewage sludge via anaerobic digestion and novel composting. J. Hazard. Mater..

[bib0036] Liu B., Rajagopal D. (2019). Life-cycle energy and climate benefits of energy recovery from wastes and biomass residues in the United States. Nat. Energy.

[bib0037] Liu H., Li X., Zhang Z., Li J., Zhou T., Wang Z., Wang Q. (2024). Urine pretreatment enhances energy recovery by boosting medium-chain fatty acids production from waste activate sludge through anaerobic fermentation. Chem. Eng. J..

[bib0038] Liu H., Qiao H., Liu S., Wei G., Zhao H., Li K., Weng F. (2023). Energy, environment and economy assessment of sewage sludge incineration technologies in China. Energy.

[bib0039] Mayer F., Bhandari R., Gäth S.A. (2021). Life cycle assessment of prospective sewage sludge treatment paths in Germany. J. Environ. Manage..

[bib0040] Medina-Martos E., Istrate I.-R., Villamil J.A., Gálvez-Martos J.-L., Dufour J., Mohedano Á.F. (2020). Techno-economic and life cycle assessment of an integrated hydrothermal carbonization system for sewage sludge. J. Clean. Prod..

[bib0041] Mills N., Pearce P., Farrow J., Thorpe R., Kirkby N. (2014). Environmental & economic life cycle assessment of current & future sewage sludge to energy technologies. Waste Manage..

[bib0042] Morsink-Georgali P.-Z., Kylili A., Fokaides P.A., Papadopoulos A.M. (2022). Compost versus biogas treatment of sewage sludge dilemma assessment using life cycle analysis. J. Clean. Prod..

[bib0043] Mukawa J., Pająk T., Rzepecki T., Banaś M. (2022). Energy potential of biogas from sewage sludge after thermal hydrolysis and digestion. Energies.

[bib0044] Nges I.A., Liu J. (2010). Effects of solid retention time on anaerobic digestion of dewatered-sewage sludge in mesophilic and thermophilic conditions. Renew. Energ..

[bib0045] Nguyen D.H., Khominich I.P. (2023). The measurement of green economic quality in the BRICS countries: should they prioritize financing for environmental protection, economic growth, or social goals?. Russ. J. Econ..

[bib0046] Pasciucco F., Francini G., Pecorini I., Baccioli A., Lombardi L., Ferrari L. (2023). Valorization of biogas from the anaerobic co-treatment of sewage sludge and organic waste: life cycle assessment and life cycle costing of different recovery strategies. J. Clean. Prod..

[bib0047] Rahman M.M., Alam K. (2021). Clean energy, population density, urbanization and environmental pollution nexus: evidence from Bangladesh. Renew. Energ..

[bib0048] Sauve G., Van Acker K. (2020). The environmental impacts of municipal solid waste landfills in Europe: a life cycle assessment of proper reference cases to support decision making. J. Environ. Manage..

[bib0049] Shahbeig H., Nosrati M. (2020). Pyrolysis of municipal sewage sludge for bioenergy production: thermo-kinetic studies, evolved gas analysis, and techno-socio-economic assessment. Renew. Sust. Energ. Rev..

[bib0050] Shi W., Zhuang W.-E., Hur J., Yang L. (2021). Monitoring dissolved organic matter in wastewater and drinking water treatments using spectroscopic analysis and ultra-high resolution mass spectrometry. Water Res..

[bib0051] Singh A.D., Upadhyay A., Shrivastava S., Vivekanand V. (2020). Life-cycle assessment of sewage sludge-based large-scale biogas plant. Bioresour. Technol..

[bib0052] Singh V., Phuleria H.C., Chandel M.K. (2020). Estimation of energy recovery potential of sewage sludge in India: waste to watt approach. J. Clean. Prod..

[bib0053] Soterroni A.C., Império M., Scarabello M.C., Seddon N., Obersteiner M., Rochedo P.R., Schaeffer R., Andrade P.R., Ramos F.M., Azevedo T.R. (2023). Nature-based solutions are critical for putting Brazil on track towards net-zero emissions by 2050. Glob. Chang. Biol..

[bib0054] Staples M.D., Malina R., Barrett S.R. (2017). The limits of bioenergy for mitigating global life-cycle greenhouse gas emissions from fossil fuels. Nat. Energy.

[bib0055] Sternberg A., Bardow A. (2015). Power-to-What? – Environmental assessment of energy storage systems. Energy Environ. Sci..

[bib0056] Tang Y., Sun J., Dong B., Dai X. (2022). Thermal hydrolysis pretreatment-anaerobic digestion promotes plant-growth biostimulants production from sewage sludge by upregulating aromatic amino acids transformation and quinones supply. Environ. Sci. Technol..

[bib0057] Teoh S.K., Li L.Y. (2020). Feasibility of alternative sewage sludge treatment methods from a lifecycle assessment (LCA) perspective. J. Clean. Prod..

[bib0058] Tyagi V.K., Lo S.-L. (2013). Sludge: a waste or renewable source for energy and resources recovery?. Renew. Sust. Energ. Rev..

[bib0059] Usman K., Khan S., Ghulam S., Khan M.U., Khan N., Khan M.A., Khalil S.K. (2012). Sewage sludge: an important biological resource for sustainable agriculture and its environmental implications. Am. J. Bot..

[bib0060] Wang Y., Wei W., Wu S.-L., Ni B.-J. (2020). Zerovalent iron effectively enhances medium-chain fatty acids production from waste activated sludge through improving sludge biodegradability and electron transfer efficiency. Environ. Sci. Technol..

[bib0061] Wang Z., Li X., Liu H., Li J., Vodnar D.C., Lin C.S.K., Wang Q. (2024). Life cycle assessment of traditional and innovative sludge management scenarios in Australia: focusing on environmental impacts, energy balance, and economic benefits. Resour. Conserv. Recy..

[bib0062] Wang Z., Li X., Liu H., Zhou T., Li J., Li Y., Lin C.S.K., Wang Q. (2024). Triclosan in sludge: exploring its journey from the sewage treatment plants to land application and potential impacts on the environment. Crit. Rev. Environ. Sci. Technol..

[bib0063] Wang Z., Li X., Liu H., Zhou T., Li J., Siddiqui M.A., Lin C.S.K., Hatshan M.R., Huang S., Cairney J.M. (2023). Enhancing methane production from anaerobic digestion of secondary sludge through lignosulfonate addition: feasibility, mechanisms, and implications. Bioresour. Technol..

[bib0064] Wang Z., Li X., Liu H., Zhou T., Qin Z., Mou J., Sun J., Huang S., Chaves A.V., Gao L. (2023). Bioproduction and applications of short-chain fatty acids from secondary sludge anaerobic fermentation: a critical review. Renew. Sust. Energ. Rev..

[bib0065] Wang Z., Li X., Siddiqui M.A., Liu H., Zhou T., Zheng L., Huang S., Gao L., Lin C.S.K., Wang Q. (2023). Effect of humic substances on the anaerobic digestion of secondary sludge in wastewater treatment plants: a review. Environ. Chem. Lett..

[bib0066] Wei W., Zhou X., Wang D., Sun J., Wang Q. (2017). Free ammonia pre-treatment of secondary sludge significantly increases anaerobic methane production. Water Res..

[bib0067] Weihs G.F., Jones J., Ho M., Malik R., Abbas A., Meka W., Fennell P., Wiley D. (2022). Life cycle assessment of co-firing coal and wood waste for bio-energy with carbon capture and storage–New South Wales study. Energ. Convers. Manage..

[bib0068] Wen L., Yang F., Li X., Liu S., Lin Y., Hu E., Gao L., Li M. (2023). Composition of dissolved organic matter (DOM) in wastewater treatment plants influent affects the efficiency of carbon and nitrogen removal. Sci. Total Environ..

[bib0069] Xu C., Chen W., Hong J. (2014). Life-cycle environmental and economic assessment of sewage sludge treatment in China. J. Clean. Prod..

[bib0070] Xu Y., Liu R., Liu H., Geng H., Dai X. (2022). Novel anaerobic digestion of waste activated sludge via isoelectric-point pretreatment: ultra-short solids retention time and high methane yield. Water Res..

[bib0071] Yang G., Zhang G., Wang H. (2015). Current state of sludge production, management, treatment and disposal in China. Water Res..

[bib0072] Yang H., Guo Y., Fang N., Dong B. (2023). Life cycle assessment of sludge anaerobic digestion combined with land application treatment route: greenhouse gas emission and reduction potential. J. Environ. Chem. Eng..

[bib0073] Yin Z., Hoffmann M., Jiang S. (2018). Sludge disinfection using electrical thermal treatment: the role of ohmic heating. Sci. Total Environ..

[bib0074] Yoshida H., ten Hoeve M., Christensen T.H., Bruun S., Jensen L.S., Scheutz C. (2018). Life cycle assessment of sewage sludge management options including long-term impacts after land application. J. Clean. Prod..

[bib0075] Zhang Q., Hu J., Lee D.-J., Chang Y., Lee Y.-J. (2017). Sludge treatment: current research trends. Bioresour. Technol..

[bib0076] Zhang S., Wang F., Mei Z., Lv L., Chi Y. (2021). Status and development of sludge incineration in China. Waste Biomass Valori..

[bib0077] Zhang X., Schwarze M., Schomäcker R., van de Krol R., Abdi F.F. (2023). Life cycle net energy assessment of sustainable H2 production and hydrogenation of chemicals in a coupled photoelectrochemical device. Nat. Commun..

[bib0078] Zhang X., Ye P., Wu Y. (2022). Enhanced technology for sewage sludge advanced dewatering from an engineering practice perspective: a review. J. Environ. Manage..

[bib0079] Zhao S., Yan K., Wang Z., Gao Y., Li K., Peng J. (2023). Does anaerobic digestion improve environmental and economic benefits of sludge incineration in China? Insight from life-cycle perspective. Resour. Conserv. Recyc..

[bib0080] Zheng G.-D., Gao D., Chen T.-B., Luo W. (2007). Stabilization of nickel and chromium in sewage sludge during aerobic composting. J. Hazard. Mater..

[bib0081] Zhou T., Li X., Liu H., Dong S., Zhang Z., Wang Z., Li J., Nghiem L.D., Khan S.J., Wang Q. (2024). Occurrence, fate, and remediation for per-and polyfluoroalkyl substances (PFAS) in sewage sludge: a comprehensive review. J. Hazard. Mater..

[bib0082] Zhou X., Li J., Zhao X., Yang J., Sun H., Yang S.-S., Bai S. (2022). Resource recovery in life cycle assessment of sludge treatment: contribution, sensitivity, and uncertainty. Sci. Total Environ..

