# Supplementary material for: Evaluating energy balance and environmental footprint of sludge management in BRICS countries
Source: Water Res X. 2024 Sep 1;25:100255. doi: 10.1016/j.wroa.2024.100255 (PMC11404187; doi:10.1016/j.wroa.2024.100255)
Supplement: Supplementary file 1 [file mmc1.docx]

**Supplementary materials**

**Evaluating energy balance and environmental footprint of sludge management in BRICS countries**

Zhenyao Wang ^a^, Xuan Li ^a,^*, Huan Liu ^a^, Jinhua Mou ^b^, Stuart J. Khan ^c^, Carol Sze Ki Lin ^b^, Qilin Wang ^a,*^

^a^ Center for Technology in Water and Wastewater, School of Civil and Environmental Engineering, University of Technology, Ultimo, NSW, 2007, Australia

^b^ School of Energy and Environment, City University of Hong Kong, Tat Chee Avenue, Kowloon, Hong Kong, China

^c^ School of Civil Engineering, University of Sydney, NSW 2006, Australia

* Corresponding authors:

Prof. Qilin Wang; Email address: [Qilin.Wang@uts.edu.au](mailto:Qilin.Wang@uts.edu.au)

Dr. Xuan Li; Email address: Xuan.Li@uts.edu.au

# Supplementary Notes

## Supplementary Note 1: Sources of data on sludge treatment and disposal in Brazil

In Brazil, wastewater treatment plants have widely adopted up-flow anaerobic sludge blanket technology to treat wastewater effectively. The following list describes the specific sources of data on each sludge treatment and disposal process.

**Dewatering:** The up-flow anaerobic sludge blanket technology yields sewage sludge with a moisture content of 93.5% (Medeiros et al., 2023), which serves as the input sludge for subsequent treatment processes. The electricity consumption during dewatering was calculated based on Eq. 1 (Unit process and inventory analysis section). At the local sewage treatment plant in Brazil, 2 kg of polypropylene/t dry sludge (DS) was added as the flocculant (Avancini et al., 2021; Guimarães and Urashima, 2013). After dewatering, the sludge had a moisture content of 77% (Avancini et al., 2021).

**Thermal drying:** The energy requirements for heating the sludge and evaporating water during thermal drying were calculated as detailed in the Unit process and inventory analysis section. The initial temperature of the sludge before thermal drying was assumed to be the annual average temperature of Brazil, which is approximately 26 ℃. The specific thermal drying temperature was set at 100 ℃ in accordance with the technology standards for sludge treatment in municipal sewage treatment plants, with an estimated heat loss efficiency of 20% during the drying process (Hao et al., 2019). Following this process, the moisture content of the sludge was reduced to 60% (Cañote et al., 2021).

**Landfill:** The transport distance from the sewage treatment plant to the landfill site was approximately 27 km (do Amaral et al., 2018). During the landfill process, pertinent emissions were released into the air; these were generated primarily from the combustion of landfill biogas through flares (Cañote et al., 2021) and water discharge after the sludge had been deposited in the landfill (Mayer et al., 2021). Notably, the specific methane production was assumed to be 4.6 Nm^3^ CH_4_/t volatile solids according to data from the landfill site (Cañote et al., 2021). These emissions were accompanied by various emission factors, namely NO_x_, CO, SO_2_, HCl, HF, and particulate matter (PM) (Cañote et al., 2021). Further elaboration on these factors can be found in Table S2.

**Lime stabilization:** Lime was transported from plants to the sewage treatment facility over a distance of approximately 102 km (do Amaral et al., 2018). To achieve sludge stabilization, lime was applied at a dosage of 0.3 t/t DS, a technique rooted in the local sewage treatment plant’s practical experience (do Amaral et al., 2018). During lime stabilization, exhaust gases, including NH_3_, CO_2_, CO, NO_x_, and PM, were emitted (do Amaral et al., 2018). Such emissions are inherent to the process and might contribute to the overall environmental impact of sludge management.

**Land application:** The distance from the sewage treatment plant to the designated land site was approximately 187 km (do Amaral et al., 2018). The land application of sludge is regulated by the metal ion concentration limitations outlined in Conama 375/06 Resolution, Brazil (Bittencourt, 2018). Sludge contains valuable components such as nitrogen and phosphorus, which can act as a natural fertilizer by effectively enriching the soil with essential nutrients. Specifically, the sludge contained 10.2 g/kg of nitrogen and 3.6 g/kg of phosphorus, which would contribute substantially to soil fertility (do Amaral et al., 2018). However, it is essential to acknowledge that the land application of sludge also entails the release of pollutants into the surrounding environment (i.e., air, soil, and water) (do Amaral et al., 2018); details of the specific emission factors are provided in Table S2.

**Anaerobic digestion:** The energy and electricity required to maintain anaerobic digestion performance were calculated based on the equation expounded in Unit process and inventory analysis section. During anaerobic digestion, the organic matter within sludge underwent degradation (Wang et al., 2023), with an anaerobic digestion efficiency of 40% (Zhao et al., 2023). This degradation yielded biogas, a valuable product (Wang et al., 2023). The conversion coefficient between the degraded chemical oxygen demand (COD; kg) and CH_4_ (m^3^) was 0.35, determined from the practical experience of the sewage treatment plants (Tauber et al., 2019). Additionally, the resulting biogas from this process comprised 65% CH_4_, 30% CO_2_, 1.5% N_2_, 1.5% H_2_, and 0.05% H_2_S (Xu et al., 2014). Notably, a minor biogas leakage of approximately 3% occurred during anaerobic digestion (Medina-Martos et al., 2020).

**Cogeneration unit:** Cogeneration unit technology is commonly employed to efficiently convert biogas into heat and electricity, with total transformation efficiency rates as high as 90% (Xu et al., 2022). In an example of its remarkable efficiency, a notable 40% of the biogas was converted into electricity, while 50% was transformed into valuable heat energy, thus reducing the operational costs of sewage treatment plants (Xu et al., 2022). During operation, a cogeneration unit system was reported to consume lubricating oil at a rate of 2.35 × 10^-4^ kg/kWh per unit of electricity production (Mayer et al., 2021). During combustion within the cogeneration unit, biogas had a low heating value of approximately 6.5 kWh/m^3^ (Mukawa et al., 2022). Importantly, however, along with energy production, the cogeneration unit process also entails the emission of exhaust gases such as CO_2_, CH_4_, CO, SO_2_, NO, NO_x_, N_2_O, and PM (Alengebawy et al., 2022); the specific emission factors of these exhaust gases are shown in Table S2.

## Supplementary Note 2: Sources of data on sludge treatment and disposal in Russia

**Thickening:** Initially, the sludge had a moisture content of 99% prior to thickening (Kiselev et al., 2019), which was subsequently reduced to 95% (Kovalev et al., 2022). To enhance the efficiency of flocculation and optimize solid–liquid separation, polyacrylamide was selected as the flocculant at a dosage of 4 kg/t DS (Kalyuzhnyi, 2007). Drawing from practical application, the thickening process was estimated to consume 14 kWh electricity/t DS (Zhao et al., 2023).

**Anaerobic digestion:** The volatile solids content of the input sludge for anaerobic digestion was 65% (Kovalev et al., 2022). The initial sludge temperature (4.9 ℃) was consistent with the average annual temperature in Russia. However, the efficiency of anaerobic digestion of the sludge, approximately 35% (Kalyuzhnyi, 2007), was lower than that observed in Brazil (40%). The remaining parameters remained consistent with those measured in Brazil.

**Cogeneration unit:** The pertinent parameters remained unchanged and were consistent with the values utilised in the scenario in Brazil.

**Dewatering:** The calculated electricity consumption for dewatering was consistent with those of scenarios in Brazil, as detailed in the Unit process and inventory analysis section. The chosen flocculant, polyacrylamide, was administered at a rate of 4 kg/t DS. Notably, although the initial moisture content of sludge differed across the scenarios (96.15% in RS1, 95% in RS2 and RS3), a post-dewatering uniform moisture content of 60% was achieved (Kashkovsky et al., 2014).

**Land application:** The distance between the sewage treatment plant and the land application site was approximately 110 km (Ravi et al., 2022). The regulatory limits for metal ion concentrations in sludge used for agricultural purposes adhered to the guidelines set by the Russian Federation, as stipulated in RussianGost|Official Regulatory Library-GOST R 54534-2011 (LeBlanc et al., 2009). The sludge composition featured a potassium proportion (expressed as K_2_O) of 0.70% kg/kg DS, nitrogen content (expressed as N, calcium ammonium nitrate) of 1.50 kg/kg DS, and a phosphorus component (expressed as P_2_O_5_) of 4.50 kg/kg DS. The emission factors for pollutants introduced into the soil, water, and air due to land application are detailed in Table S3.

**Thermal drying:** The temperature of the sludge before entering the drying bed matched the average annual temperature of Russia (4.9 ℃). The required electricity and heat to reduce the sludge moisture content from 60% to 40% were calculated according to the methodology outlined in the Unit process and inventory analysis section.

**Incineration:** Sludge was required to have a moisture content of 40% before entering the incineration process (Värri et al., 2010). The high and low calorific values of sludge and the electricity consumption during the incineration process were determined using the approach detailed in Unit process and inventory analysis section. The cumulative conversion efficiency of sludge during incineration was approximately 84%; 42% was converted into electricity, and the remainder was transformed into heat (Zhao et al., 2021). Furthermore, within the context of incineration, NaOH, NaHCO_3_, lime, and active carbon additive were incorporated at rates of 15.41 kg/t organic matter, 48.43 kg/t organic matter, 94.50 kg/t organic matter, and 1.40 kg/t dry matter, respectively, to curtail the emission of pollutants (Zhao et al., 2021). Moreover, the exhaust gases from sludge incineration predominantly comprised CO, NO_x_, NH_3_, SO_2_, and PM (Zhao et al., 2021); the specific emission factors are detailed in Table S3.

**Landfill:** The landfill processes comprised one of two components: ash treatment within the landfill after sludge incineration (RS2), or the treatment of dewatered sludge landfill (RS3). The transportation distance from the Kuryanovskoy sewage treatment plant to the Aleksinsky landfill in the Moscow region was approximately 180 km. The emissions resulting from leachate treatment of the incinerated sludge (RS2) were obtained from a prior study (2021), and the specific data are detailed in Table S3.

## Supplementary Note 3: Sources of data on sludge treatment and disposal in India

**Thickening:** The initial sludge moisture content before thickening was 99% (Singh et al., 2020b), and this was reduced to 92% after thickening (Ghosh et al., 2020; Kumar et al., 2023). In India, the volatile solids content within sewage sludge was reported to be as high as 83% (Ghosh et al., 2020; Kumar et al., 2023). Additionally, the flocculant demand (including type and dosage) (Patel and Singh, 2022) and electricity consumption during thickening (Zhao et al., 2023) mirrored those of scenarios used in Brazil.

**Anaerobic digestion:** Before entering anaerobic digestion, the sludge temperature was aligned with India’s average annual temperature (26 ℃). This crucial information was sourced from the India Meteorological Department, a reliable climate information authority. Notably, the 5% rate of biogas leakage in India differed from the rates in the other BRICS countries (Patel and Singh, 2022). The remaining parameters associated with anaerobic digestion were consistent with those in Brazil.

**Cogeneration unit:** The pertinent parameters employed in the cogeneration unit process were consistent with those adopted in Brazil.

**Dewatering:** The electricity consumption throughout dewatering was assessed using the equation outlined in the Unit process and inventory analysis section. The selected flocculant remained consistent with that utilized in sewage treatment plants across Russia.

**Land application:** The transportation distance from sewage treatment plants to the agricultural application site was approximately 20 km, as documented in a previous study (Patel and Singh, 2022). The metal ion concentration in sewage sludge adhered to the guidelines prescribed by the government of India (Saha et al., 2015). Furthermore, the precise nitrogen, phosphorus, and potassium contents within the sewage sludge were found to align with the values reported in previous literature: 1.31% kg/kg DS, 2.46 kg/kg DS, and 1.83% kg/kg DS, respectively (Saha et al., 2017).

**Biogas flaring:** In India, biogas flaring systems are commonly used to effectively manage excess biogas (Tyagi et al., 2021). The composition of exhaust gases emanating from the biogas flaring system aligned with the findings of a recent study by Singh et al. (Singh et al., 2020a).

**Landfill:** The distance separating the Okhla sewage treatment plant from the Okhla landfill was approximately 8 km. Furthermore, the emissions to water and air resulting from sludge treatment through landfilling were consistent with the scenarios detailed in Table S4.

## Supplementary Note 4: Sources of data on sludge treatment and disposal in China

The following fundamental sources of data on the processes included in multiple scenarios for sludge treatment and disposal were used.

**Thickening:** The moisture content of the sludge was reduced from 99% (Huang et al., 2023; Zhao et al., 2023) to 95% (Yang et al., 2015) during thickening. The average volatile solids content of the sludge in sewage treatment plants was obtained from engineers working in Chinese sewage treatment facilities (i.e., private consultation). The requirements for flocculant utilization and electricity demand in this process aligned with the values used in Russia.

**Anaerobic digestion:** The sludge temperature was set to the average annual temperature in China (15 ℃). The specific data were obtained from the Blue Book on Climate Change of China, which was released by the China Meteorological Administration. Moreover, the temperature of the anaerobic digestion process adhered to the guidelines issued by the Ministry of Housing and Urban-Rural Development of the People’s Republic of China.

**Cogeneration unit, Dewatering, Thermal drying, and Incineration processes:** The calculations of parameters and the data sources mirrored those used in the scenarios implemented in Brazil.

**Landfill and Land application processes:** For sludge transportation, the distance from the sewage treatment plant to the landfill site was approximately 58 km (Chen et al., 2020; Li et al., 2013); the distance to the land application site was approximately 80 km (Diaz-Elsayed et al., 2020). Furthermore, sludge was used in agricultural applications according to a government document (2018) that stipulates limits on the permissible metal ion content for such usage. The emissions associated with sludge landfill treatment and those resulting from sludge agricultural application are shown in Table S5.

**Composting and subsequent Land application:** The electricity consumption and diesel demand during composting were derived from a preceding study (Tarpani and Azapagic, 2018). Similarly, information about the products avoided after composting the sludge mass and the exhaust gas emissions during composting was extracted from published papers (Mannarino et al., 2022; Tarpani and Azapagic, 2018; Zhuang, 2021). The transportation distance from the composting facility to the land site was approximately 20 km (Chai et al., 2015). The composition of the composting sludge (e.g., the carbon and nitrogen contents and heavy metal ion concentration) was informed by previous studies (Kulikowska and Gusiatin, 2015).

## Supplementary Note 5: Sources of data on sludge treatment and disposal in South Africa

The following sources of data on sludge stabilisation scenarios in South Africa were used:

**Thickening:** The moisture content of the influent sludge was 99% (Kiselev et al., 2019), which was reduced to 95% after the thickening process. The volatile solids content in sludge was 70% (Apollo, 2022).

**Anaerobic digestion:** The initial temperature of the sludge was equal to the average annual temperature of South Africa (17.5 ℃). The other parameters associated with anaerobic digestion were consistent with those used in the scenarios in Brazil.

**Cogeneration unit and Dewatering, Thermal drying, and Incineration:** The parameters associated with these processes were consistent with those used in Brazil.

**Land application:** The distance required to transport sludge from the sewage treatment plant to its destination for agricultural application was approximately 75 km (Tesfamariam et al., 2020). The nitrogen, phosphorus, and potassium contents in sludge were reported to be 3.0%, 2.0%, and 0.3%, respectively (Tesfamariam et al., 2020). The metal ion concentration in sludge complied with official government documents (Waal, 2008). Additionally, the relevant emission factors associated with the agricultural application of sludge are elaborated in Table S6.

**Landfill:** The distance between the northern sewage treatment plant and the landfill site at Clare Hills, Durban, was about 15 km. The other parameters were the same as those used in Brazil.

## Supplementary Note 6: Projections of carbon emissions associated with sludge management in BRICS in 2050, maintaining the same composition of energy sources as in 2023

In terms of environmental impact, the worst sludge management scenario, CS3 in China, is projected to contribute the largest amount of carbon emissions by 2050, reaching 27.39 Mt CO_2_ eq; this is projected to be followed by the worst scenarios in India (IS3: 3.35 Mt CO_2_ eq), Brazil (BS1: 2.07 Mt CO_2_ eq), Russia (RS3: 0.43 Mt CO_2_ eq) and South Africa (SS3: −0.16 Mt CO_2_ eq) (Fig. S20). Transitioning to the best scenario, CS1 in China and India, which is projected to achieve the greatest carbon emission savings of 15.03 Mt CO_2_ eq, followed by South Africa (SS2: 3.37 Mt CO_2_ eq), Brazil (BS3: 2.38 Mt CO_2_ eq), and Russia (RS2: 0.43 Mt CO_2_ eq) (Fig. S20). The disparity in carbon emissions between the best and worst scenarios is projected to account for 69.5%, 66.5%, 46.1%, 23.5%, and 10.6% of carbon emissions from the transport sectors in South Africa, India, China, Brazil, and Russia, respectively. Notably, the net energy balance of sludge management in BRICS countries in 2050 with different energy sources remains consistent (In one, the composition of energy sources remains consistent with those in 2023, while in another, there is a transformation towards renewable energy sources in line with the Announced Pledges Scenarios). Therefore, this aspect of the content is omitted in this section.

# Supplementary Figures


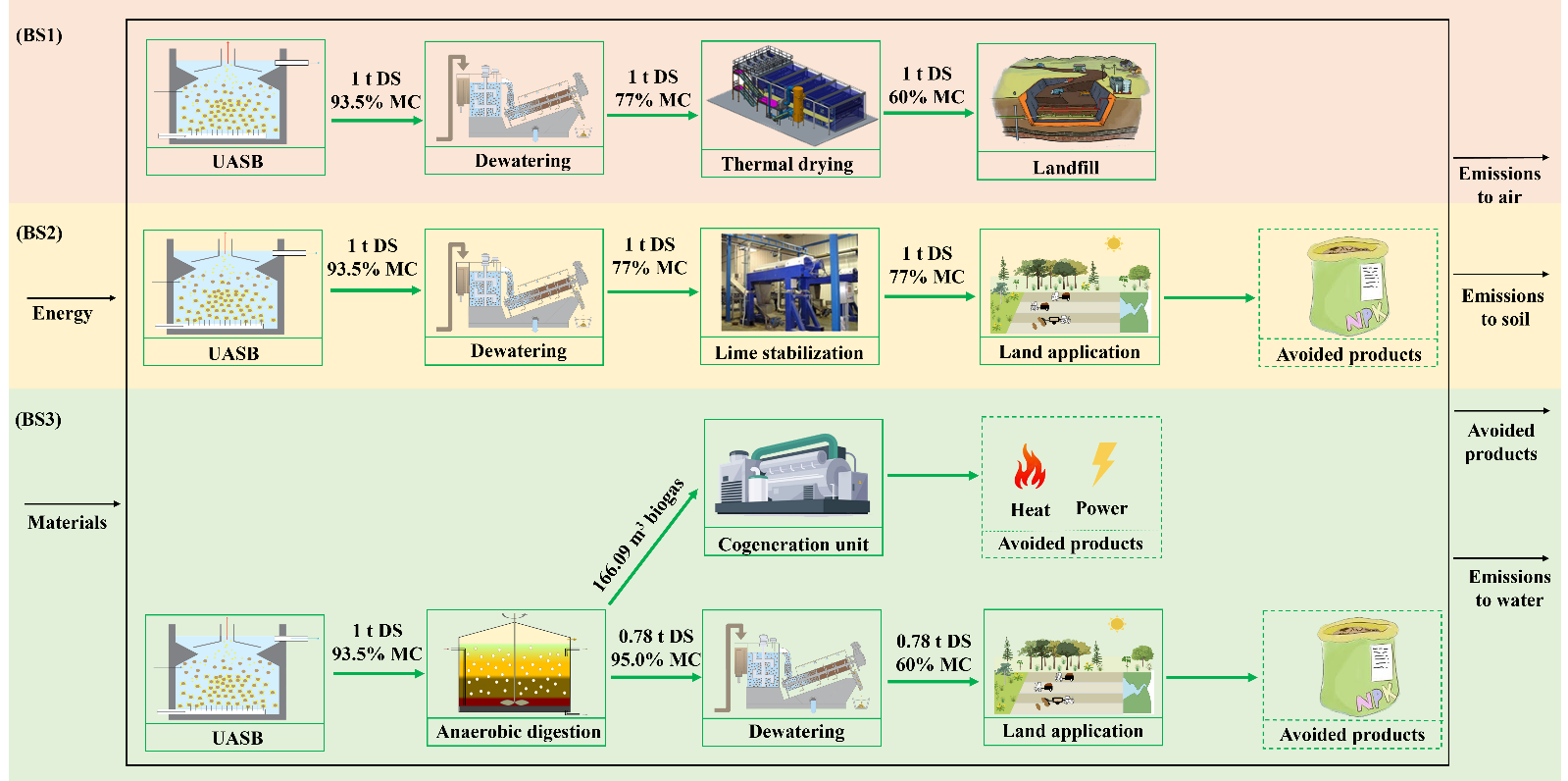


**Fig. S1.** System boundaries and mass flows of different sludge management scenarios used in Brazil. Note: BS1–3 represent the scenarios implemented in Brazil. UASB, DS, and MC represent the up-flow anaerobic sludge blanket, dry sludge, and sludge moisture content, respectively. Green dashed lines denote avoided products (energy or fertiliser).


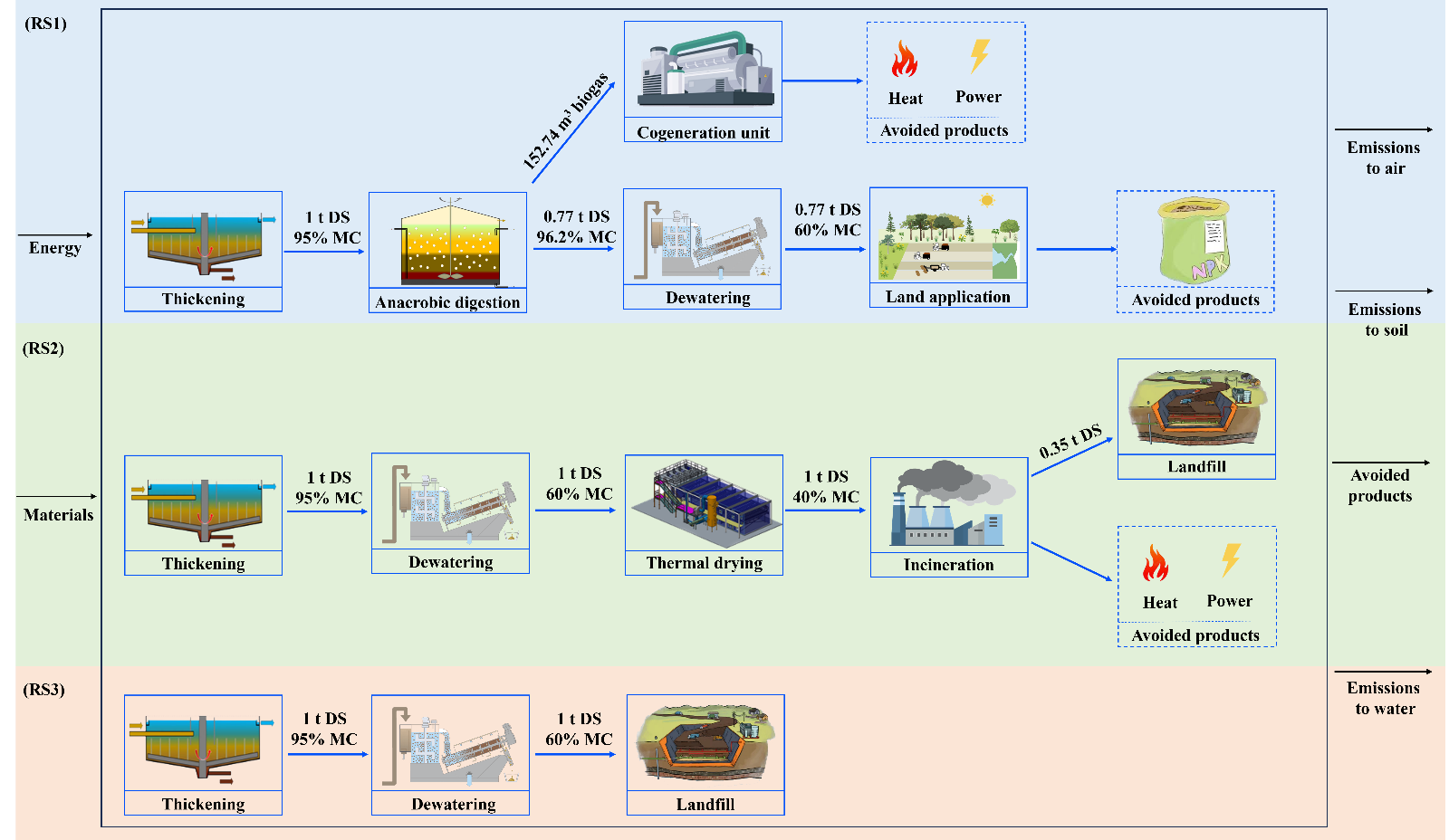


**Fig. S2.** System boundaries and mass flows of different sludge management scenarios used in Russia. Note: RS1–3 represent the scenarios implemented in Russia. DS and MC represent the dry sludge and sludge moisture content, respectively. Blue dashed lines indicate avoided products (energy or fertiliser).


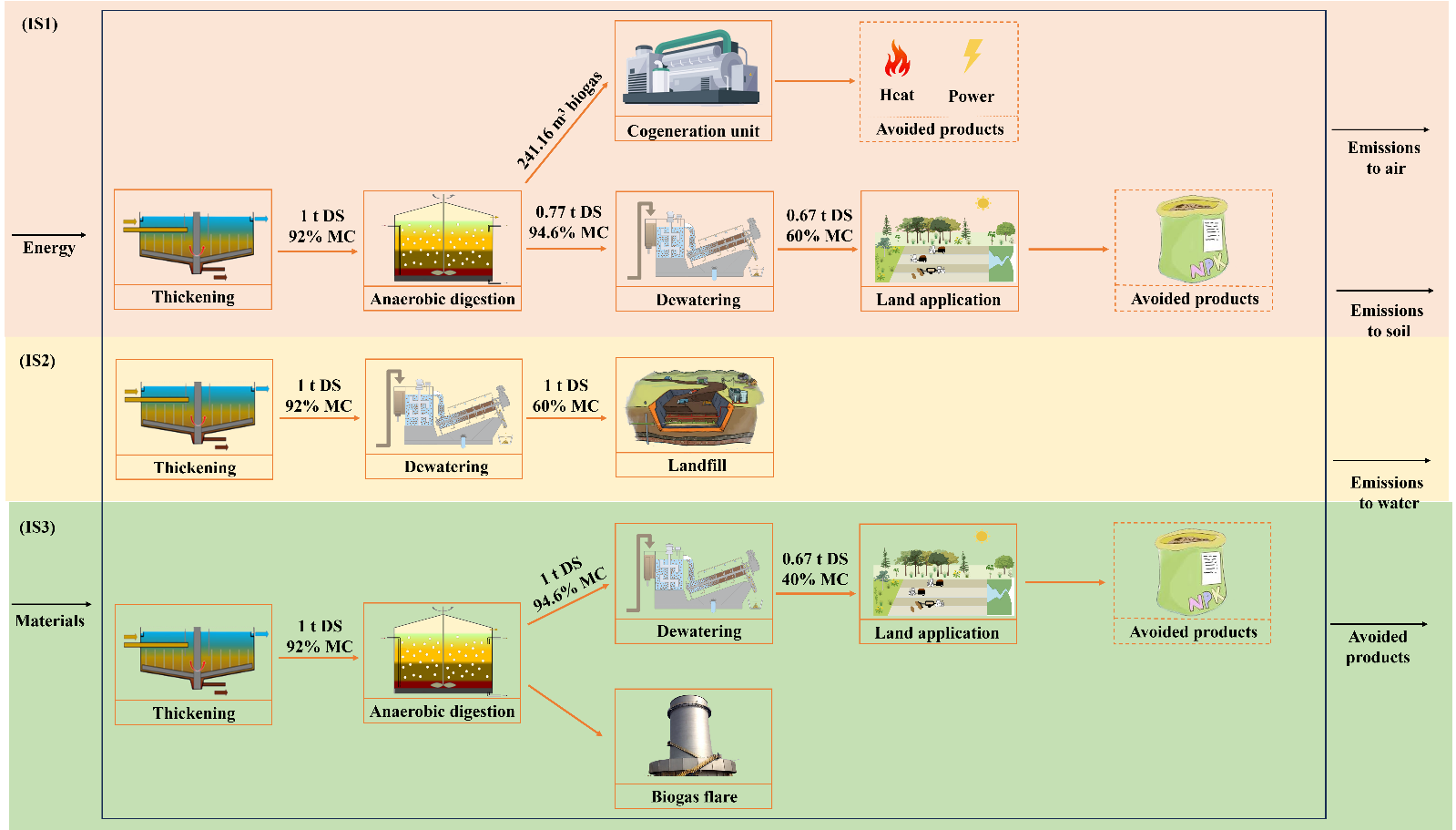


**Fig. S3.** System boundaries and mass flows of sludge management scenarios used in India. Note: IS1–3 represent the scenarios implemented in India. DS and MC represent the dry sludge and sludge moisture content, respectively. Orange dashed lines indicate avoided products (energy or fertiliser).


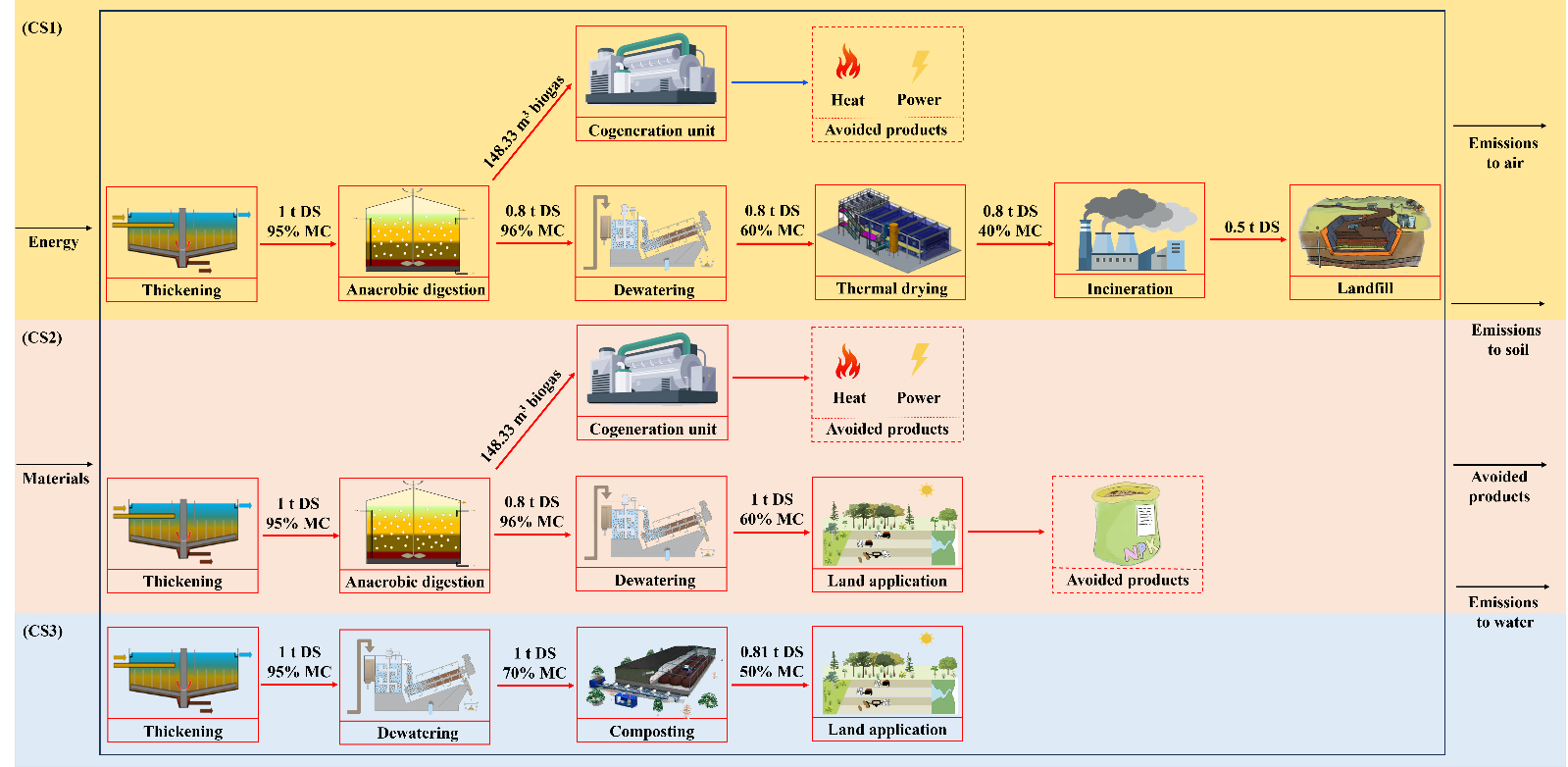


**Fig. S4.** System boundaries and mass flows of sludge management scenarios used in China. Note: CS1–3 represent the scenarios implemented in China. DS and MC represent the dry sludge and sludge moisture content, respectively. Red dashed lines indicate avoided products (energy or fertiliser).


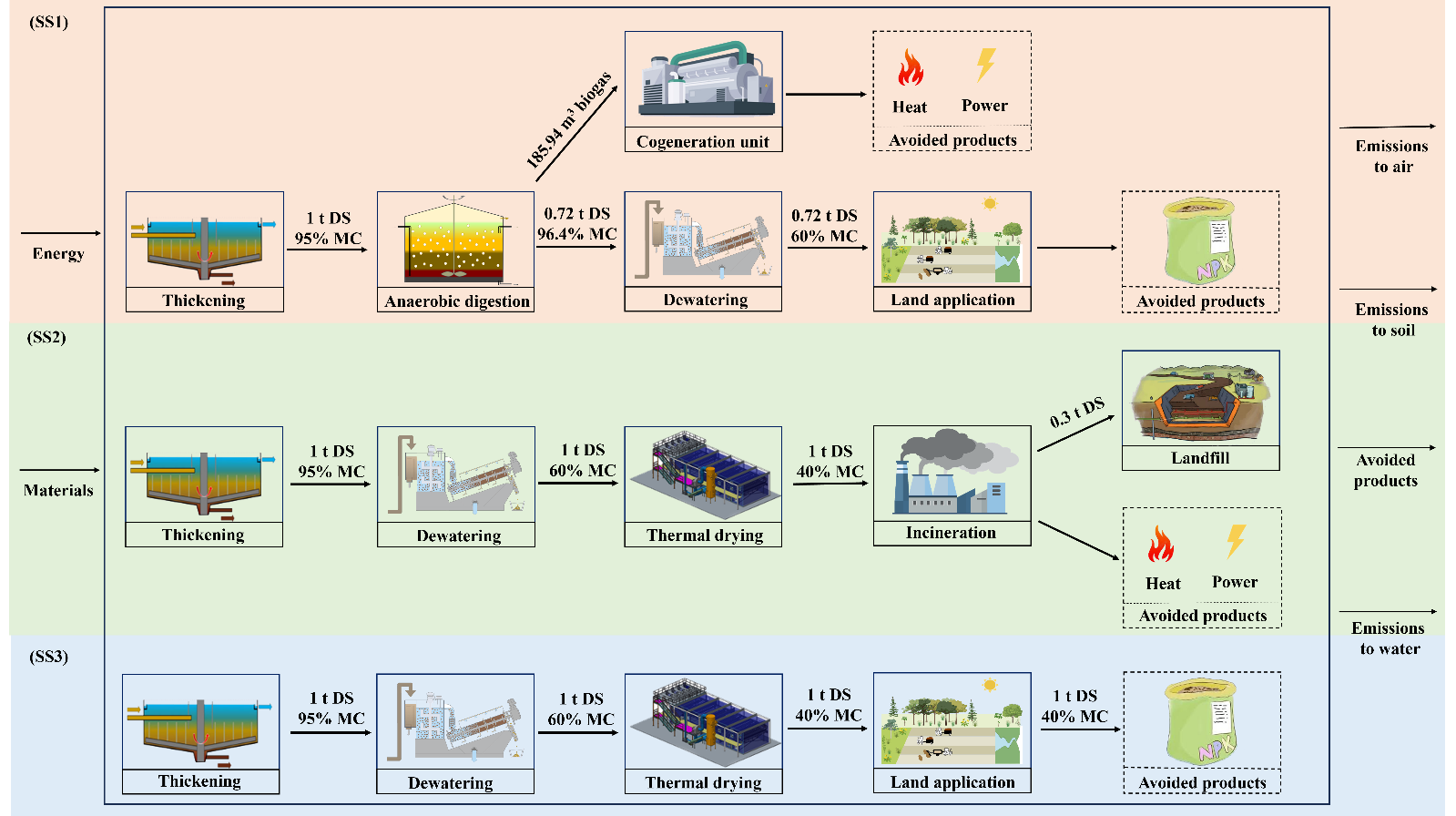


**Fig. S5.** System boundaries and mass flows of different sludge management scenarios used in South Africa. Note: SS1–3 represent the scenarios implemented in South Africa. DS and MC represent the dry sludge and sludge moisture content, respectively. Black dashed lines indicate avoided products (energy or fertiliser).


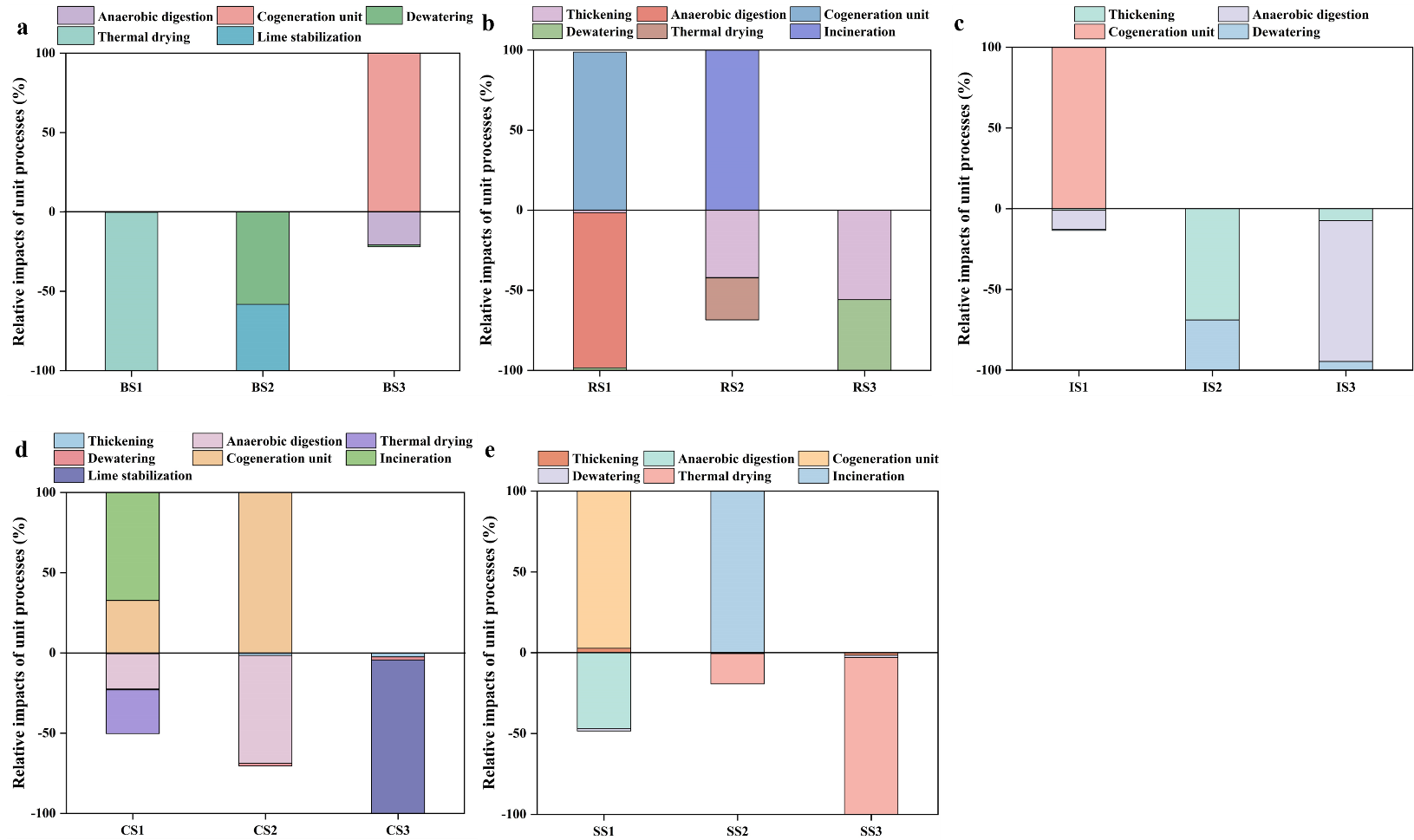


**Fig. S6.** Relative impacts of unit processes on the net energy balance in each sludge management scenario implemented in each BRICS country, namely Brazil (**a**), Russia (**b**), India (**c**), China (**d**), and South Africa (**e**). BS1–3, RS1–3, IS1–3, CS1–3, and SS1–3 represent sludge management scenarios used in Brazil, Russia, India, China, and South Africa, respectively; details of the processes are given in Fig. 2. Positive values indicate processes that facilitate energy production, while negative values indicate processes that result in energy consumption during sludge management.

**
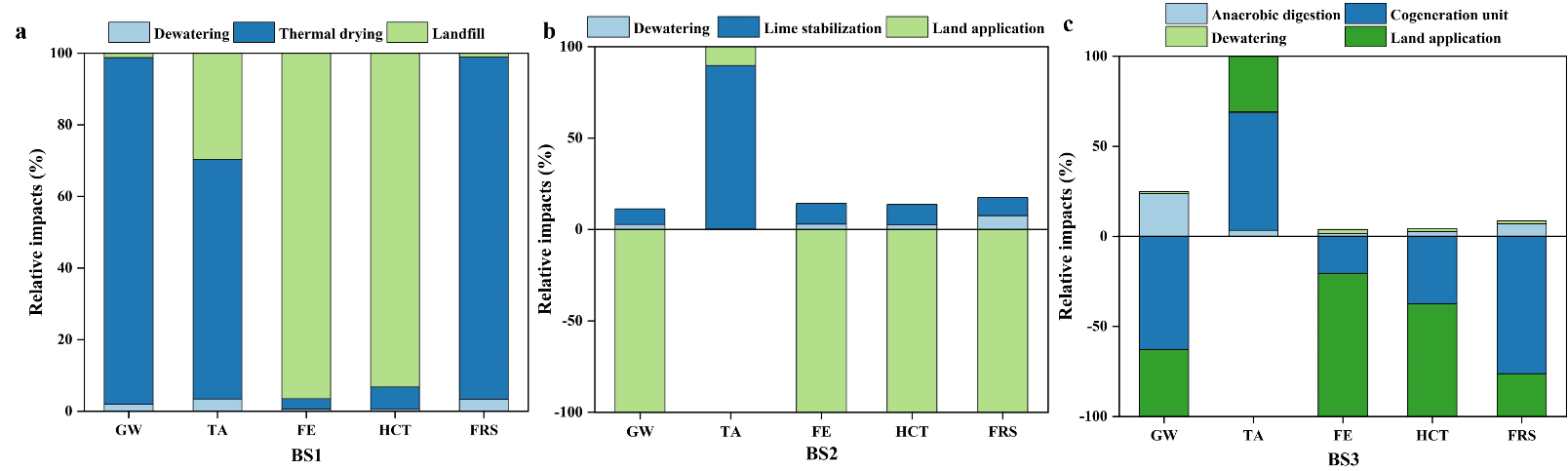
**

**Fig. S7.** Relative impact of each unit process on the total environmental impacts of three sludge management scenarios implemented in Brazil: BS1 (**a**), BS2 (**b**), and BS3 (**c**). Details of the processes used in BS1-3 are given in Fig. 2. GW, TA, FE, HCT, and FRS represent the impact categories of global warming, terrestrial acidification, freshwater eutrophication, human carcinogenic toxicity, and fossil resource scarcity, respectively. Positive values indicate processes that impose environmental burdens, while negative values indicate processes that result in environmental benefits during sludge management.


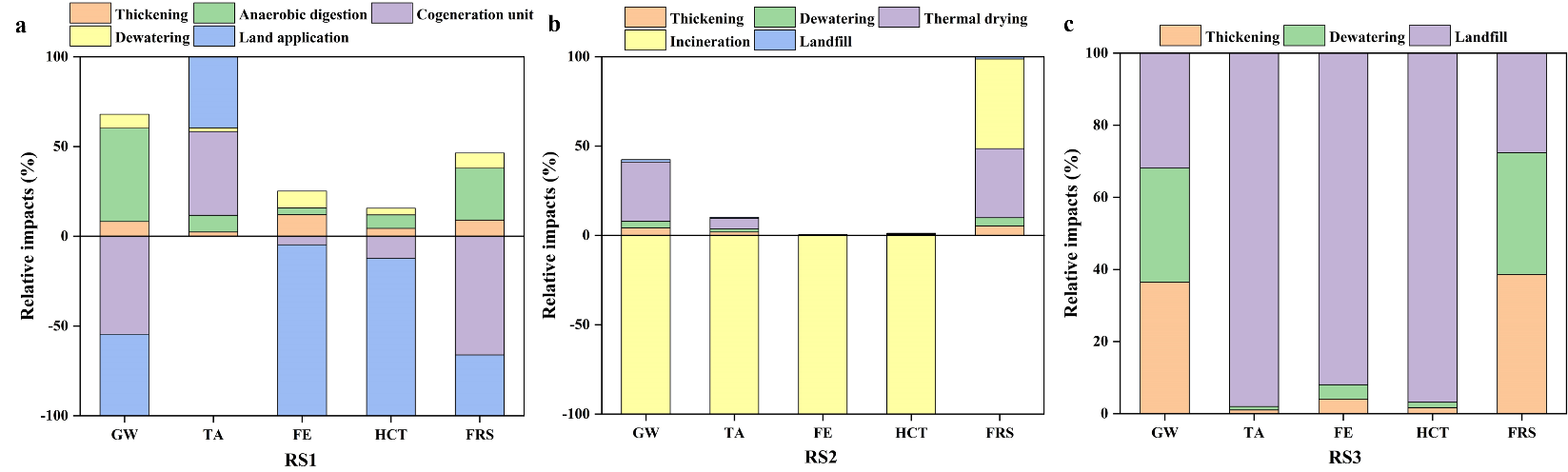


**Fig. S8.** Relative impact of each unit process on the total environmental impacts of three sludge management scenarios implemented in Russia: RS1 (**a**), RS2 (**b**), and RS3 (**c**). Details of the processes included in RS1, RS2, and RS3 are listed in Fig. 2. GW, TA, FE, HCT, and FRS represent the impact categories of global warming, terrestrial acidification, freshwater eutrophication, human carcinogenic toxicity, and fossil resource scarcity, respectively. Positive values indicate processes that impose environmental burdens, while negative values indicate processes that result in environmental benefits during sludge management.


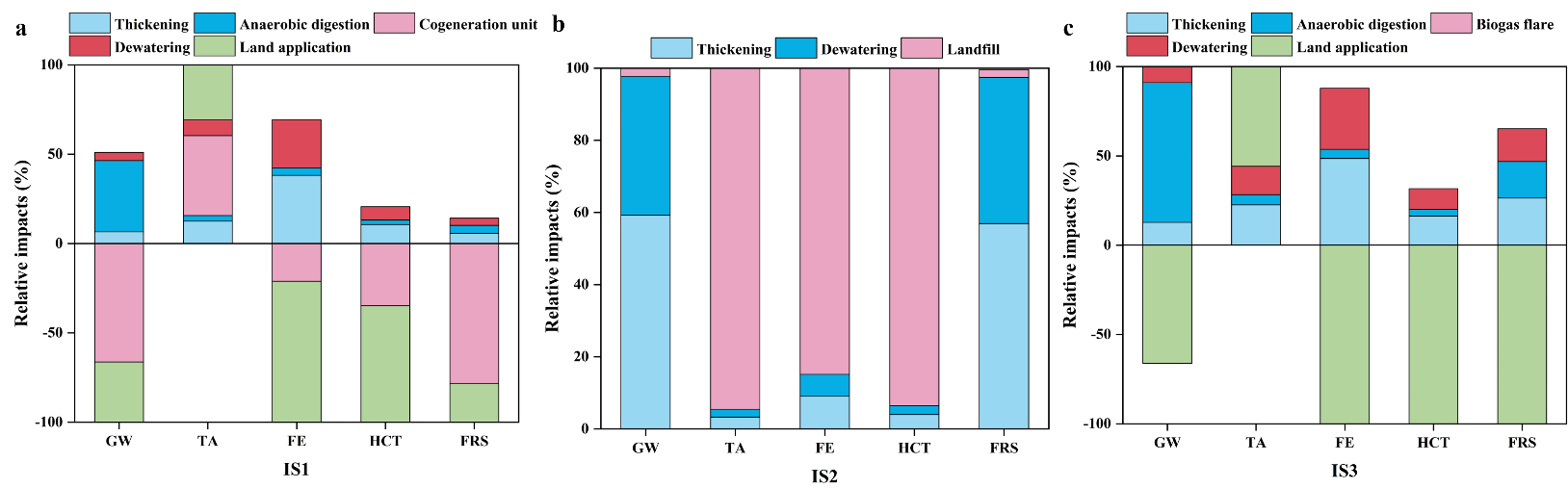


**Fig. S9.** Relative impact of each unit process on the total environmental impacts of three sludge management scenarios implemented in India: IS1 (**a**), IS2 (**b**), and IS3 (**c**). Details of the processes included in IS1, IS2, and IS3 are given in Fig. 2. GW, TA, FE, HCT, and FRS represent the impact categories of global warming, terrestrial acidification, freshwater eutrophication, human carcinogenic toxicity, and fossil resource scarcity, respectively. Positive values indicate processes that impose environmental burdens, while negative values indicate processes that result in environmental benefits during sludge management.


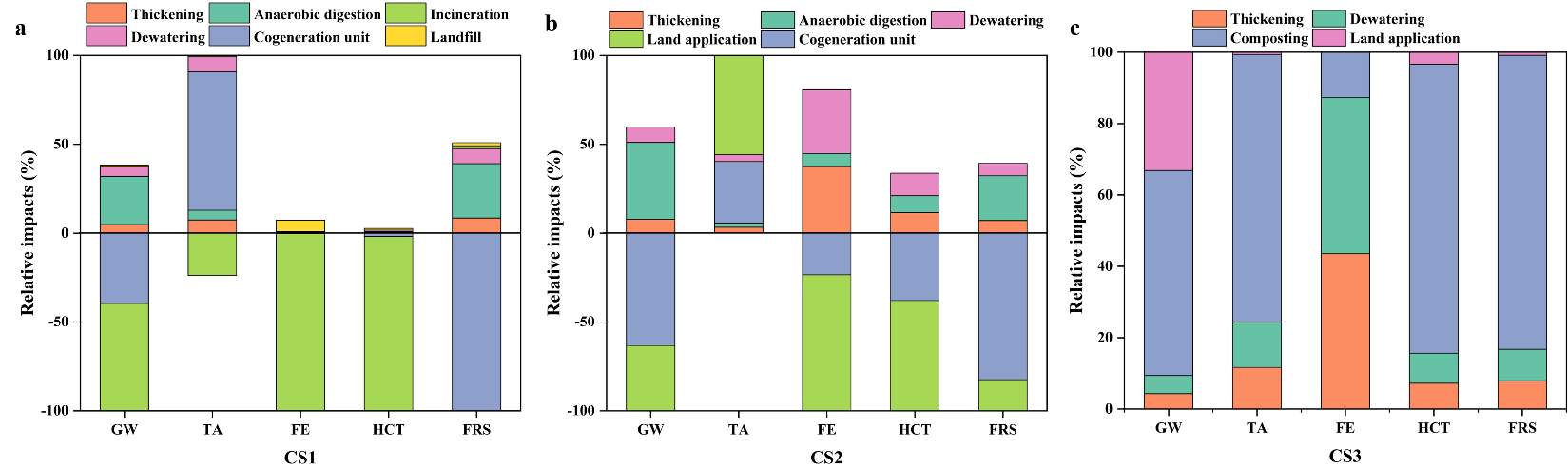


**Fig. S10.** Relative impact of each unit process on the total environmental impacts of three sludge management scenarios implemented in China: CS1 (**a**), CS2 (**b**), and CS3 (**c**). Details of the processes included in each scenario are given in Fig. 2. GW, TA, FE, HCT, and FRS represent the impact categories of global warming, terrestrial acidification, freshwater eutrophication, human carcinogenic toxicity, and fossil resource scarcity, respectively. Positive values indicate processes that impose environmental burdens, while negative values indicate processes that result in environmental benefits during sludge management.


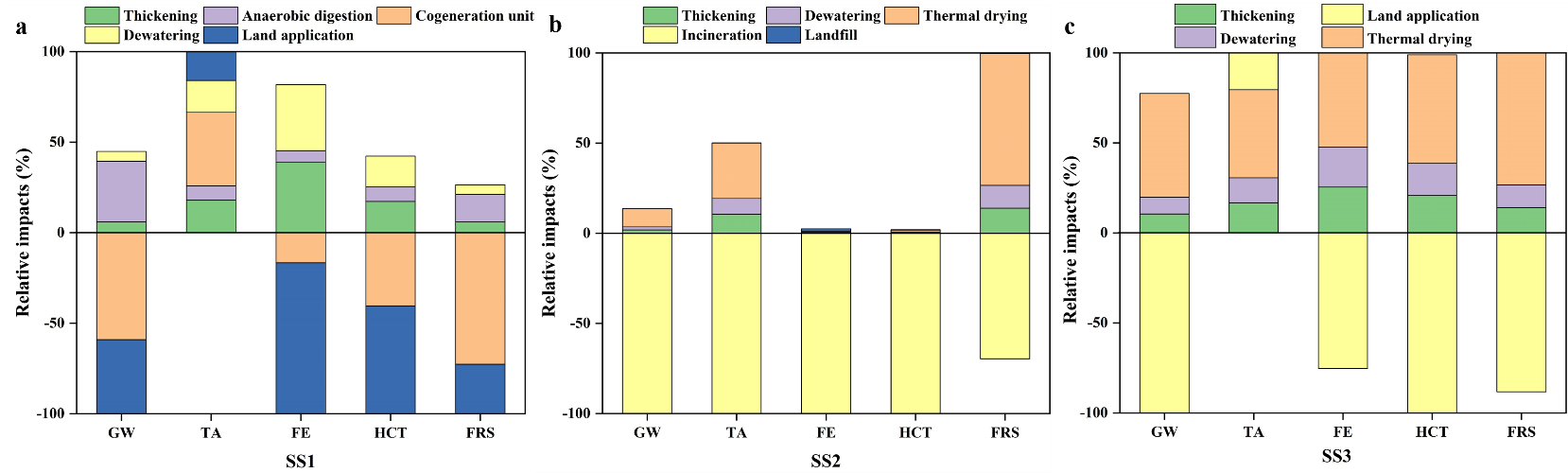


**Fig. S11.** Relative impact of each unit process on the total environmental impacts of three sludge management scenarios implemented in South Africa: SS1 (**a**), SS2 (**b**), and SS3 (**c**). Details of the processes included in each scenario are listed in Fig. 2. GW, TA, FE, HCT, and FRS represent the impact categories of global warming, terrestrial acidification, freshwater eutrophication, human carcinogenic toxicity, and fossil resource scarcity, respectively. Positive values indicate processes that impose environmental burdens, while negative values indicate processes that result in environmental benefits during sludge management.


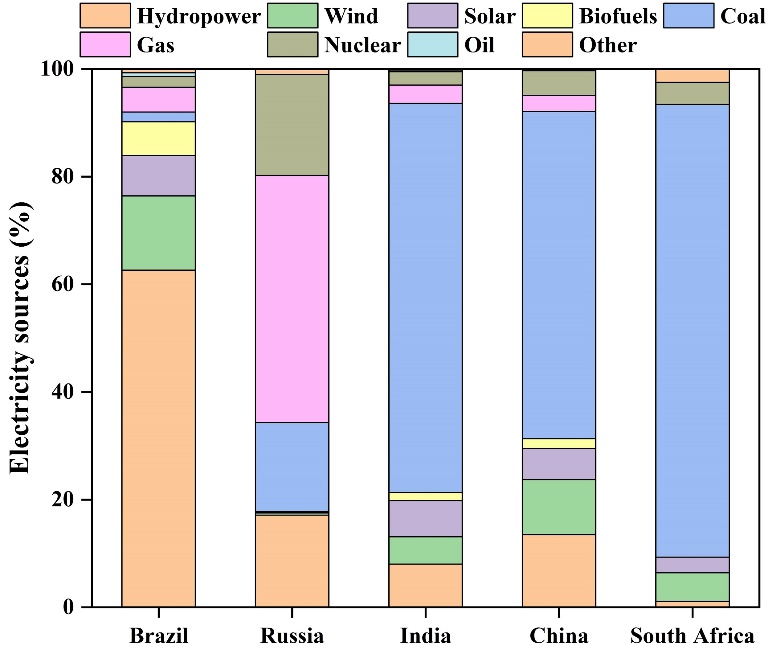


**Fig. S12.** Electricity generation sources in BRICS countries, namely Brazil, Russia, India, China, and South Africa, in 2023 (sourced from https://lowcarbonpower.org/).


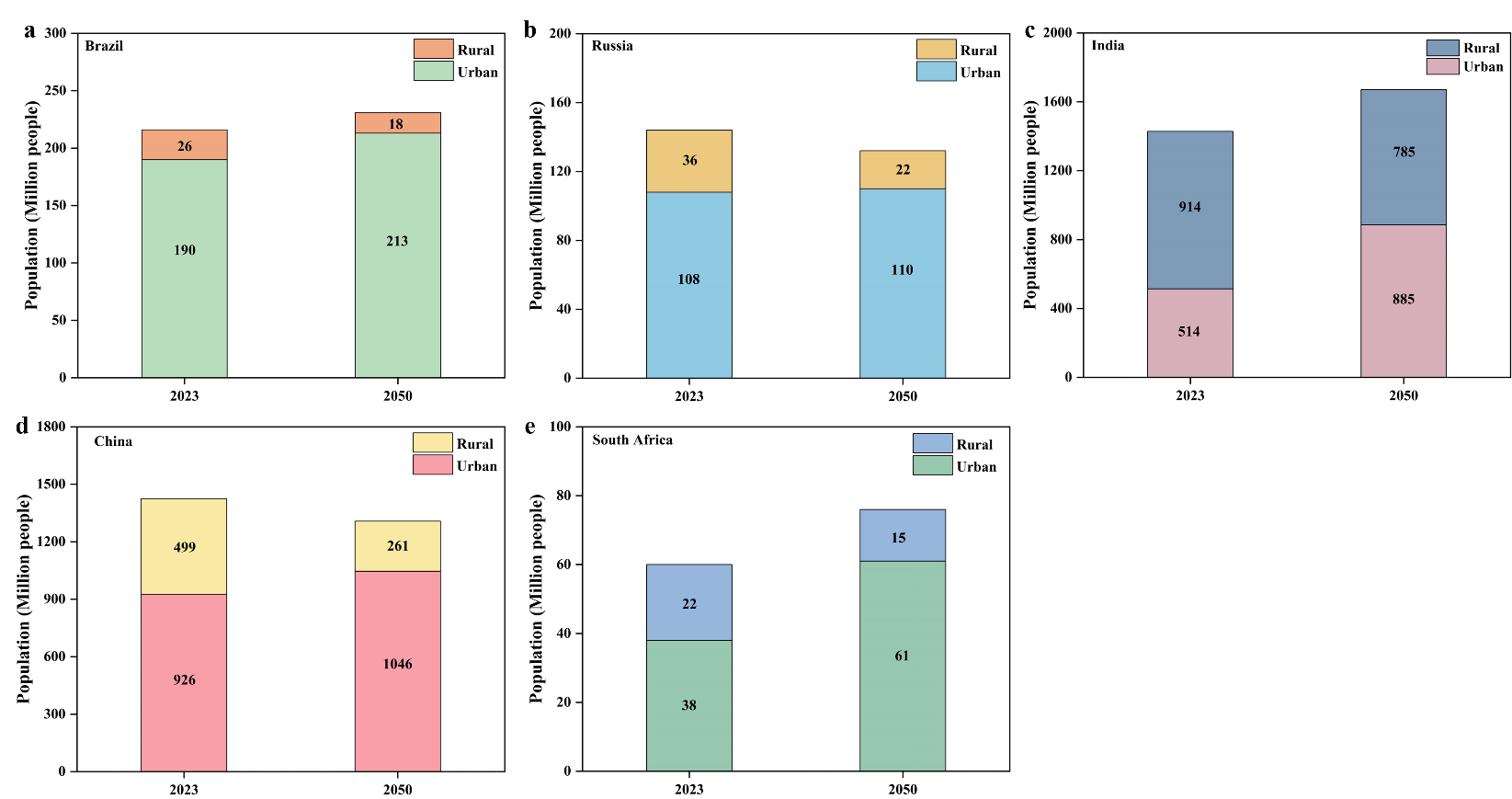


**Fig. S13.** Estimated urban and rural populations of BRICS countries, namely Brazil (**a**), Russia (**b**), India (**c**), China (**d**), and South Africa (**e**), in 2023 and 2050. The data were sourced from the United Nations Department of Economic and Social Affairs.


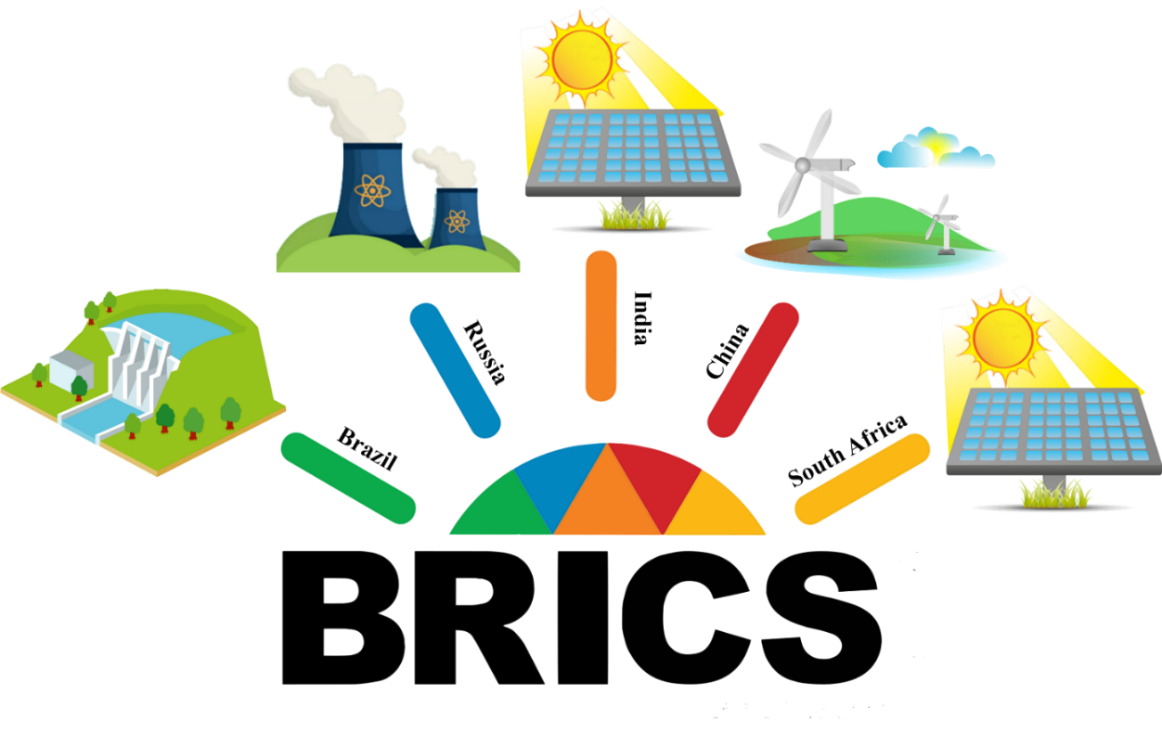


**Fig. S14.** Projected electricity generation sources in BRICS countries, namely Brazil, Russia, India, China, and South Africa, in 2050. The data were sourced from the International Energy Agency.


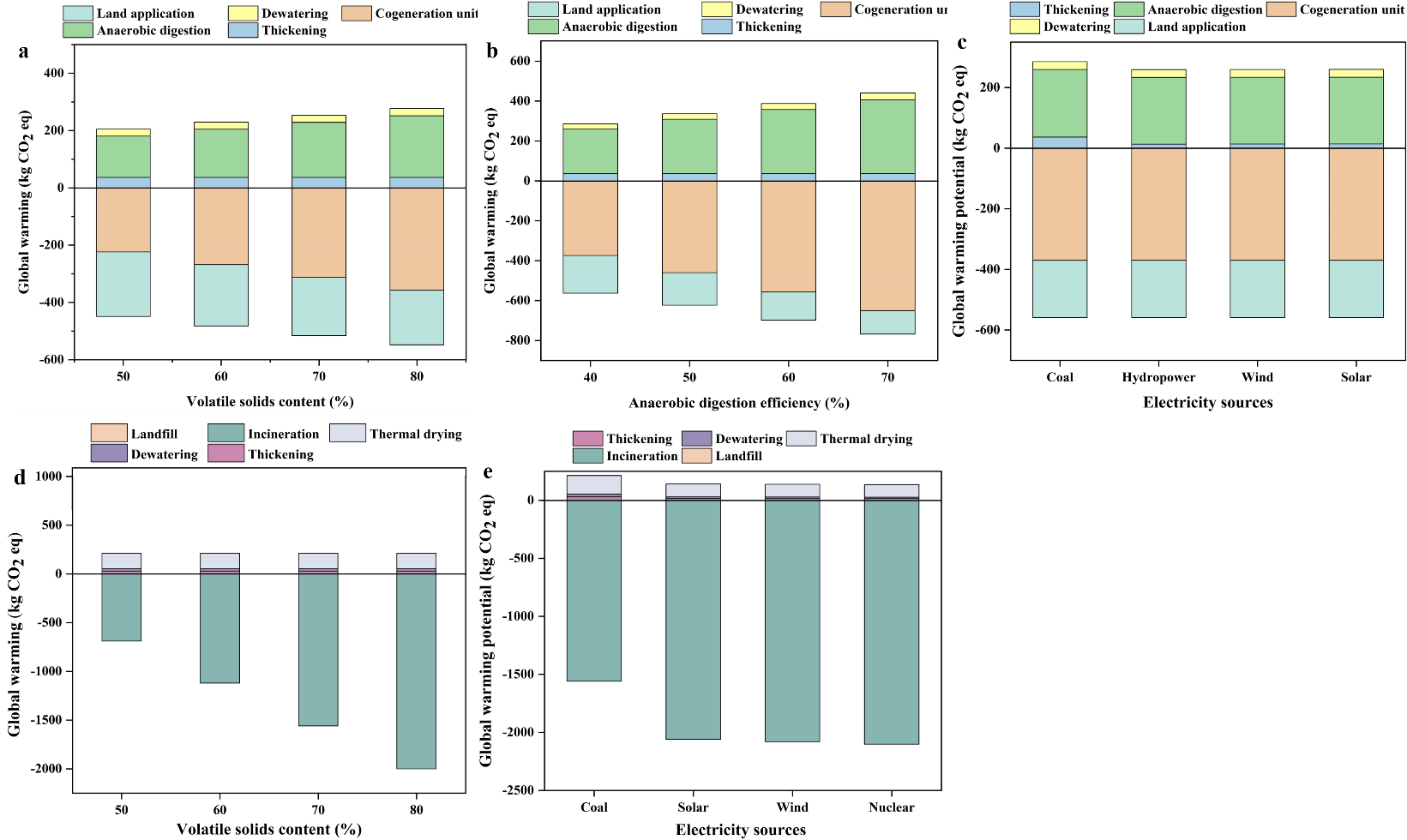


**Fig. S15.** Results of sensitivity analysis of carbon emissions for each unit process in the optimal scenarios for sludge management in India (IS1: Thickening–Anaerobic digestion–Cogeneration unit–Dewatering–Land application) and South Africa (SS2: Thickening–Dewatering–Thermal Drying–Incineration–Landfill). **a–c** Crucial parameters for IS1, namely volatile solids content ranging from 50% to 80% (**a**), anaerobic digestion efficiency ranging from 40% to 70% (**b**) and electricity sources such as coal, hydropower, wind, and solar (**c**). **d–e** Critical parameters for SS2, namely volatile solids content ranging from 50% to 80% (**d**) and electricity sources such as coal, solar, wind, and nuclear (**e**). Positive values indicate processes that facilitate greenhouse gas emissions, while negative values indicate processes associated with greenhouse gas emission mitigation during sludge management.


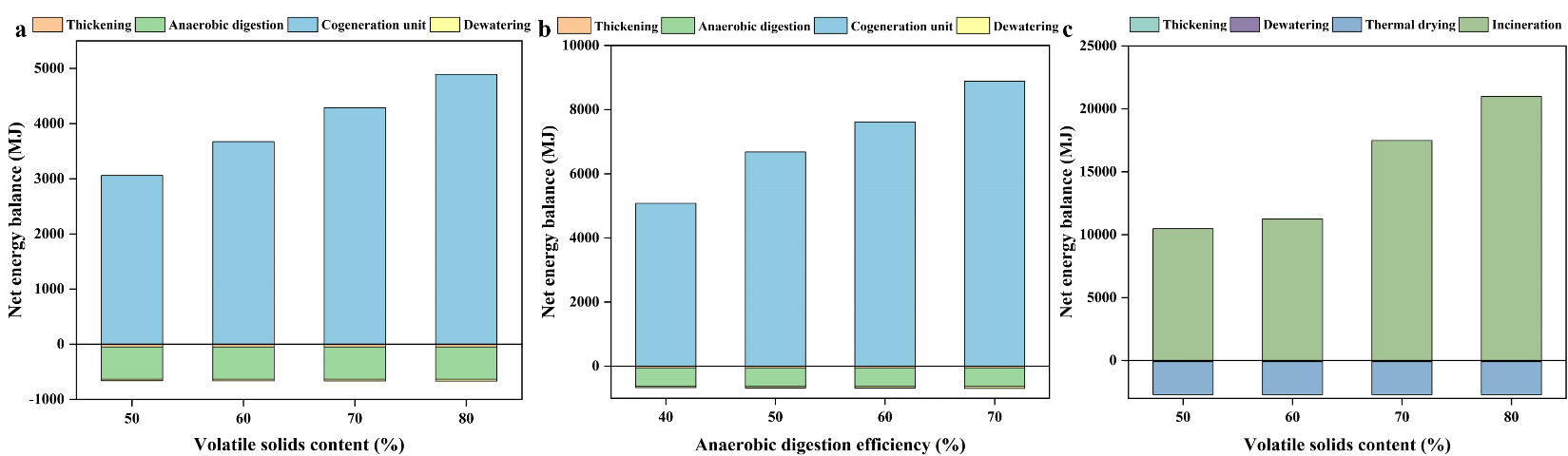


**Fig. S16.** Results of sensitivity analysis of the net energy balance for each unit process in the optimal scenarios for sludge management in India (IS1: Thickening–Anaerobic digestion–Cogeneration unit–Dewatering–Land application) and South Africa (SS2: Thickening–Dewatering–Thermal Drying–Incineration–Landfill). **a–b** Crucial parameters for IS1, namely volatile solids content ranging from 50% to 80% (**a**) and an anaerobic digestion efficiency ranging from 40% to 70% (**b**). **c** The crucial parameter for SS2, namely volatile solids content ranging from 50% to 80%. Positive values indicate processes that facilitate energy production, while negative values indicate processes that consume energy during sludge management.


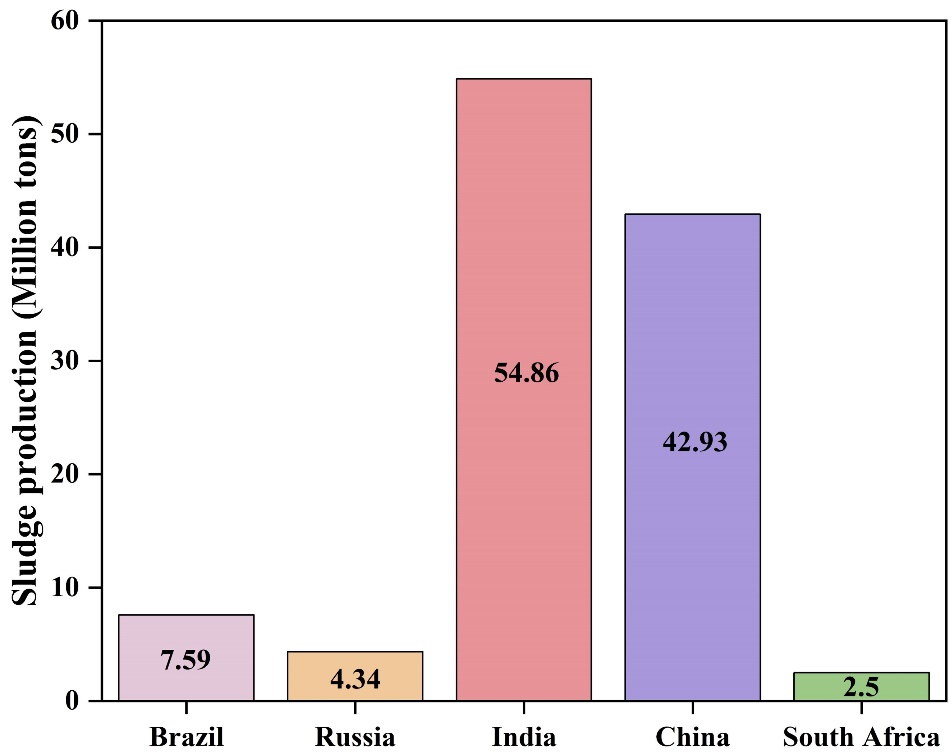


**Fig. S17.** Projected sludge production in BRICS countries, namely Brazil, Russia, India, China, and South Africa, according to the population size and per capita sludge production. Note: Population size data were obtained from the United Nations. Per capita sludge production estimates were based on published research (Appels et al., 2008).


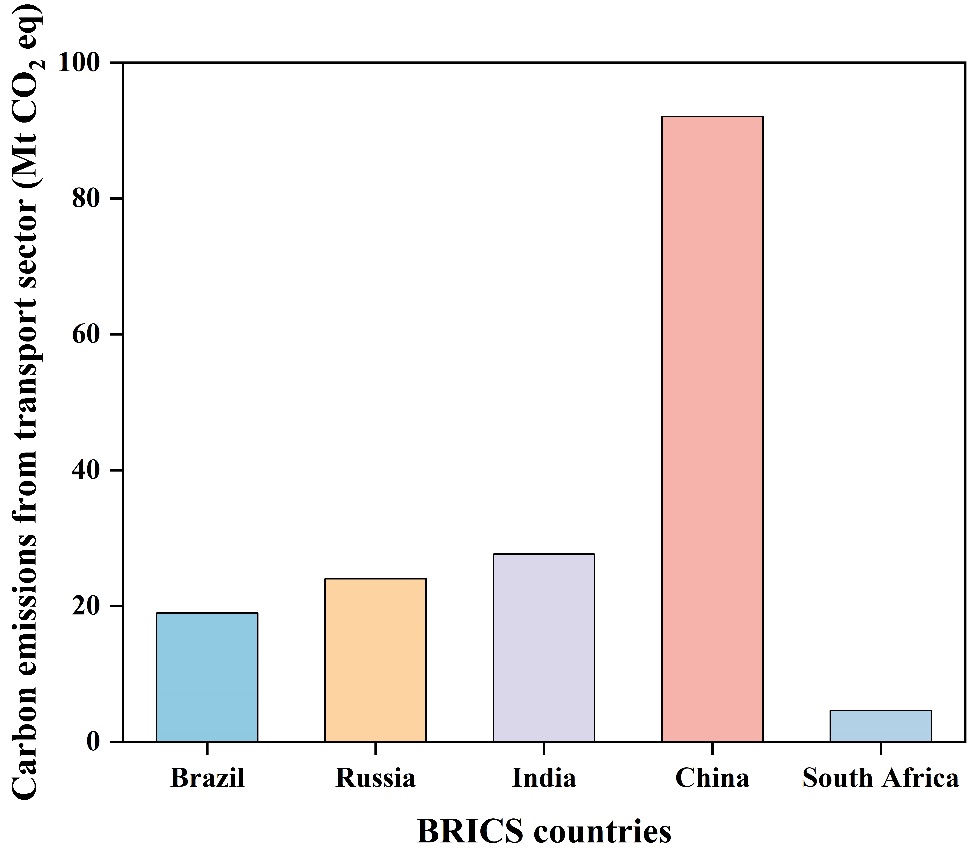


**Fig. S18.** Carbon emissions from the transport sector in BRICS countries. Note: The data in this figure were obtained from Our World in Data (<https://ourworldindata.org/>).


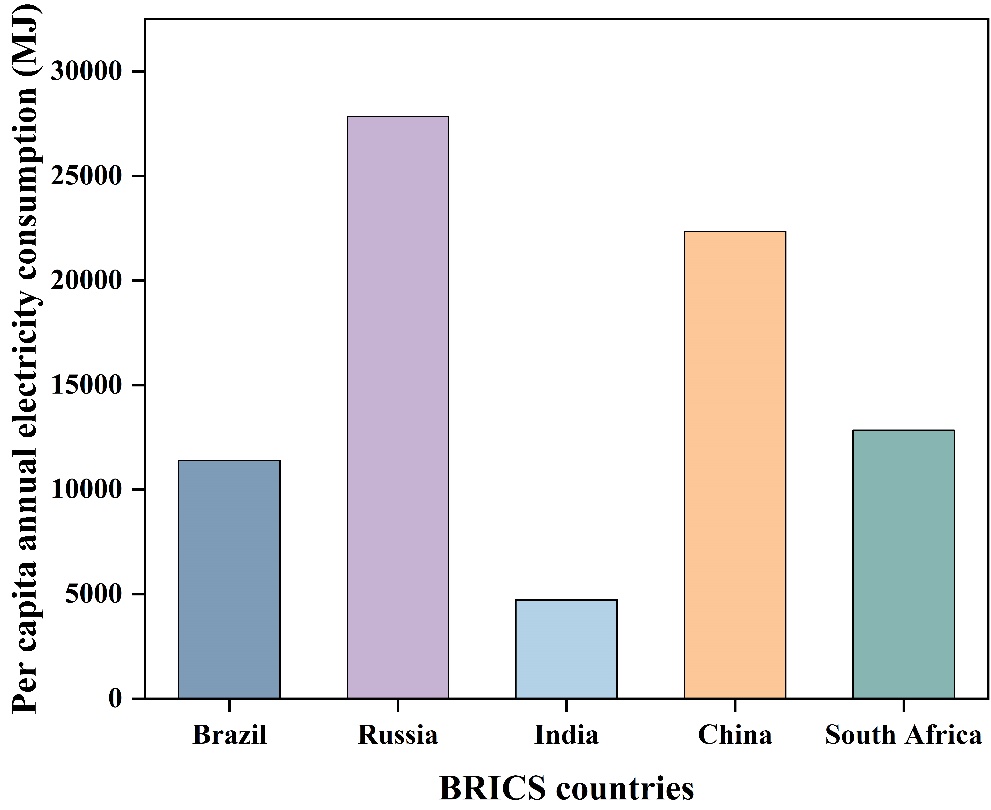


**Fig. S19.** Per capita annual electricity consumption in BRICS countries. Note: The data in this figure were obtained from Our World in Data (<https://ourworldindata.org/>).


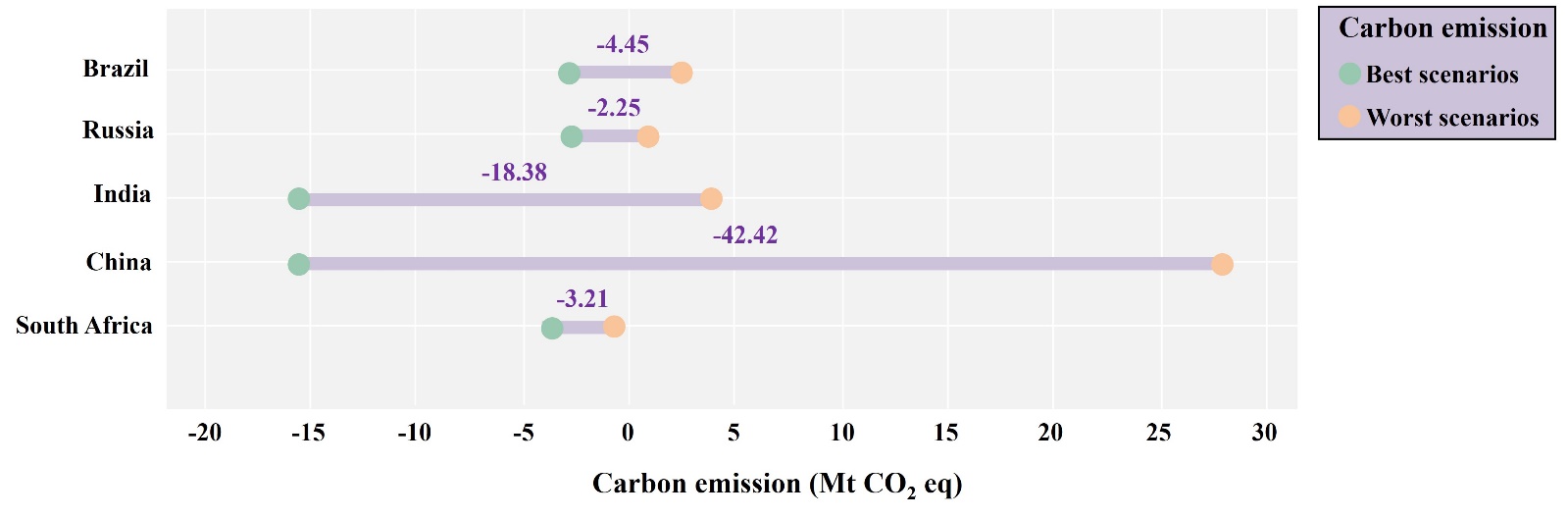


**Fig. S20.** Carbon emission projections for the best and worst sludge management scenarios in each BRICS country for the years 2050, maintaining the same composition of energy sources as in 2023. The purple values represent the difference between the best and worst sludge management scenarios in terms of the carbon emission across the BRICS countries in 2050 (for detailed calculations, see Methods). Negative carbon emission values represent environmental benefits, whereas positive values represent environmental burdens.

# Supplementary Tables

Table S1. Characteristics of sludge sourced from the sewage treatment plants considered in the selected scenarios used in BRICS countries.

| Characteristics | BRICS countries | | | | |
| --- | --- | --- | --- | --- | --- |
|  | Brazil | Russia | India | China | South Africa |
| Moisture content of influent sludge (%) | 93.5 | 99 | 99 | 99 | 99 |
| Moisture content of thickening sludge (%) | / | 95 | 92 | 95 | 95 |
| Moisture content of digested sludge (%) | / | 96.15 | 94.64 | 96 | 96.4 |
| Moisture content of dewatering sludge (%) | 77 (60) | 60 | 60 | 60 | 60 |
| Volatile solids content of raw sludge (%) | 56 | 65 | 83 | 50 | 70 |
| Digestion efficiency (%) | 40 | 35 | 40 | 40 | 40 |
| Moisture content of thermal drying sludge (%) | 60 | 60 | / | 40 | 60 |
| Moisture content of composting sludge (%) | / | / | / | 70 | / |
| Moisture content of incinerated sludge (%) | / | 40 | / | 40 | 40 |

Note: The notation ‘77 (60)’ means that the moisture content of the dewatering sludge was 77% in scenarios 1–2 and 60% in scenario 3 during sludge treatment and disposal in Brazil. The differences in moisture contents across the sludge management scenarios are due to differences in the location of the dewatering processes, as outlined in Supplementary Fig. 1 with specific details. The symbol ‘/’ indicates the absence of a process in the selected sludge treatment and disposal practices used in BRICS countries.

**Table S2****.** Results of life cycle inventories of the sludge treatment and disposal scenarios used in Brazil. The values are displayed per functional unit of 1 t dry sludge.

| Processes | Types | Flows | Quantity | Unit |
| --- | --- | --- | --- | --- |
| Dewatering  (BS1 and BS2) | Input | Raw sludge | 1 | t |
|  | Input | Electricity | 6.97 | kWh |
|  | Input | Polypropylene (flocculant) | 2 | kg |
|  | Output | Dewatered sludge | 1 | t |
| Thermal drying (BS1) | Input | Dewatered sludge | 1 | t |
|  | Input | Heat | 6639.64 | MJ |
|  | Input | Electricity | 101.75 | kWh |
|  | Output | Thermal drying sludge | 1 | t |
| Landfill (BS1) | Input | Thermal drying sludge | 1 | t |
|  | Input | Transportation | 27 | tkm |
|  | Emission | Nitrogen oxides (air) | 1.63E-3 | kg |
|  | Emission | Carbon monoxide (air) | 1.9E-3 | kg |
|  | Emission | Particulates (air) | 6.13E-4 | kg |
|  | Emission | Dioxins (air) | 1.73E-11 | kg |
|  | Emission | Sulfur dioxide (air) | 8E-2 | kg |
|  | Emission | HCl (air) | 4E-2 | kg |
|  | Emission | HF (air) | 8E-2 | kg |
|  | Emission | Cadmium (water) | 0.02 | kg |
|  | Emission | Mercury (water) | 0.025 | kg |
|  | Emission | Lead (water) | 1 | kg |
|  | Emission | Chromium (water) | 1 | kg |
|  | Emission | Arsenic (water) | 0.075 | kg |
|  | Emission | Nickel (water) | 0.2 | kg |
|  | Emission | Zinc (water) | 4 | kg |
|  | Emission | Copper (water) | 1.5 | kg |
|  | Emission | COD (water) | 8.25 | kg |
| Lime stabilization  (BS2) | Input | Dewatered sludge | 1 | t |
|  | Input | Lime | 0.3 | t |
|  | Input | Transportation | 30.6 | tkm |
|  | Input | Electricity | 5 | kWh |
|  | Emission | Ammonia | 17.4 | kg |
|  | Emission | Carbon dioxide | 28.2 | kg |
|  | Emission | Carbon monoxide | 5.39E-2 | kg |
|  | Emission | Nitrogen oxides | 0.12 | kg |
|  | Emission | Particulates | 1.12E-3 | kg |
|  | Output | Stabilized sludge | 1.3 | t |
| Land application  (BS2) | Input | Stabilized sludge | 1.3 | t |
|  | Input | Transportation | 243.1 | tkm |
|  | Output | N_fertilizer (urea) | 10.2 | kg |
|  | Output | P-fertilizer (P_2_O_5_) | 3.6 | kg |
|  | Emission | Arsenic (soil) | 2.05E-2 | kg |
|  | Emission | Barium (soil) | 0.65 | kg |
|  | Emission | Cadmium (soil) | 1.95E-2 | kg |
|  | Emission | Chromium (soil) | 0.5 | kg |
|  | Emission | Copper (soil) | 0.75 | kg |
|  | Emission | Mercury (soil) | 8.5E-3 | kg |
|  | Emission | Molybdenum (soil) | 2.5E-3 | kg |
|  | Emission | Nickel (soil) | 0.21 | kg |
|  | Emission | Lead (soil) | 0.15 | kg |
|  | Emission | Selenium (soil) | 5E-2 | kg |
|  | Emission | Zinc (soil) | 1.4 | kg |
|  | Emission | Ammonia (air) | 5.1 | kg |
|  | Emission | Dinitrogen oxide (air) | 0.15 | kg |
|  | Emission | Nitrogen oxides (air) | 0.057 | kg |
|  | Emission | Carbon monoxide (air) | 0.39 | kg |
|  | Emission | Particulates (air) | 8.54E-5 | kg |
|  | Emission | Phosphate (water) | 0.057 | kg |
|  | Emission | Nitrate (water) | 0.75 | kg |
|  | Emission | Cadmium (water) | 2.73E-6 | kg |
|  | Emission | Copper (water) | 2.46E-4 | kg |
|  | Emission | Zinc (water) | 2.19E-3 | kg |
|  | Emission | Lead (water) | 3.54E-5 | kg |
|  | Emission | Chromium (water) | 1.42E-3 | kg |
|  | Emission | Mercury (water) | 8.9E-8 | kg |
| Anaerobic digestion  (BS3) | Input | Raw sludge | 1 | t |
|  | Input | Heat | 744.4 | MJ |
|  | Input | Electricity | 1.95 | kWh |
|  | Emission | Methane | 2.14 | kg |
|  | Emission | Carbon dioxide | 2.73 | kg |
|  | Emission | Nitrogen | 0.08 | kg |
|  | Emission | Hydrogen | 6.2E-3 | kg |
|  | Emission | Hydrogen sulfide | 3.5E-2 | kg |
|  | Output | Digested sludge | 0.78 | t |
|  | Output | Biogas | 166.09 | m^3^ |
| Cogeneration unit (BS3) | Input | Biogas | 166.09 | m^3^ |
|  | Input | Lubricating oil | 0.11 | kg |
|  | Output | Electricity | 445.2 | kWh |
|  | Output | Heat | 2003.40 | MJ |
|  | Output | Waste oil | 0.11 | kg |
|  | Emission | Carbon dioxide | 300.96 | kg |
|  | Emission | Methane | 1.2 | kg |
|  | Emission | Carbon monoxide | 1.12 | kg |
|  | Emission | Sulfur dioxide | 6.9E-2 | kg |
|  | Emission | Nitrogen oxides | 0.73 | kg |
|  | Emission | Dinitrogen oxides | 5.8E-3 | kg |
|  | Emission | Particulates | 1.2E-3 | kg |
| Dewatering (BS3) | Input | Digested sludge | 0.776 | t |
|  | Input | Electricity | 10.93 | kWh |
|  | Input | Polypropylene (flocculant) | 1.55 | kg |
|  | Output | Dewatered sludge | 0.78 | t |
| Land application  (BS3) | Input | Dewatered sludge | 0.78 | t |
|  | Input | Transportation | 145.11 | tkm |
|  | Output | N_fertilizer (urea) | 7.92 | kg |
|  | Output | P-fertilizer (P_2_O_5_) | 2.79 | kg |
|  | Emission | Arsenic (soil) | 1.59E-2 | kg |
|  | Emission | Barium (soil) | 0.50 | kg |
|  | Emission | Cadmium (soil) | 1.51E-2 | kg |
|  | Emission | Chromium (soil) | 0.39 | kg |
|  | Emission | Copper (soil) | 0.58 | kg |
|  | Emission | Mercury (soil) | 6.6E-3 | kg |
|  | Emission | Molybdenum (soil) | 1.94E-2 | kg |
|  | Emission | Nickel (soil) | 0.16 | kg |
|  | Emission | Lead (soil) | 0.12 | kg |
|  | Emission | Selenium (soil) | 3.88E-2 | kg |
|  | Emission | Zinc (soil) | 1.09 | kg |
|  | Emission | Ammonia (air) | 3.96 | kg |
|  | Emission | Dinitrogen oxide (air) | 0.12 | kg |
|  | Emission | Nitrogen oxides (air) | 0.044 | kg |
|  | Emission | Carbon monoxide (air) | 0.03 | kg |
|  | Emission | Particulates (air) | 6.63E-5 | kg |
|  | Emission | Phosphate (water) | 0.044 | kg |
|  | Emission | Nitrate (water) | 0.58 | kg |
|  | Emission | Cadmium (water) | 2.12E-6 | kg |
|  | Emission | Copper (water) | 1.91E-4 | kg |
|  | Emission | Zinc (water) | 1.7E-3 | kg |
|  | Emission | Lead (water) | 2.75E-5 | kg |
|  | Emission | Chromium (water) | 1.1E-3 | kg |
|  | Emission | Mercury (water) | 6.91E-8 | kg |

**Table S3.** Results of life cycle inventories of the sludge treatment and disposal scenarios used in Russia. The values are displayed per functional unit of 1 t dry sludge.

| Processes | Types | Flows | Quantity | Unit |
| --- | --- | --- | --- | --- |
| Thickening  (RS1–3) | Input | Raw sludge | 1 | t |
|  | Input | Electricity | 14 | kWh |
|  | Input | Polyacrylamide (flocculant) | 4 | kg |
|  | Output | Thickening sludge | 1 | t |
| Anaerobic digestion  (RS1) | Input | Thickening sludge | 1 | t |
|  | Input | Heat | 3149.97 | MJ |
|  | Input | Electricity | 1.95 | kWh |
|  | Emission | Methane | 3.07 | kg |
|  | Emission | Carbon dioxide | 1.42 | kg |
|  | Emission | Nitrogen | 7.1E-2 | kg |
|  | Emission | Hydrogen | 7.1E-2 | kg |
|  | Emission | Hydrogen sulfide | 2.4E-3 | kg |
|  | Output | Digested sludge | 0.77 | t |
|  | Output | Biogas | 152.74 | m^3^ |
| Cogeneration unit (RS1) | Input | Biogas | 152.74 | m^3^ |
|  | Input | Lubricating oil | 0.093 | kg |
|  | Output | Electricity | 397.12 | kWh |
|  | Output | Heat | 1790 | MJ |
|  | Output | Waste oil | 0.093 | kg |
|  | Emission | Carbon dioxide | 269.19 | kg |
|  | Emission | Methane | 1.08 | kg |
|  | Emission | Carbon monoxide | 1.0 | kg |
|  | Emission | Sulfur dioxide | 6.2E-2 | kg |
|  | Emission | Nitrogen oxides | 0.65 | kg |
|  | Emission | Dinitrogen oxides | 5.2E-3 | kg |
|  | Emission | Particulates | 1.1E-3 | kg |
| Dewatering (RS1) | Input | Digested sludge | 0.77 | t |
|  | Input | Electricity | 14.84 | kWh |
|  | Input | Polyacrylamide (flocculant) | 3.08 | kg |
|  | Output | Dewatered sludge | 0.77 | t |
| Land application  (RS1) | Input | Dewatered sludge | 0.77 | t |
|  | Input | Transportation | 84.7 | tkm |
|  | Output | K-fertilizer (potassium nitrate) | 5.39 | kg |
|  | Output | N_fertilizer (calcium ammonium nitrate) | 11.55 | kg |
|  | Output | P-fertilizer (diammonium phosphate) | 34.65 | kg |
|  | Emission | Cadmium (soil) | 1.2E-2 | kg |
|  | Emission | Mercury (soil) | 5.8E-3 | kg |
|  | Emission | Lead (soil) | 0.19 | kg |
|  | Emission | Chromium (soil) | 0.39 | kg |
|  | Emission | Arsenic (soil) | 7.7E-3 | kg |
|  | Emission | Nickel (soil) | 0.15 | kg |
|  | Emission | Zinc (soil) | 1.35 | kg |
|  | Emission | Copper (soil) | 0.58 | kg |
|  | Emission | Ammonia (air) | 3.93 | kg |
|  | Emission | Dinitrogen oxide (air) | 0.12 | kg |
|  | Emission | Nitrogen oxides (air) | 0.044 | kg |
|  | Emission | Carbon monoxide (air) | 0.03 | kg |
|  | Emission | Particulates (air) | 6.58E-5 | kg |
|  | Emission | Phosphate (water) | 0.044 | kg |
|  | Emission | Nitrate (water) | 0.58 | kg |
|  | Emission | Cadmium (water) | 2.1E-6 | kg |
|  | Emission | Copper (water) | 1.89E-4 | kg |
|  | Emission | Zinc (water) | 1.69E-3 | kg |
|  | Emission | Lead (water) | 2.73E-5 | kg |
|  | Emission | Chromium (water) | 1.09E-3 | kg |
|  | Emission | Mercury (water) | 6.95E-8 | kg |
| Dewatering  (RS2 and RS3) | Input | Thickening sludge | 1 | t |
|  | Input | Electricity | 11.06 | kWh |
|  | Input | Polyacrylamide (flocculant) | 4 | kg |
|  | Output | Dewatered sludge | 1 | t |
| Thermal drying  (RS2)  Incineration  (RS2) | Input | Dewatered sludge | 1 | t |
|  | Input | Heat | 2942.9 | MJ |
|  | Input | Electricity | 45.65 | kWh |
|  | Input | Drying sludge | 1 | t |
|  | Input | NaOH | 10.02 | kg |
|  | Input | NaHCO_3_ | 31.48 | kg |
|  | Input | Lime | 61.43 | kg |
|  | Input | Activated carbon | 1.4 | kg |
|  | Input | Electricity | 485.33 | kWh |
|  | Output | Electricity | 1891.67 | kWh |
|  | Output | Heat | 6810 | MJ |
|  | Output | Drying sludge | 1 | t |
|  | Emission | Carbon monoxide | 3.3E-2 | kg |
|  | Emission | Nitrogen oxide | 0.33 | kg |
|  | Emission | Ammonia | 2E-2 | kg |
|  | Emission | Sulfur dioxide | 2E-2 | kg |
|  | Emission | PM | 2E-2 | kg |
| Landfill (Incinerated sludge) (RS2) | Input | Mass of ash | 0.35 | t |
|  | Input | Transportation | 63 | tkm |
|  | Emission | Aluminium (air) | 3.85E-4 | kg |
|  | Emission | Arsenic (air) | 8.12E-7 | kg |
|  | Emission | Cadmium (air) | 7.21E-9 | kg |
|  | Emission | Calcium (air) | 3.57E-4 | kg |
|  | Emission | Carbon monoxide, fossil | 1.59E-3 | kg |
|  | Emission | Chloride (air) | 8.05 | kg |
|  | Emission | Chromium (air) | 1.42E-11 | kg |
|  | Emission | Copper (air) | 9.77E-8 | kg |
|  | Emission | Iron (air) | 4.66E-5 | kg |
|  | Emission | Lead (air) | 8.47E-9 | kg |
|  | Emission | Mercury (air) | 8.23E-11 | kg |
|  | Emission | Methane, fossil (air) | 4.55E-5 | kg |
|  | Emission | Nickel (air) | 1.8E-11 | kg |
|  | Emission | Phosphorus | 2.15E-4 | kg |
|  | Emission | Silicon | 9.98E-4 | kg |
|  | Emission | Zinc | 9.94E-8 | kg |
|  | Emission | Aluminium (water) | 1.13E-2 | kg |
|  | Emission | Arsenic (water) | 2.54E-3 | kg |
|  | Emission | Cadmium (water) | 1.34E-4 | kg |
|  | Emission | Calcium (water) | 1.96 | kg |
|  | Emission | Chromium (water) | 6.13E-7 | kg |
|  | Emission | Copper (water) | 8.86E-5 | kg |
|  | Emission | Iron (water) | 5.39E-2 | kg |
|  | Emission | Lead (water) | 2.81 | kg |
|  | Emission | Mercury (water) | 1.54E-5 | kg |
|  | Emission | Nickel (water) | 6.41E-4 | kg |
|  | Emission | Phosphate | 0.35 | kg |
|  | Emission | Silicon | 2.74E-2 | kg |
|  | Emission | Potassium | 0.63 | kg |
|  | Emission | Sulfate | 1.37 | kg |
|  | Emission | Zinc | 2.72E-3 | kg |
| Landfill (dewatered sludge) (RS3) | Input | Dewatered sludge | 1 | t |
|  | Input | Transportation | 180 | tkm |
|  | Emission | Cadmium (water) | 2E-2 | kg |
|  | Emission | Mercury (water) | 2.5E-2 | kg |
|  | Emission | Lead (water) | 1 | kg |
|  | Emission | Chromium (water) | 1 | kg |
|  | Emission | Arsenic (water) | 7.5E-2 | kg |
|  | Emission | Nickel (water) | 0.2 | kg |
|  | Emission | Zinc (water) | 4 | kg |
|  | Emission | Copper (water) | 1.5 | kg |
|  | Emission | COD | 8.25 | kg |
|  | Emission | Ammonia | 10.08 | kg |
|  | Emission | Sulfur dioxide | 0.22 | kg |
|  | Emission | Nitrogen oxides | 7.8E-2 | kg |
|  | Emission | Hydrogen sulfide | 4.54E-3 | kg |

**Table S4.** Results of life cycle inventories of the sludge treatment and disposal scenarios used in India. The values are displayed per functional unit of 1 t dry sludge.

| Processes | Types | Flows | Quantity | Unit |
| --- | --- | --- | --- | --- |
| Thickening  (IS1–3) | Input | Raw sludge | 1 | t |
|  | Input | Electricity | 14 | kWh |
|  | Input | Polyacrylamide (flocculant) | 4 | kg |
|  | Output | Thickening sludge | 1 | t |
| Anaerobic digestion  (IS1 and IS3) | Input | Thickening sludge | 1 | t |
|  | Input | Heat | 588.66 | MJ |
|  | Input | Electricity | 1.95 | kWh |
|  | Emission | Methane | 5.92 | kg |
|  | Emission | Carbon dioxide | 7.53 | kg |
|  | Emission | Nitrogen | 0.22 | kg |
|  | Emission | Hydrogen | 1.7E-2 | kg |
|  | Emission | Hydrogen sulfide | 9.8E-2 | kg |
|  | Output | Digested sludge | 0.67 | t |
|  | Output | Biogas | 241.16 | m^3^ |
| Cogeneration unit (IS1) | Input | Biogas | 241.16 | m^3^ |
|  | Input | Lubricating oil | 0.15 | kg |
|  | Output | Electricity | 627.02 | kWh |
|  | Output | Heat | 2820 | MJ |
|  | Output | Waste oil | 0.15 | kg |
|  | Emission | Carbon dioxide | 424.69 | kg |
|  | Emission | Methane | 1.70 | kg |
|  | Emission | Carbon monoxide | 1.57 | kg |
|  | Emission | Sulfur dioxide | 9.8E-2 | kg |
|  | Emission | Nitrogen oxides | 1.03 | kg |
|  | Emission | Dinitrogen oxides | 8E-3 | kg |
|  | Emission | Particulates | 1.7E-3 | kg |
| Dewatering  (IS1 and IS3) | Input | Digested sludge | 0.67 | t |
|  | Input | Electricity | 10.21 | kWh |
|  | Input | Polyacrylamide (flocculant) | 2.68 | kg |
|  | Output | Dewatered sludge | 0.67 | t |
| Land application  (IS1 and IS3) | Input | Dewatered sludge | 0.67 | t |
|  | Input | Transportation | 13.4 | tkm |
|  | Output | K-fertilizer (potassium nitrate) | 8.78 | kg |
|  | Output | N_fertilizer (calcium ammonium nitrate) | 16.48 | kg |
|  | Output | P-fertilizer (diammonium phosphate) | 12.26 | kg |
|  | Emission | Cadmium (soil) | 3.3E-3 | kg |
|  | Emission | Lead (soil) | 0.28 | kg |
|  | Emission | Nickel (soil) | 0.12 | kg |
|  | Emission | Zinc (soil) | 0.85 | kg |
|  | Emission | Copper (soil) | 0.2 | kg |
|  | Emission | Ammonia (air) | 3.42 | kg |
|  | Emission | Dinitrogen oxide (air) | 0.1 | kg |
|  | Emission | Nitrogen oxides (air) | 3.82E-2 | kg |
|  | Emission | Carbon monoxide (air) | 2.61E-2 | kg |
|  | Emission | Particulates (air) | 5.72E-5 | kg |
|  | Emission | Phosphate (water) | 3.82E-2 | kg |
|  | Emission | Nitrate (water) | 5.03E-1 | kg |
|  | Emission | Cadmium (water) | 1.83E-6 | kg |
|  | Emission | Copper (water) | 1.65E-4 | kg |
|  | Emission | Zinc (water) | 1.47E-3 | kg |
|  | Emission | Lead (water) | 2.37E-5 | kg |
|  | Emission | Chromium (water) | 9.51E-4 | kg |
|  | Emission | Mercury (water) | 5.96E-8 | kg |
| Dewatering  (IS2) | Input | Thickening sludge | 1 | t |
|  | Input | Electricity | 6.32 | kWh |
|  | Input | Polyacrylamide (flocculant) | 4 | kg |
|  | Output | Dewatered sludge | 1 | t |
| Landfill (dewatered sludge) (IS2) | Input | Dewatered sludge | 1 | t |
|  | Input | Transportation | 8 | tkm |
|  | Emission | Cadmium (water) | 2E-2 | kg |
|  | Emission | Mercury (water) | 2.5E-2 | kg |
|  | Emission | Lead (water) | 1 | kg |
|  | Emission | Chromium (water) | 1 | kg |
|  | Emission | Arsenic (water) | 7.5E-2 | kg |
|  | Emission | Nickel (water) | 0.2 | kg |
|  | Emission | Zinc (water) | 4 | kg |
|  | Emission | Copper (water) | 1.5 | kg |
|  | Emission | COD (water) | 8.25 | kg |
|  | Emission | Ammonia (air) | 10.08 | kg |
|  | Emission | Sulfur dioxide (air) | 0.22 | kg |
|  | Emission | Nitrogen oxides (air) | 7.8E-2 | kg |
|  | Emission | Hydrogen sulfide (air) | 4.54E-3 | kg |
| Biogas flared  (IS3) | Input | Biogas | 241.16 | m^3^ |
|  | Emission | Carbon dioxide | 0.13 | kg |
|  | Emission | Nitrogen oxides | 1.45E-5 | kg |
|  | Emission | Sulfur oxides | 3.62E-5 | kg |

**Table S5.** Results of life cycle inventories of the sludge treatment and disposal scenarios used in China. The values are displayed per functional unit of 1 t dry sludge.

| Processes | Types | Flows | Quantity | Unit |
| --- | --- | --- | --- | --- |
| Thickening  (CS1–3) | Input | Raw sludge | 1 | t |
|  | Input | Electricity | 14 | kWh |
|  | Input | Polyacrylamide (flocculant) | 4 | kg |
|  | Output | Thickening sludge | 1 | t |
| Anaerobic digestion  (CS1 and CS2) | Input | Thickening sludge | 1 | t |
|  | Input | Heat | 2093 | MJ |
|  | Input | Electricity | 1.5 | kWh |
|  | Emission | Methane | 2.14 | kg |
|  | Emission | Carbon dioxide | 2.73 | kg |
|  | Emission | Nitrogen | 8E-2 | kg |
|  | Emission | Hydrogen | 6.2E-3 | kg |
|  | Emission | Hydrogen sulfide | 3.5E-2 | kg |
|  | Output | Digested sludge | 0.8 | t |
|  | Output | Biogas | 148.33 | m^3^ |
| Cogeneration unit (CS1 and CS2) | Input | Biogas | 148.33 | m^3^ |
|  | Input | Lubricating oil | 9.1E-2 | kg |
|  | Output | Electricity | 385.66 | kWh |
|  | Output | Heat | 1735.45 | MJ |
|  | Output | Waste oil | 9.1E-2 | kg |
|  | Emission | Carbon dioxide | 261.67 | kg |
|  | Emission | Methane | 1.05 | kg |
|  | Emission | Carbon monoxide | 0.97 | kg |
|  | Emission | Sulfur dioxide | 0.06 | kg |
|  | Emission | Nitrogen oxides | 0.63 | kg |
|  | Emission | Dinitrogen oxides | 5E-3 | kg |
|  | Emission | Particulates | 1E-3 | kg |
| Dewatering  (CS1 and CS2) | Input | Digested sludge | 0.8 | t |
|  | Input | Electricity | 14.22 | kWh |
|  | Input | Polyacrylamide (flocculant) | 3.2 | kg |
|  | Output | Dewatered sludge | 0.8 | t |
| Thermal drying  (CS1) | Input | Dewatered sludge | 0.8 | t |
|  | Input | Heat | 2471.36 | MJ |
|  | Input | Power | 36.85 | kWh |
|  | Output | Dried sludge | 0.8 | t |
| Incineration (CS1) | Input | Dried sludge | 0.8 | t |
|  | Input | NaOH | 4.62 | kg |
|  | Input | NaHCO_3_ | 14.53 | kg |
|  | Input | Lime | 28.35 | kg |
|  | Input | Activated carbon | 1.12 | kg |
|  | Input | Electricity | 366.67 | kWh |
|  | Output | Electricity | 1077.78 | kWh |
|  | Output | Heat | 3880 | MJ |
|  | Emission | Carbon monoxide | 1.5E-2 | kg |
|  | Emission | Nitrogen oxide | 0.15 | kg |
|  | Emission | Ammonia | 9E-3 | kg |
|  | Emission | Sulfur dioxide | 9E-3 | kg |
|  | Emission | PM | 1.6E-2 | kg |
| Landfill (Incinerated sludge) (CS1) | Input | Mass of ash | 0.5 | t |
|  | Input | Transportation | 29 | tkm |
|  | Emission | Aluminium (air) | 5.5E-4 | kg |
|  | Emission | Arsenic (air) | 1.16E-4 | kg |
|  | Emission | Cadmium (air) | 1.03E-8 | kg |
|  | Emission | Calcium (air) | 5.1E-4 | kg |
|  | Emission | Carbon monoxide, fossil | 2.27E-3 | kg |
|  | Emission | Chloride (air) | 11.5 | kg |
|  | Emission | Chromium (air) | 2.04E-11 | kg |
|  | Emission | Copper (air) | 1.4E-7 | kg |
|  | Emission | Iron (air) | 6.65E-5 | kg |
|  | Emission | Lead (air) | 1.21E-8 | kg |
|  | Emission | Mercury (air) | 1.18E-10 | kg |
|  | Emission | Methane, fossil (air) | 6.5E-5 | kg |
|  | Emission | Nickel (air) | 2.57E-11 | kg |
|  | Emission | Phosphorus (air) | 3.07E-4 | kg |
|  | Emission | Silicon (air) | 1.43E-3 | kg |
|  | Emission | Zinc (air) | 1.42E-7 | kg |
|  | Emission | Aluminium (water) | 1.62E-2 | kg |
|  | Emission | Arsenic (water) | 3.63E-3 | kg |
|  | Emission | Cadmium (water) | 1.92E-4 | kg |
|  | Emission | Calcium (water) | 2.8 | kg |
|  | Emission | Chromium (water) | 8.75E-7 | kg |
|  | Emission | Copper (water) | 1.27E-4 | kg |
|  | Emission | Iron (water) | 7.7E-2 | kg |
|  | Emission | Lead (water) | 4.02E-5 | kg |
|  | Emission | Mercury (water) | 2.2E-5 | kg |
|  | Emission | Nickel (water) | 99.15E-4 | kg |
|  | Emission | Phosphate (water) | 0.5 | kg |
|  | Emission | Silicon (water) | 3.92E-2 | kg |
|  | Emission | Potassium | 0.9 | kg |
|  | Emission | Sulfate | 1.95 | kg |
|  | Emission | Zinc | 3.89E-3 | kg |
| Land application  (CS2) | Input | Dewatered sludge | 0.8 | t |
|  | Input | Transportation | 64 | tkm |
|  | Output | K-fertilizer (potassium nitrate) | 3.6 | kg |
|  | Output | N_fertilizer (calcium ammonium nitrate) | 8.8 | kg |
|  | Output | P-fertilizer (diammonium phosphate) | 8.95 | kg |
|  | Emission | Cadmium (soil) | 2.4E-3 | kg |
|  | Emission | Mercury (soil) | 2.4E-3 | kg |
|  | Emission | Lead (soil) | 0.24 | kg |
|  | Emission | Chromium (soil) | 0.4 | kg |
|  | Emission | Arsenic (soil) | 2.4E-2 | kg |
|  | Emission | Nickel (soil) | 8E-2 | kg |
|  | Emission | Zinc (soil) | 0.96 | kg |
|  | Emission | Copper (soil) | 0.4 | kg |
|  | Emission | Mineral oil | 0.4 | kg |
|  | Emission | Benzo(a)pyrene | 1.6E-3 | kg |
|  | Emission | PAHs | 4.8E-3 | kg |
|  | Emission | Ammonia (air) | 4.08 | kg |
|  | Emission | Dinitrogen oxide (air) | 0.12 | kg |
|  | Emission | Nitrogen oxides (air) | 4.56E-2 | kg |
|  | Emission | Carbon monoxide (air) | 3.12E-2 | kg |
|  | Emission | Particulates (air) | 6.83E-5 | kg |
|  | Emission | Phosphate (water) | 4.56E-2 | kg |
|  | Emission | Nitrate (water) | 0.6 | kg |
|  | Emission | Cadmium (water) | 2.18E-6 | kg |
|  | Emission | Copper (water) | 1.97E-4 | kg |
|  | Emission | Zinc (water) | 1.75E-3 | kg |
|  | Emission | Lead (water) | 2.83E-5 | kg |
|  | Emission | Chromium (water) | 1.14E-3 | kg |
|  | Emission | Mercury (water) | 7.12E-8 | kg |
| Dewatering (CS3) | Input | Thickening sludge | 1 | t |
|  | Input | Electricity | 10.53 | kWh |
|  | Input | Polyacrylamide  (flocculant) | 4 | kg |
|  | Output | Dewatered sludge | 1 | t |
| Composting (CS3) | Input | Dewatered sludge | 1 | t |
|  | Input | Electricity | 534 | kWh |
|  | Input | Diesel | 9.6 | kg |
|  | Output | Composting sludge | 0.81 | t |
|  | Output | K-fertilizer | 7.35 | kg |
|  | Output | N_fertilizer | 7.35 | kg |
|  | Output | P-fertilizer | 7.35 | kg |
|  | Emission | Methane | 8.2E4 | kg |
|  | Emission | Dinitrogen oxide | 6.98E-6 | kg |
|  | Emission | Carbon dioxide | 157.18 | kg |
| Land application  (CS3) | Input | Composting sludge | 0.81 | t |
|  | Input | Transportation | 16.2 | tkm |
|  | Emission | Cadmium (soil) | 7.45E-4 | kg |
|  | Emission | Mercury (soil) | 1.78E-5 | kg |
|  | Emission | Lead (soil) | 1.5E-2 | kg |
|  | Emission | Chromium (soil) | 3.9E-2 | kg |
|  | Emission | Nickel (soil) | 2.4E-2 | kg |
|  | Emission | Zinc Soil) | 0.34 | kg |
|  | Emission | Copper (soil) | 6.5E-2 | kg |
|  | Emission | Methane | 3.7E-2 | kg |
|  | Emission | Ammonia | 1.7 | kg |
|  | Emission | Dinitrogen oxide | 0.7 | kg |

**Table S6.** Results of life cycle inventories of the sludge treatment and disposal scenarios used in South Africa. The values are displayed per functional unit of 1 t dry sludge

| Processes | Types | Flows | Quantity | Unit |
| --- | --- | --- | --- | --- |
| Thickening  (SS1–3) | Input | Raw sludge | 1 | t |
|  | Input | Electricity | 14 | kWh |
|  | Input | Polyacrylamide (flocculant) | 4 | kg |
|  | Output | Thickening sludge | 1 | t |
| Anaerobic digestion  (SS1) | Input | Thickening sludge | 1 | t |
|  | Input | Heat | 1831.38 | MJ |
|  | Input | Electricity | 1.95 | kWh |
|  | Emission | Methane | 2.68 | kg |
|  | Emission | Carbon dioxide | 3.42 | kg |
|  | Emission | Nitrogen | 0.1 | kg |
|  | Emission | Hydrogen | 7.7E-3 | kg |
|  | Emission | Hydrogen sulfide | 4.5E-2 | kg |
|  | Output | Digested sludge | 0.72 | t |
|  | Output | Biogas | 185.94 | m^3^ |
| Cogeneration unit (SS1) | Input | Biogas | 185.94 | m^3^ |
|  | Input | Lubricating oil | 0.11 | kg |
|  | Output | Electricity | 483.44 | kWh |
|  | Output | Heat | 2175.22 | MJ |
|  | Output | Waste oil | 0.11 | kg |
|  | Emission | Carbon dioxide | 327.71 | kg |
|  | Emission | Methane | 1.31 | kg |
|  | Emission | Carbon monoxide | 1.22 | kg |
|  | Emission | Sulfur dioxide | 7.5E-2 | kg |
|  | Emission | Nitrogen oxides | 0.79 | kg |
|  | Emission | Dinitrogen oxides | 6.3E-3 | kg |
|  | Emission | Particulates | 1.3E-3 | kg |
| Dewatering (SS1) | Input | Digested sludge | 0.72 | t |
|  | Input | Electricity | 15.98 | kWh |
|  | Input | Polyacrylamide (flocculant) | 2.88 | kg |
|  | Output | Dewatered sludge | 0.72 | t |
| Land application  (SS1) | Input | Dewatered sludge | 0.72 | t |
|  | Input | Transportation | 54 | tkm |
|  | Output | N_fertilizer | 21.6 | kg |
|  | Output | P-fertilizer | 14.4 | kg |
|  | Output | K-fertilizer | 2.16 | kg |
|  | Emission | Cadmium (soil) | 6.12E-2 | kg |
|  | Emission | Mercury (soil) | 3.96E-2 | kg |
|  | Emission | Lead (soil) | 0.60 | kg |
|  | Emission | Chromium (soil) | 2.16 | kg |
|  | Emission | Arsenic (soil) | 5.4E-2 | kg |
|  | Emission | Nickel (soil) | 0.30 | kg |
|  | Emission | Zinc Soil) | 5.4 | kg |
|  | Emission | Copper (soil) | 3.10 | kg |
|  | Emission | Ammonia (air) | 3.67 | kg |
|  | Emission | Dinitrogen oxide (air) | 0.11 | kg |
|  | Emission | Nitrogen oxides (air) | 4.1E-2 | kg |
|  | Emission | PM (air) | 6.15E-5 | kg |
|  | Emission | Phosphate | 4.10E-2 | kg |
|  | Emission | Nitrate | 0.54 | kg |
|  | Emission | Cadmium (water) | 1.97E-6 | kg |
|  | Emission | Copper (water) | 1.77E-4 | kg |
|  | Emission | Zinc (water) | 1.58E-3 | kg |
|  | Emission | Lead (water) | 2.55E-5 | kg |
|  | Emission | Chromium (water) | 1.02E-3 | kg |
|  | Emission | Mercury (water) | 6.41E-8 | kg |
| Dewatering  (SS2 and SS3) | Input | Thickening sludge | 1 | t |
|  | Input | Electricity | 11.06 | kWh |
|  | Input | Polyacrylamide (flocculant) | 4 | kg |
|  | Output | Dewatered sludge | 1 | t |
| Thermal drying  (SS2 and SS3) | Input | Dewatered sludge | 1 | t |
|  | Input | Heat | 2784.68 | MJ |
|  | Input | Electricity | 45.65 | kWh |
|  | Output | Dried sludge | 1 | t |
| Incineration (SS2) | Input | Dried sludge | 1 | t |
|  | Input | NaOH | 10.79 | kg |
|  | Input | NaHCO_3_ | 33.9 | kg |
|  | Input | Lime | 66.15 | kg |
|  | Input | Activated carbon | 1.4 | kg |
|  | Input | Electricity | 485.33 | kWh |
|  | Output | Electricity | 2427.78 | kWh |
|  | Output | Heat | 8740 | MJ |
|  | Emission | Carbon monoxide | 3.5E-2 | kg |
|  | Emission | Nitrogen oxide | 0.35 | kg |
|  | Emission | Ammonia | 2.1E-2 | kg |
|  | Emission | Sulfur dioxide | 2.1E-2 | kg |
|  | Emission | PM | 2E-2 | kg |
| Landfill (SS2) | Input | Incinerated sludge | 0.3 | t |
|  | Input | Transportation | 4.5 | tkm |
|  | Emission | Aluminium (air) | 3.3E-4 | kg |
|  | Emission | Arsenic (air) | 6.96E-7 | kg |
|  | Emission | Cadmium (air) | 6.18E-9 | kg |
|  | Emission | Calcium (air) | 3.06E-4 | kg |
|  | Emission | Carbon monoxide, fossil | 1.36E-3 | kg |
|  | Emission | Chloride (air) | 6.9 | kg |
|  | Emission | Chromium (air) | 1.22E-11 | kg |
|  | Emission | Copper (air) | 8.37E-8 | kg |
|  | Emission | Iron (air) | 3.99E-5 | kg |
|  | Emission | Lead (air) | 7.26E-9 | kg |
|  | Emission | Mercury (air) | 7.05E-11 | kg |
|  | Emission | Methane, fossil (air) | 3.9E-5 | kg |
|  | Emission | Nickel (air) | 1.54E-11 | kg |
|  | Emission | Phosphorus (air) | 1.84E-4 | kg |
|  | Emission | Silicon (air) | 8.55E-4 | kg |
|  | Emission | Zinc (air) | 8.52E-8 | kg |
|  | Emission | Aluminium (water) | 9.72E-3 | kg |
|  | Emission | Arsenic (water) | 2.18E-3 | kg |
|  | Emission | Cadmium (water) | 1.15E-4 | kg |
|  | Emission | Calcium (water) | 1.68 | kg |
|  | Emission | Chromium (water) | 5.25E-7 | kg |
|  | Emission | Copper (water) | 7.59E-5 | kg |
|  | Emission | Iron (water) | 4.6E-2 | kg |
|  | Emission | Lead (water) | 2.41E-5 | kg |
|  | Emission | Mercury (water) | 1.32E-5 | kg |
|  | Emission | Nickel (water) | 5.49E-4 | kg |
|  | Emission | Phosphate (water) | 0.3 | kg |
|  | Emission | Silicon (water) | 2.35E-2 | kg |
|  | Emission | Potassium | 0.54 | kg |
|  | Emission | Sulfate | 1.17 | kg |
|  | Emission | Zinc | 2.33E-3 | kg |
| Land application  (SS3) | Input | Dried sludge | 1 | t |
|  | Input | Transportation | 75 | tkm |
|  | Output | K-fertilizer (potassium nitrate) | 3 | kg |
|  | Output | N_fertilizer (calcium ammonium nitrate) | 30 | kg |
|  | Output | P-fertilizer (diammonium phosphate) | 20 | kg |
|  | Emission | Cadmium (soil) | 8.5E-2 | kg |
|  | Emission | Mercury (soil) | 5.5E-2 | kg |
|  | Emission | Lead (soil) | 0.84 | kg |
|  | Emission | Chromium (soil) | 3 | kg |
|  | Emission | Arsenic (soil) | 7.5E-2 | kg |
|  | Emission | Nickel (soil) | 0.42 | kg |
|  | Emission | Zinc (soil) | 7.5 | kg |
|  | Emission | Copper (soil) | 4.3 | kg |
|  | Emission | Ammonia (air) | 5.1 | kg |
|  | Emission | Dinitrogen oxide (air) | 0.15 | kg |
|  | Emission | Nitrogen oxides (air) | 5.7E-2 | kg |
|  | Emission | Carbon monoxide (air) | 3.9E-2 | kg |
|  | Emission | Particulates (air) | 8.54E-5 | kg |
|  | Emission | Phosphate (water) | 5.7E-2 | kg |
|  | Emission | Nitrate (water) | 0.75 | kg |
|  | Emission | Cadmium (water) | 2.73E-6 | kg |
|  | Emission | Copper (water) | 2.46E-4 | kg |
|  | Emission | Zinc (water) | 2.19E-3 | kg |
|  | Emission | Lead (water) | 3.54E-5 | kg |
|  | Emission | Chromium (water) | 1.42E-3 | kg |
|  | Emission | Mercury (water) | 8.9E-8 | kg |

# References

Technology standard for sludge treatment of municipal wastewater treatment plant Housing and Urban-Rural Development

CNSMC. 2018 Control standards of pollutants in sludge for agricultural use, China National Standardization Management Committee.

Alengebawy, A., Mohamed, B.A., Ghimire, N., Jin, K., Liu, T., Samer, M. and Ai, P. 2022. Understanding the environmental impacts of biogas utilization for energy production through life cycle assessment: An action towards reducing emissions. Environ. Res. 213, 113632.

Apollo, S. 2022. A review of sludge production in South Africa municipal wastewater treatment plants, analysis of handling cost and potential minimization methods. Phy. Sci. Rev.

Appels, L., Baeyens, J., Degrève, J. and Dewil, R. 2008. Principles and potential of the anaerobic digestion of waste-activated sludge. Progress in energy and combustion science 34(6), 755-781.

Avancini, L.L.d.S., Muller, M. and Vidal, D.d.M. 2021. Application of polyelectrolytes for improving the dewatering performance of drinking water treatment sludge using geotextiles. Rev. Ambient. Água 16.

Bittencourt, S. 2018. Agricultural use of sewage sludge in Paraná State, Brazil: A decade of national regulation. Recycling 3(4), 53.

Cañote, S.J.B., Barros, R.M., Lora, E.E.S., dos Santos, I.F.S., Silva, A.P.M., Piñas, J.A.V., Cañote, A.L.B. and de Castro e Silva, H.L. 2021. Life cycle assessment of upflow anaerobic sludge blanket sludge management and activated sludge systems aiming energy use in the municipality of Itajubá, Minas Gerais, Brazil. J. Mater. Cycles Waste Manag. 23, 1810-1830.

Chai, C., Zhang, D., Yu, Y., Feng, Y. and Wong, M.S. 2015. Carbon footprint analyses of mainstream wastewater treatment technologies under different sludge treatment scenarios in China. Water 7(3), 918-938.

Chen, S., Huang, J., Xiao, T., Gao, J., Bai, J., Luo, W. and Dong, B. 2020. Carbon emissions under different domestic waste treatment modes induced by garbage classification: Case study in pilot communities in Shanghai, China. Sci. Total Environ. 717, 137193.

Diaz-Elsayed, N., Rezaei, N., Ndiaye, A. and Zhang, Q. 2020. Trends in the environmental and economic sustainability of wastewater-based resource recovery: A review. J. Clean. Prod. 265, 121598.

do Amaral, K.C., Aisse, M.M., Possetti, G.R.C. and Prado, M.R. 2018. Use of life cycle assessment to evaluate environmental impacts associated with the management of sludge and biogas. Water Sci. Technol. 77(9), 2292-2300.

Ghosh, P., Kumar, M., Kapoor, R., Kumar, S.S., Singh, L., Vijay, V., Vijay, V.K., Kumar, V. and Thakur, I.S. 2020. Enhanced biogas production from municipal solid waste via co-digestion with sewage sludge and metabolic pathway analysis. Bioresour. Technol. 296, 122275.

Guimarães, M. and Urashima, D. 2013. Dewatering sludge in geotextile closed systems: brazilian experiences. Soils Rocks 36(3), 251-263.

Hao, X., Li, J., van Loosdrecht, M.C.M., Jiang, H. and Liu, R. 2019. Energy recovery from wastewater: Heat over organics. Water Res. 161, 74-77.

Huang, Y., Zhen, Y., Liu, L., Ning, X., Wang, C., Li, K., Zhao, L. and Lu, Q. 2023. Comprehensive competitiveness assessment of four typical municipal sludge treatment routes in China based on environmental and techno-economic analysis. Sci. Total Environ., 165123.

Kalyuzhnyi, S. 2007. Wastewater sludge management in the Russian federation: the current status and perspectives. Water Pract. Technol. 2(4), wpt2007085.

Kashkovsky, V., Evdokymenko, V., Kamensky, D. and Evdokymenko, A. 2014. Method of sewage sludge dewatering with the use of geotube technique elements at the bortnichy aeration station. Science 10(1), 30-40.

Kiselev, A., Magaril, E., Magaril, R., Panepinto, D., Ravina, M. and Zanetti, M.C. 2019. Towards circular economy: Evaluation of sewage sludge biogas solutions. Resources 8(2), 91.

Kovalev, A.A., Mikheeva, E.R., Kovalev, D.A., Katraeva, I.V., Zueva, S., Innocenzi, V., Panchenko, V., Zhuravleva, E.A. and Litti, Y.V. 2022. Feasibility Study of Anaerobic Codigestion of Municipal Organic Waste in Moderately Pressurized Digesters: A Case for the Russian Federation. Appl. Sci. 12(6), 2933.

Kulikowska, D. and Gusiatin, Z.M. 2015. Sewage sludge composting in a two-stage system: Carbon and nitrogen transformations and potential ecological risk assessment. Waste Manage. 38, 312-320.

Kumar, A., Bhardwaj, S. and Samadder, S.R. 2023. Evaluation of methane generation rate and energy recovery potential of municipal solid waste using anaerobic digestion and landfilling: A case study of Dhanbad, India. Waste Manage. Res. 41(2), 407-417.

LeBlanc, R.J., Matthews, P. and Richard, R.P. (2009) Un-habitat.

Li, Y., Luo, X., Huang, X., Wang, D. and Zhang, W. 2013. Life cycle assessment of a municipal wastewater treatment plant: a case study in Suzhou, China. J. Clean. Prod. 57, 221-227.

Mannarino, G., Caffaz, S., Gori, R. and Lombardi, L. 2022. Environmental life cycle assessment of hydrothermal carbonization of sewage sludge and its products valorization pathways. Waste Biomass Valori. 13(9), 3845-3864.

Mayer, F., Bhandari, R. and Gäth, S.A. 2021. Life cycle assessment of prospective sewage sludge treatment paths in Germany. J. Environ. Manag. 290, 112557.

Medeiros, D.L., Dos Santos, C.M.Q., Ribeiro, R. and Tommaso, G. 2023. The dissolved methane recovery from treated sewage in upflow anaerobic sludge blanket (UASB) reactors: The energy demand, carbon footprint and financial cost. J. Environ. Manage. 343, 118258.

Medina-Martos, E., Istrate, I.-R., Villamil, J.A., Gálvez-Martos, J.-L., Dufour, J. and Mohedano, Á.F. 2020. Techno-economic and life cycle assessment of an integrated hydrothermal carbonization system for sewage sludge. J. Clean. Prod. 277, 122930.

Mukawa, J., Pająk, T., Rzepecki, T. and Banaś, M. 2022. Energy potential of biogas from sewage sludge after thermal hydrolysis and digestion. Energies 15(14), 5255.

Patel, K. and Singh, S.K. 2022. A life cycle approach to environmental assessment of wastewater and sludge treatment processes. Water Environ. J. 36(3), 412-424.

Ravi, R., Beyers, M., Bruun, S. and Meers, E. 2022. Life cycle assessment of struvite recovery and wastewater sludge end-use: A Flemish illustration. Resour. Conserv. Recycl. 182, 106325.

Saha, S., Hazra, G., Saha, B. and Mandal, B. 2015. Assessment of heavy metals contamination in different crops grown in long-term sewage-irrigated areas of Kolkata, West Bengal, India. Environ. Monit. Assess. 187, 1-12.

Saha, S., Saha, B.N., Pati, S., Pal, B. and Hazra, G.C. 2017. Agricultural use of sewage sludge in India: benefits and potential risk of heavy metals contamination and possible remediation options–a review. Int. J. Environ. Technol. Manage. 20(3-4), 183-199.

Singh, A.D., Upadhyay, A., Shrivastava, S. and Vivekanand, V. 2020a. Life-cycle assessment of sewage sludge-based large-scale biogas plant. Bioresour. Technol. 309, 123373.

Singh, V., Phuleria, H.C. and Chandel, M.K. 2020b. Estimation of energy recovery potential of sewage sludge in India: Waste to watt approach. J. Clean. Prod. 276, 122538.

Tarpani, R.R.Z. and Azapagic, A. 2018. Life cycle costs of advanced treatment techniques for wastewater reuse and resource recovery from sewage sludge. J. Clean. Prod. 204, 832-847.

Tauber, J., Parravicini, V., Svardal, K. and Krampe, J. 2019. Quantifying methane emissions from anaerobic digesters. Water Sci. Technol. 80(9), 1654-1661.

Tesfamariam, E.H., Ogbazghi, Z.M., Annandale, J.G. and Gebrehiwot, Y. 2020. Cost–benefit analysis of municipal sludge as a low-grade nutrient source: A case study from South Africa. Sust. 12(23), 9950.

Tyagi, V.K., Kapoor, A., Arora, P., Banu, J.R., Das, S., Pipesh, S. and Kazmi, A.A. 2021. Mechanical-biological treatment of municipal solid waste: Case study of 100 TPD Goa plant, India. J. Environ. Manage. 292, 112741.

Värri, H., Havukainen, J. and Horttanainen, M.H. 2010. Suitability of wastewater sludge for utilization in the Leningrad Region (Russia). Linnaeus Eco-Tech, 188-196.

Waal, C.v.d. 2008 Guidelines for the Utilisation and Disposal of Wastewater Sludge

Wang, Z., Li, X., Siddiqui, M.A., Liu, H., Zhou, T., Zheng, L., Huang, S., Gao, L., Lin, C.S.K. and Wang, Q. 2023. Effect of humic substances on the anaerobic digestion of secondary sludge in wastewater treatment plants: a review. Environ. Chem. Lett., 1-18.

Xu, C., Chen, W. and Hong, J. 2014. Life-cycle environmental and economic assessment of sewage sludge treatment in China. J. Clean. Prod. 67, 79-87.

Xu, Y., Liu, R., Liu, H., Geng, H. and Dai, X. 2022. Novel anaerobic digestion of waste activated sludge via isoelectric-point pretreatment: Ultra-short solids retention time and high methane yield. Water Res. 220, 118657.

Yang, G., Zhang, G. and Wang, H. 2015. Current state of sludge production, management, treatment and disposal in China. Water Res. 78, 60-73.

Zhao, S., Chen, W., Luo, W., Fang, H., Lv, H., Liu, R. and Niu, Q. 2021. Anaerobic co-digestion of chicken manure and cardboard waste: Focusing on methane production, microbial community analysis and energy evaluation. Bioresour. Technol. 321, 124429.

Zhao, S., Yan, K., Wang, Z., Gao, Y., Li, K. and Peng, J. 2023. Does anaerobic digestion improve environmental and economic benefits of sludge incineration in China? Insight from life-cycle perspective. Resour. Conserv. Recy. 188, 106688.

Zhuang, Z. (2021) Environmental and economic life cycle assessment of sewage sludge treatment processes, University of British Columbia.
